# Dynamic clonal hematopoiesis and functional T-cell immunity in a supercentenarian

**DOI:** 10.1038/s41375-020-01086-0

**Published:** 2020-11-12

**Authors:** Erik B. van den Akker, Stavros Makrodimitris, Marc Hulsman, Martijn H. Brugman, Tatjana Nikolic, Ted Bradley, Quinten Waisfisz, Frank Baas, Marja E. Jakobs, Daphne de Jong, P. Eline Slagboom, Frank J. T. Staal, Marcel J. T. Reinders, Henne Holstege

**Affiliations:** 1 Leiden Computational Biology Center, Leiden University Medical Center, Leiden, The Netherlands; 2 Section of Molecular Epidemiology, Leiden University Medical Center, Leiden, The Netherlands; 3 Pattern Recognition & Bioinformatics, Delft University of Technology, Delft, The Netherlands; 4 Department of Clinical Genetics, Vrije Universiteit Amsterdam, Amsterdam University Medical Center, Amsterdam, The Netherlands; 5 Alzheimer Center Amsterdam, Department of Neurology, Amsterdam Neuroscience, Vrije Universiteit Amsterdam, Amsterdam UMC, Amsterdam, The Netherlands; 6 Department of Immunology, Leiden University Medical Center, Leiden, The Netherlands; 7 Department of Clinical Genetics, Leiden University Medical Center, Leiden, The Netherlands; 8 Department of Clinical Genetics, Academic Medical Center, Amsterdam University Medical Center, Amsterdam, The Netherlands; 9 Department of Pathology, Vrije Universiteit Amsterdam, Amsterdam University Medical Center, Amsterdam, The Netherlands

**Keywords:** Genomics, T cells, Haematopoiesis, Haematopoietic stem cells

## To the Editor:

Age-related Clonal hematopoiesis (ARCH) is an inevitable consequence of ageing, which arises when an ageing hematopoietic stem cell (HSC) acquires a somatic mutation that confers a competitive growth advantage, leading to its gradual expansion [1]. ARCH-associated mutations typically target genes associated with acute myeloid leukemia, most frequently the epigenetic regulators *DNMT3A* and *TET2* [2]. When a substantial proportion of the blood cells carries such a pre-leukemic mutation in an otherwise normal immuno-hematopoietic system, this state is also referred to as Clonal Hematopoiesis of Indeterminate Potential [3].

Next to its role in acute myeloid leukemia, ARCH has also been associated with a broad spectrum of age-related low-grade inflammatory syndromes [4], including type 2 diabetes, chronic obstructive pulmonary disease, cardiovascular disease, and all-cause mortality. Somatic mutations accumulate in the HSC over the course of a lifetime [5], thus effectively tagging each individual HSC and its offspring with a unique “genetic barcode” [1]. Acquired somatic mutations are heterozygous, and because a mutated clone contributes to only a fraction of the total peripheral blood, somatic mutations have an allele balance (between alternate allele and reference allele) that is consistently lower than the 1:1 ratio observed for germline heterozygous mutations. The variant allele frequency (VAF) of each somatic mutation is representative of the fraction of blood cells generated by the HSC that carries the variant/mutation.

By applying this paradigm, we previously found that ~65% of the peripheral blood from a healthy 115-year-old female (W115) was derived from a single HSC [6]. Finding extensive ARCH in a healthy 115-year-old was unexpected given the association between ARCH and all-cause mortality, but it was in line with our previous observations that the association between ARCH and all-cause mortality seems to wane in the oldest old [7]. Together, our findings led us to question whether the ARCH was recently established prior to the 115-year-old’s death, or whether it had taken many years for the mutated HSC to gradually populate the majority of her peripheral blood. Also, we questioned whether the mutated HSC contributed to all blood cell types or to specific blood cell types. Furthermore, it was unclear to what extent immune function was compromised in a hematopoietic system dominated by a single mutated HSC clone.

**Fig. 1:**
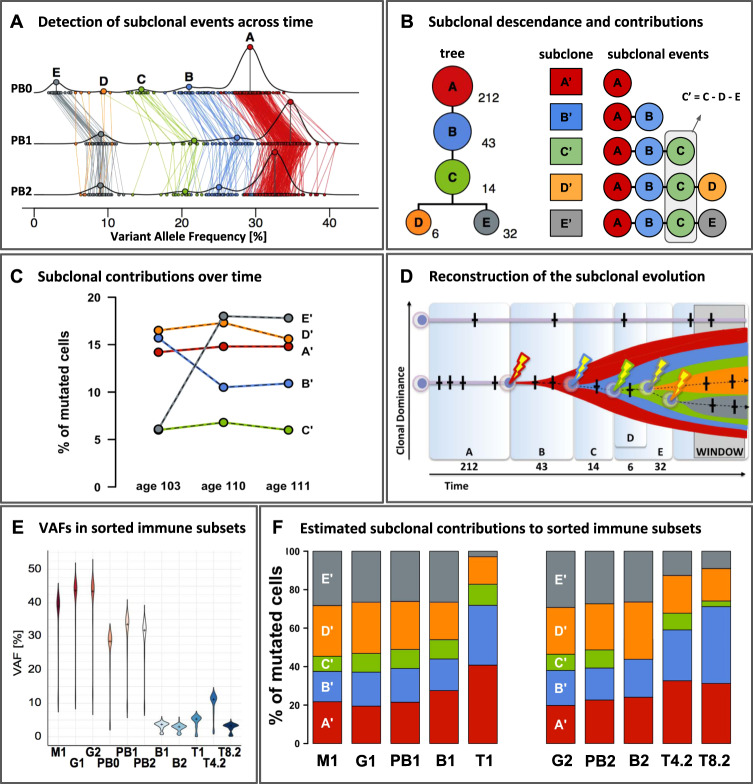
Deep sequencing of longitudinal samples reveals the clonal architecture within the peripheral blood of an elderly subject with age-related clonal hematopoiesis. Blood samples from W111 were collected at three time points, age 103 (timepoint 0), 110 (timepoint 1), and 111 (timepoint 2) respectively, and included peripheral blood (PB0, PB1, PB2), as well as its flow sorted subsets: granulocytes (G), monocytes (M), T-cells (T), CD4^+^ T-cells (T4), CD8^+^ T-cells (T8) and B-cells (B). Numbers signify time points 0, 1 and 2. **A** Horizontal lines PB0, PB1, PB2: the density distribution of the variant allele frequency (VAF) of the 307 confirmed somatic mutations at ages 103, 110 and 111. With clone A as the founding clone, the median VAF of variants in clone A represents 0.5x the median contribution of the clone and its subclones to the peripheral blood. Colored lines connect the same mutations measured at the different timepoints. Using SciClone, mutations were assigned to five independent clonal events (A-E) and colored accordingly. See supplement for in-depth methods and results. **B** Left: Modeling with SCHISM indicated that these five clonal events most likely occurred consecutively within a single clonal lineage that terminates into two independent sister-clones D and E. These were derived from a shared ancestral subclone carrying mutations associated with clonal events (**A**–**C**). The number of somatic mutations supporting each subclonal event are listed next to the clones. Right: to estimate the contributions of each subclone to peripheral blood, we corrected for the interdependencies introduced by the shared clonal descendance: all somatic mutations in clone A are present in its clonal descendants (**B**–**E**), and all somatic mutations in (**B**) are present in (**C**–**E**), but not (**A**). **C** Median VAFs after subtractions of the median VAF of the descendant clonal event indicates that changes in dominance of subclones A-C are largely explained by changes in dominance of subclone E and notably not by subclone D. **D** Reconstruction of subclonal evolution. Time frames A–E correspond to the periods in which passenger mutations (crosses) were accumulated until a clonal event driving expansion (bolt) was encountered. Widths of the time frames are roughly proportional to the number of mutations detected for each event. The y-axis reflects the relative contribution of an HSC to overall peripheral blood production. ‘WINDOW’ refers to our window of observation ranging from age 103 to 111, a 9-year period characterized by the expansion of clonal event E. **E** Violin plots of VAFs [%] in peripheral blood and its sorted subsets. The amplicon panel of 307 somatic mutations were used to re-sequence DNA derived from FACS-sorted immune subsets. The median VAFs between different cell subsets collected at age 110 indicated a significant higher clonal contribution to the myeloid branch (87.4% of the granulocytes (G1_VAF_ = 0.437) and 77.8% of the monocytes (M1_VAF_ = 0.389)) compared to the lymphoid lineage (~10.6% of the T-cells (T1_VAF_ = 0.053), and ~7.4% of the B-cells (B1_VAF_ = 0.037)). Re-sequencing within the blood sample collected at age 111 indicated that VAFs were significantly higher in CD4^+^ T-cells (22.2% of the cells, T4.2_VAF_ = 0.111) compared to CD8^+^ T-cells (6.4% of the cells, T8.2_VAF_ = 0.032, *p* < 0.001, Wilcoxon) and B-cells (6.0% of the cells, B2_VAF_ = 0.030, *p* < 0.001, Wilcoxon). **F** Fraction of mutated cells per sorted cell subset derived from each subclone. Stacked bar plots per subset add up to 100%.

To address these questions, we investigated ARCH in a second centenarian female who died at 111 years (W111), and whose blood showed no signs of hematological malignancies. This time, we investigated ARCH longitudinally, using W111-blood samples collected at ages 103, 110, and 111 years. By comparing deep sequencing data from DNA derived from blood and a skin biopsy collected at age 110, we identified 650 putative somatic mutations that were present in blood and absent in skin (see supplement for methods/results/mutation-lists). For a subset of these mutations we successfully designed a targeted amplicon resequencing panel which allowed us to confirm the somatic origin of 307 mutations. These mutations served as genetic markers for clonal tracing in blood samples taken at different time points or different sorted immune subsets. The density distribution of the VAF of these 307 somatic mutations exhibited multiple peaks, indicative of a subclonal architecture (Fig. 1A, line PB1). Lineage analysis inferred five clonal events within a single clonal lineage in which a founding clone A brought forth subclone B, which brought forth subclone C, from which two independent sister-clones D and E originated (Fig. 1B). Based on the VAF distribution of variants assigned to founding clone A (and thereby to subclones B-E) we determined that at age 110, ~70–75% of the peripheral blood was generated by the clone and its subclones (Fig. 1A, line PB1).

We screened the somatic mutations for candidate driver mutations using the definitions compiled by Jaiswal et al. [2], and identified and confirmed a splice-donor site mutation in intron 11 of DNA (cytosine-5)-methyltransferase 3 A (*DNMT3A*, NM_022552.4, chr2:25,469,028_C > T, c.1429 + 1 G > A), which was previously observed in patients with hematopoietic or lymphoid malignancies (COSM5945645). Based on its VAF of 0.38 the *DNMT3A* mutation was assigned to founding clone A, such that this mutation may have driven the initial clonal expansion [8]. Among the remaining somatic mutations, we did not identify putative driver mutations that could explain the successive subclonal expansions B-E, possibly due to undetected or incomplete knowledge of driver mutations [1]. Alternatively, impairment of *DNMT3A* function, a key epigenetic regulator, may lead to ‘epimutations’ that improve the replicatory fitness of an HSC [9]. Also, the *DNMT3A* mutation may lead to enhanced HSC-proliferation upon bone marrow stress, such as inflammation or environmental stimuli. In such a scenario, the clonal architecture could represent the history of reactivation of otherwise quiescent HSCs [10].

To explore whether the contribution of each subclone to the peripheral blood changed over time, we investigated the VAFs of the 307 mutations identified at age 110 years in the peripheral blood samples collected at ages 103 and 111. VAFs were inter-correlated between timepoints (Fig. 1A). The inferred clonal lineage implies that all somatic mutations in clone A are present in its clonal descendants B-E, and all somatic mutations in B are present in C-E, but not A (Fig. 1B). After adjusting for these interdependencies, we observed that temporal changes in dominance of subclones A-C are largely explained by changes in dominance of subclone E (Fig. 1C). While clonal event D exhibits a near equal contribution of ~16.5% of the cells to peripheral blood at age 103, 110, and 111 years, clonal event E nearly tripled its clonal contribution from 6.1% at age 103–17.9% of the peripheral blood cells between ages 110 and 111. Meanwhile, clone B becomes less dominant: its contribution to peripheral blood decreases from 15.7% at age 103 to 10.7% at ages 110 and 111. Concluding, we observed a complex subclonal architecture with ongoing dynamics during the 9-year timeframe of our sampling, and we reconstructed a possible path of subclonal evolution (Fig. 1D).

Next, we investigated to what extent the somatic mutations were present in the major cell subsets of peripheral blood sampled at age 110 (PB1) and age 111 (PB2). We observed that the *DNMT3A*-mutated HSC contributed to the majority of the myeloid cells (78–87%) and to a small proportion of T-cells (11%) and B-cells (6–7%) (Fig. 1E). Moreover, the HSC contributed to a significantly larger proportion of CD4^+^ T-cells (22%) than CD8^+^ T-cells (6%). We also observed differences between the subclonal contributions to cell subsets (Fig. 1F). Specifically, subclones A and B generated a disproportionally high fraction of T-cells, while subclone E generated a disproportionally low fraction. The necessity to continuously regenerate short-lived myeloid cells may lead to a myeloid bias in the offspring generated by the newer subclones [11]. T-cells, however, are known to live tens of years, to uphold long-term immunity against specific antigens. Therefore, T-cells generated by older clones, possibly years prior to sampling, may lead to a relative higher contribution of the active HSC-clone to T-cells.

**Fig. 2:**
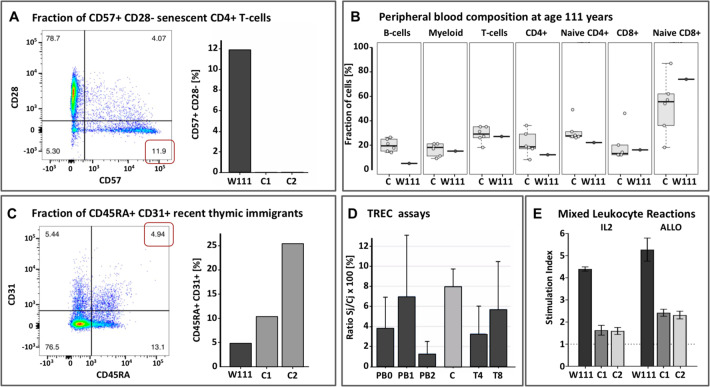
Immune characterization of W111. **A** Left: Flow cytometry Sorting of peripheral blood taken at age 110 showed increased fractions of CD57^+^ CD28^-^ senescent CD4^+^ and CD8^+^ T-cells relative to middle aged controls, as apparent by the expression of CD57; Right: Proportion of senescent cells in W111 compared to middle-aged controls C1 and C2. **B** Proportions of sorted immune subsets (B-cells, Myeloid, T-cells, CD4^+^ T-cells, naive CD4^+^ T-cells, CD8^+^ T-cells, naive CD8^+^ T-cells) in peripheral blood of six middle-aged female controls (left) in W111 at age 111 years (right). **C**. Left: At age 110 years, nearly 5% of the CD4^+^ T-cells expressed both CD45RA and CD31, indicative of recent thymic emigrants; Right: The level of recent thymic emigrants in W111 was compared to middle-aged controls C1 and C2 [**D**] The percentage of T-cell receptor excision circles (TRECs) in in W111’s peripheral blood and sorted CD4^+^ and CD8^+^ T-cells at age 110 and 111 years was comparable to that of middle-aged healthy female controls C (3–6%). PB: Peripheral Blood cells; T4: CD4^+^ T-cells; T8: CD8^+^ T-cells. Numbers signify time points: 0: age 103; 1: age 110; 2: age 111. **E** In vitro proliferation assays: we computed Stimulation Indices for an IL2/TCR-dependent (IL2) and an allogeneic mixed-lymphocyte assay (ALLO) assay of cultured T-cells of W111 and two middle-aged female controls C1 and C2. In both assays, T-cells collected from W111 outperformed those taken from middle-aged controls on a per cell basis. Furthermore, see Supplement for flow cytometry analyses of cells collected at age 110 and 111, which indicated that both the CD4^+^ and CD8^+^ T-cell subsets contained considerable fractions of in vivo activated cells, evidenced by their high CD25 expression and CD69 expression.

Ageing of the T-cell compartment, immune-senescence, has been postulated as a major factor underlying a reduced life expectancy [12]. Indeed, W111’s peripheral blood shows clear signs of an aged immune system: using flow cytometry, we found increased fractions of senescent CD4^+^ and CD8^+^ T-cells relative to middle aged controls (Fig. 2A and Supplement), and a myeloid shift, (i.e., high myeloid to B lymphocyte ratios), particularly due to lowered B-cell levels (Fig. 2B). However, we were surprised to find that at ages 110 and 111 years, the fraction of naive CD4^+^ T-cells was only slightly decreased and that the fraction of naive CD8^+^ T-cells was comparable relative to middle aged controls (Fig. 2B). Moreover, at age 110 years, we observed recent thymic emigrants in W111’s peripheral blood, although at lower levels compared to middle aged controls (Fig. 2C) [13]. Furthermore, while T-cell proliferation is often undetectable after 85 years [14], we detected ongoing T-cell proliferation in the peripheral blood of W111 at levels comparable to that of middle-aged healthy controls (Fig. 2D). In vivo and in vitro proliferation assays confirmed that W111 had preserved the capability of mounting a vigorous naive T cell response (Fig. 2E and Supplement). We found that W111 still demonstrated functional T-cell immunity, which may have, at least in part, contributed to her extreme longevity. The marked contribution (~22%) of the stem cell clone to the CD4^+^ T-cell subset (Fig. 1E) combined with well-preserved T cell immunity led us to question whether in some individuals, ARCH may be a benign consequence of aging. These findings are in line with a recent report by Hashimoto et al. [15], who observed clonally expanded CD4^+^ T-cells in 7 supercentenarians. While Hashimoto et al. attributed this clonal T-cell expansion to a sustained antigenic stimulation, our findings suggest benign ARCH as a competing explanation. In fact, we can take this one step further and speculate that ARCH may actually contribute to maintaining a functional T-cell immunity.

We acknowledge that our observations in a single healthy supercentenarian preclude any inference of causality between ARCH and an unexpectedly functional T-cell immunity. However, the presented findings warrant future research of ARCH in large cohorts of aged healthy individuals in relation to phenotypic and functional parameters of adaptive immunity.

## Supplementary information

Supplemental Material

## References

[1] Zink F, Stacey SN, Norddahl GL, Frigge ML, Magnusson OT, Jonsdottir I, et al. Clonal hematopoiesis, with and without candidate driver mutations, is common in the elderly. Blood. 2017; blood-2017-02-769869. 10.1182/blood-2017-02-769869. 10.1182/blood-2017-02-769869 PMC5553576 28483762

[2] Jaiswal S, Fontanillas P, Flannick J, Manning A, Grauman PV, Brenton Mar GB (2014). Age-related clonal hematopoiesis associated with adverse outcomes. N Engl J Med.

[3] Steensma DP, Bejar R, Jaiswal S, Coleman Lindsley R, Sekeres MA, Hasserjian RP (2015). Clonal hematopoiesis of indeterminate potential and its distinction from myelodysplastic syndromes. Blood..

[4] Bowman RL, Busque L, Levine RL. Clonal Hematopoiesis and Evolution to Hematopoietic Malignancies. Cell Stem Cell. 2018;22:157–70. 10.1016/j.stem.2018.01.011. 10.1016/j.stem.2018.01.011 PMC5804896 29395053

[5] Welch JS, Ley TJ, Link DC, Miller CA, Larson DE, Koboldt DC (2012). The origin and evolution of mutations in acute myeloid leukemia. Cell..

[6] Holstege H, Pfeiffer W, Sie D, Hulsman M, Nicholas TJ, Lee CC (2014). Somatic mutations found in the healthy blood compartment of a 115-yr-old woman demonstrate oligoclonal hematopoiesis. Genome Res.

[7] Van den Akker EB, Pitts SJ, Deelen J, Moed MH, Potluri S, van Rooij J (2016). Uncompromised 10-year survival of oldest old carrying somatic mutations in DNMT3A and TET2. Blood..

[8] Watson CJ, Papula AL, Poon GYP, Wing HW, Young AL, Druley TE (2020). The evolutionary dynamics and fitness landscape of clonal hematopoiesis. Science..

[9] Abdel-Wahab O, Levine RL (2013). Mutations in epigenetic modifiers in the pathogenesis and therapy of acute myeloid leukemia. Blood.

[10] Bystrykh LV, Verovskaya E, Zwart E, Broekhuis M, de Haan G (2012). Counting stem cells: methodological constraints. Nat Methods.

[11] Buscarlet M, Provost S, Feroz Zada Y, Vincent B, Mollica L, Dubé M (2018). Lineage restriction analyses in CHIP indicate myeloid bias for TET2 and multipotent stem cell origin for DNMT3A. Blood..

[12] Mekker A, S Tchang VS, Haeberli L, Oxenius A, Trkola A, Karrer U (2012). Immune senescence: relative contributions of age and cytomegalovirus infection. PLoS Pathog.

[13] Mitchell WA, Lang PO, Aspinall R (2010). Tracing thymic output in older individuals. Clin Exp Immunol.

[14] Nasi M, Troiano L, Lugli E, Pinti M, Ferraresi R, Monterastelli E (2006). Thymic output and functionality of the IL-7/IL-7 receptor system in centenarians: implications for the neolymphogenesis at the limit of human life. Aging Cell.

[15] Hashimoto K, Kouno T, Ikawa T, Hayatsu N, Miyajima Y, Yabukami H (2019). Single-cell transcriptomics reveals expansion of cytotoxic CD4 T-cells in supercentenarians. Proc Natl Acad Sci U S A.

