# Supplementary material for: Dynamic clonal hematopoiesis and functional T-cell immunity in a supercentenarian
Source: Leukemia. 2020 Nov 12;35(7):2125–9. doi: 10.1038/s41375-020-01086-0 (PMC8257492; doi:10.1038/s41375-020-01086-0)
Supplement: Supplementary file 1 — Supplemental Material [file 41375_2020_1086_MOESM1_ESM.docx]

# SUPPLEMENTARY MATERIAL

to

**DYNAMIC CLONAL HEMATOPOIESIS AND FUNCTIONAL T-CELL IMMUNITY IN A SUPER-CENTENARIAN**

Erik B. van den Akker^1,2,3+#^, Stavros Makrodimitris^3,5+^, Marc Hulsman^3,4,5^, Martijn H. Brugman^6^, Tatjana Nikolic^6^, Ted Bradley^4^, Quinten Waisfisz^4^, Frank Baas^7^, Marja E. Jakobs^8^, Daphne de Jong^9^, P. Eline Slagboom^2^, Frank J.T. Staal^6^, Marcel J.T. Reinders^1,3^, and Henne Holstege^3,4,5#^

Affiliations

1. Leiden Computational Biology Center, Leiden University Medical Center, Leiden, The Netherlands
2. Section of Molecular Epidemiology, Leiden University Medical Center, Leiden, The Netherlands
3. Pattern Recognition & Bioinformatics, Delft University of Technology, Delft, The Netherlands
4. Department of Clinical Genetics, Vrije Universiteit Amsterdam, Amsterdam UMC, Amsterdam, The Netherlands
5. Alzheimer Center Amsterdam, Department of Neurology, Amsterdam Neuroscience, Vrije Universiteit Amsterdam, Amsterdam UMC, Amsterdam, The Netherlands
6. Department of Immunology, Leiden University Medical Center, Leiden, The Netherlands
7. Department of Clinical Genetics, Leiden University Medical Center, Amsterdam, The Netherlands
8. Department of Clinical Genetics, Academic Medical Center, Amsterdam UMC, Amsterdam, The Netherlands
9. Department of Pathology, Amsterdam University Medical Center, Amsterdam, The Netherlands

+Equally contributed

#Correspondence: Henne Holstege, [h.holstege@amsterdamumc.nl](mailto:h.holstege@amsterdamumc.nl) and Erik B. van den Akker, [e.b.van_den_akker@lumc.nl](mailto:e.b.van_den_akker@lumc.nl)

**Material & correspondence**

Erik B. van den Akker, PhD; Leiden Computational Biology Center, Leiden University Medical Center, Leiden, The Netherlands; Einthovenweg 20, 2333 ZC, Leiden, The Netherlands; Tel: +31 (0)71 526 85 57; Fax: +31 (0)71 526 82 80; E-mail: [e.b.van_den_akker@lumc.nl](mailto:e.b.van_den_akker@lumc.nl); website:<http://www.lcbc.nl>

Henne Holstege, PhD; Amsterdam Alzheimer Center & Clinical Genetics, Amsterdam UMC—location VUmc, de Boelelaan 1118, 1081 HZ Amsterdam, The Netherlands; Tel: +31 (0)20 444 08 16; E-mail: [h.holstege@amsterdamumc.nl](mailto:h.holstege@amsterdamumc.nl); website: [www.100plus.nl](http://www.100plus.nl)

TABLE OF CONTENTS

**SUPPLEMENTARY METHODS - SM 3**

SM1: Study design and data collection 3

SM2: Clinical Assessment of the hematological status 3

SM3: Whole genome sequencing 3

SM4: Sommix 4

SM5: Amplicon re-sequencing 8

SM6: Identification of candidate driver mutations 8

SM7: Mutational signatures 9

SM8: Inferring the subclonal architecture 9

SM9: Immuno-phenotyping by flow cytrometry 9

SM10: T-cell Receptor Excision Circle (TREC) assays 9

SM11: Mixed Leukocyte Reactions (MLR) 9

**SUPPLEMENTARY RESULTS - SR 11**

SR1: Cataloguing somatic mutations in peripheral blood 11

SR2: Design of an amplicon assay for clonal tracing 11

**SUPPLEMENTARY FIGURES - SF 12**

SF1: Study design

SF2: Cataloguing somatic mutations in peripheral blood 12

SF3: Rainfall plot of 650 putative SNVs 13

SF4: Tri-nucleotide context of 650 putative SNVs 14

SF5: Mutational signatures contributing to 650 putative SNVs 15

SF6: Pairwise scatter plots of 307 SNVs at PB0, PB1 & PB2 19

SF7: Testing for suspect clonal BCR/TCR gene recombinations 20

SF8: T-cell characterization at age 110 by flow cytometry 24

SF9: T-cell characterization at age 111 by flow cytometry 33

**SUPPLEMENTARY TABLES - ST 34**

ST1: 650 Putative Somatic Mutations 34

ST2: Variant Allele Fractions of 307 Validated Somatic Mutations 48

ST3: Candidate driver definitions 55

**SUPPLEMENTARY REFERENCES 57**

References 57

Supplementary Methods - SM

# SM1: Study design and data collection

Subject of this study was W111, a Dutch female who lived for 111 years and 10 months. W111 was enrolled as a participant of the Leiden Longevity Study^12^ and seven years later as a participant of the 100-plus Study^13^. 100-plus Study and Leiden Longevity Study have been approved by their respective local Medical Ethical Committees and all participants, including the current subject, gave informed consent for study participation.

Blood samples from W111 were collected at three time points, age 103 (time point 0), 110 (time point 1), and 111 (time point 2) respectively (**Figure SF1**), and included peripheral blood (PB), its flow sorted subsets: granulocytes (G), monocytes (M), T-cells (T), CD4^+^ T-cells (T4), CD8^+^ T-cells (T8) and B-cells (B). Additionally, a skin biopsy (S) was collected at age 110, and the subject agreed to post-mortem brain donation, allowing the investigation of brain-cortex (C). Her blood showed no signs of cytopenia, dysplasia, or other clinical signs of hematological malignancies.

# SM2: Clinical assessment of the hematological status

To rule out myeloid dysplasia, we investigated whether the DNA derived from a peripheral blood sample collected at age 110 had accumulated a mutation in one or more of the 54 genes frequently mutated in myeloid neoplasia. For this we used the TruSight Myeloid panel (Illumina v1.4) and re-sequenced on a MiSeq instrument (Illumina) at >500x read depth. Obtained data was analysed using Illumina’s Isis (v2.6.2.3). Assayed genes included: *ABL1, CEBPA, HRAS, MYD88, SF3B1, ASXL1, CSF3R, IDH1, NOTCH1, SMC1A, ATRX, CUX1, IDH2, NPM1, SMC3, BCOR, DNMT3A, IKZF1, NRAS, SRSF2, BCORL1, ETV6/TEL, JAK2, PDGFRA, STAG2, BRAF, EZH2, JAK3, PHF6, TET2, CALR, FBXW7, KDM6A, PTEN, TP53, CBL, FLT3, KIT, PTPN11, U2AF1, CBLB, GATA1, KRAS, RAD21, WT1, CBLC, GATA2, MLL, RUNX1, ZRSR2, CDKN2A, GNAS, MPL, SETBP1.*

Furthermore, the presence of clonal BCR or TCR recombinations in peripheral blood may point to suspect lympho-proliferations, or a recent or sustained antigenic stimulation. The diversity in both T and B-cell receptor repertoire was investigated using IdentiClone™ PCR assays followed by quantification using an ABI Capillary Electrophoresis Instrument.

# SM3: Whole genome sequencing

To detect somatic mutations in W111’s blood, we analyzed DNA derived from peripheral blood and a skin biopsy collected on the same day using Whole Genome Sequencing (WGS) using SOLiD sequencing technology (Life Technologies) at 80x mean depth. Library preparations were performed according to the Fragment Library Preparation 5500 series SOLiD protocol (4460960A) and executed using an automated routine on the Biomek FXp workstation. The resulting library was prepared for sequencing according to SOLiD protocols EZB_Emuls_gsg_4441486E, EZB_AMP_GSG_4443494E, and EZB_enrich_gsg_4443496E. Sequencing was conducted using the SOLiD 5500XL with paired end sequences, 75bp forward en 35bp reverse, according to the 5500 user guide 4456991L protocol. Resulting data was aligned with Lifescope (v2.5), after which variants were called using GATK (v2.7.5), involving the following steps: Local read depths were determined using GATK’s DepthOfCoverage; local realignment around known indels was performed calling GATK’s RealignerTargetCreator and subsequently IndelRealigner. Mate information was then recovered using Picard Tools (v1.105). Base call recalibration was performed by running BaseRecalibrator after which variants were called using HaplotypeCaller. Variants were annotated using KGGSeq (v0.4).

Single Nucleotide Variations (SNVs) were called in both samples using GATK Haplotypecaller^14^ and somatic mutations were identified using *in-house* software Sommix (**Supplemental Methods 4**).

# SM4: Sommix

## Rationale and Design

To detect somatic mutations in blood using skin as a control we had to correct for the contamination of skin cells with blood. For this purpose, we developed Sommix. Input data for Sommix are mapped reads from a blood and a skin sample as well as the expected contamination fraction *c*. The output of the algorithm is the most probable combination of genotypes for the blood and skin samples at every genomic position.

To narrow the search space, we did not assess the whole genome with Sommix, but only the positions where GATK found a possible indication of a variant in either blood or skin. BAM files were realigned and recalibrated, after which the GATK Haplotypecaller (version 3.1) was used to obtain variant calls. A low confidence threshold of 10 for emitting a variant was used, resulting in a total of 4,554,470 possible variant sites. All these sites, irrespective of their call status or quality, were reinvestigated through Sommix.

We denote $\boldsymbol{G}=\{ AA, AC, AG, AT, CC, CG, CT, GG, GT, TT \}$ as the set of all possible genotypes and $G_{b},G_{s} \in\boldsymbol{G}$ as the genotype of the blood and the skin sample respectively in a given genomic position. Sommix identifies the most probable pair of $G_{b},G_{s}$ out of the 100 possible combinations. If there are $N_{b}$ reads covering the specific position in blood and $N_{s}$ reads in skin, the probability of each genotype pair given the available reads (*D*) is given by:

$$P\left( G_{b},G_{s} \right|D)=\frac{P\left( G_{b},G_{s} \right)\cdot P\left( D | G_{b},G_{s} \right)}{P(D)}= \frac{P\left( G_{b},G_{s} \right) \cdot\prod_{i=1}^{N_{b}} P(r_{i}\left| G_{b},G_{s} \right) \cdot\prod_{j=1}^{N_{s}} P(r_{j}\left| G_{b},G_{s} \right)}{P(D)} (1)$$

where $P\left( G_{b},G_{s} \right)$ is the prior probability of the genotype pair $G_{b},G_{s}$ and $P(D)$ is a normalizing factor. Since $P\left( D \right)$ is the same for all 100 genotype combinations, it does not influence the final result. Therefore, Sommix only computes the numerator of Eq. 1.

## Computation of Priors

Let *g*, *s* be the prior probability of observing a germ line and a somatic mutation respectively in any position.

Let R be the reference base and V_1_, V_2_, V_3_ each one of the remaining three bases.

The prior for a genotype pair is

$$P\left( G_{b},G_{s} \right)=P\left( G_{s} \right) P\left( G_{b}|G_{s} \right) (2)$$

where the prior of a germ line mutation is set to:

$$P\left( G_{s} \right)=\left\{ \begin{aligned} 1-g, G_{s}=RR \\ \frac{2g}{3}, G_{s}=RV_{i} \\ \frac{g}{3}, G_{s}=V_{i}V_{j} \end{aligned} \right.(3)$$

For loci in dbSNP (build 141), *g* is set to 0.2, while for locations not in dbSNP, it is set to 0.001.

The prior of a somatic mutation is set to:

$$P\left( G_{b}|G_{s} \right)=\left\{ \begin{aligned} 1-s, G_{b}=G_{s} \\ s, if G_{b} and G_{s} have 1 allele in common \\ 0, otherwise \end{aligned} \right. (4)$$

The prior *s* is set to 1e-07 (corresponding to an expectation of approximately 300 positions in the whole genome with a somatic mutation).

## Somatic genotype fraction estimate

Sommix calculates for every genotype pair $G_{b},G_{s}$ the fraction to which a somatic genotype is present in a position, which we denote as $V\left( G_{b},G_{s} \right)$. This value is the weighted average of the estimate of this fraction in blood and skin. If $G_{b}=G_{s}$, then $V\left( G_{b},G_{s} \right)=0$.

For a heterozygous somatic mutation:

$$V^{blood}\left( G_{b},G_{s} \right)=2\frac{\#reads with somatic allele in blood}{\#reads with reference allele in blood+\#reads with somatic allele in blood} (5)$$

$$V^{skin}\left( G_{b},G_{s} \right)=2\frac{\#reads with somatic allele in skin}{\#reads with reference allele in skin+\#reads with somatic allele in skins} (6)$$

Other more complex somatic events, such as homozygous somatic mutations and/or the presence of germ line mutations are handled similarly.

To obtain comparable somatic genotype fractions, we convert the estimate $V^{skin}\left( G_{b},G_{s} \right)$ to $V^{blood*skin}\left( G_{b},G_{s} \right)$ through use of the expected amount of contamination of blood cells in the skin sample:

$$V^{blood*skin}\left( G_{b},G_{s} \right)=\frac{V^{skin}\left( G_{b},G_{s} \right)}{c} (7)$$

Then the weighted average is calculated as follows:

$$V\left( G_{b},G_{s} \right)=\frac{\#reads in skin\cdot c\cdot V^{blood*skin}\left( G_{b},G_{s} \right)+ \#reads in blood\cdot V^{blood}\left( G_{b},G_{s} \right)}{\#reads in skin\cdot c+\#reads in blood} (8)$$

This reduces the weight of the skin sample by the expected contamination fraction.

## Likelihood of a read given a genotype pair

The likelihood of each base given a genotype depends on whether it is present in the genotype and on whether it is a reference base or not. It also depends on the base-calling quality (*BQ*) and mapping quality (*MQ*) in the position in question for a given read. Let $e_{bc}$ be the probability that the base called in a certain position in a given read is erroneous and $e_{m}$ the probability that a mapping error has occurred in the read.

Base *b* given the genotype *G* = *bb* can be observed in the following ways:

- The sequenced base is *b* and the base-calling and mapping are done correctly.
- The sequenced base is any of the 4 bases and a base-calling error has occurred.
- The sequenced base is *b*, the correct base has been called, but a mapping error has occurred. In that case the likelihood to observe a certain *b* depends on the reference sequence, as an incorrect mapping can only occur for reads that are very similar to the reference sequence at the mapped location. We assume, therefore, that an incorrectly mapped read at each position follows the reference with 0.95 probability, and conversely, includes a non-reference base at 0.05 probability.

If *G* is heterozygous for *b* (*G* = *bx*), then the ways to observe base *b* are similar, with one difference: In the case where both the base-calling and mapping are correct, there is still 0.5 probability of observing each of the two bases.

Finally, if *G* does not contain base *b*, then observing *b* can be either to a base calling or a mapping error. In the case of correct base calling and a mapping error, we still expect a higher chance of observing base *b*, if it is the reference base.

The above is summed up in Eq. 9, where *β* = 0.95 if *b* is the reference base and 0.05 if not.

$$P\left( b | G \right)= \left\{ \begin{aligned} \frac{e_{bc}}{4}+\left( 1-e_{bc} \right)\left( 1-e_{m}+\beta e_{m} \right), G=bb \\ \frac{e_{bc}}{4}+\left( 1-e_{bc} \right)\left( \frac{1-e_{m}}{2}+\beta e_{m} \right), G=bx \\ \frac{e_{bc}}{4}+\left( 1-e_{bc} \right)\beta e_{m}, b \notin G \end{aligned} \right. (9)$$

The probability of observing base $b^{s}$ in skin is:

$$P({r_{i}=b}^{s}\left| G_{b},G_{s} \right)=\left( 1-V\left( G_{b},G_{s} \right)\cdot c \right)\cdot P\left( b^{s} | G_{s} \right)+V\left( G_{b},G_{s} \right)\cdot c\cdot P\left( b^{s} | G_{b} \right) (10)$$

The probability of observing base $b^{b}$ in blood is:

$$P({r_{i}=b}^{b}\left| G_{b},G_{s} \right)=\left( 1-V\left( G_{b},G_{s} \right) \right)\cdot P\left( b^{b} | G_{s} \right)+V\left( G_{b},G_{s} \right) \cdot P\left( b^{b} | G_{b} \right) (11)$$

## Heterozygosity Correction

In case of germ line heterozygosity, the expected allele frequency is 0.5 for both alleles. However, in practice, amplification biases and mapping affinities frequently lead to quite different allele frequencies. Not including this in the scoring framework would lead to false positive calls, as germ line heterozygous loci with deviating allele frequency patterns could also be interpreted as a mixture of a germ line and a somatic genotype.

To correct for this, we also estimate the heterozygosity allele frequency for germ line variants directly based on the reads.

If no somatic event occurred, then the expected germ line heterozygous VAF in skin is the average of the calculated VAFs in blood and skin weighted by the number of reads in each sample.

In case of a somatic event, we subtract the part of the reads that can be attributed to the somatic genotype fraction, using the following correction factors:

$${cor}_{b}=1-\frac{V\left( G_{b},G_{s} \right)}{1+V\left( G_{b},G_{s} \right)} (12)$$

$${cor}_{s}=1-\frac{V\left( G_{b},G_{s} \right)\cdot c}{1+V\left( G_{b},G_{s} \right)\cdot c} (13)$$

If the somatic genotype is homozygous (and the germ line heterozygous), the estimated heterozygous allele frequency is now estimated as:

${VAF}_{corrected}=\frac{{cor}_{s}\cdot\#reads with som. allele in skin+ {cor}_{b}\cdot\#reads with som. allele in skin}{{cor}_{s}\cdot\#reads with som. allele in skin+{cor}_{b}\cdot\#reads with som. allele in blood+\#reads with ref. allele in skin+\#reads with ref. allele in blood} (14)$

More complex cases that involve more than one somatic mutation are handled similarly.

Based on these corrections, in the cases when the germ line genotype is heterozygous, the likelihood of each of the two bases given the genotype needs to be revisited. More specifically, we assumed earlier that in a heterozygous site without base-calling and mapping errors there is 0.5 chance that each of the bases might be observed. We showed here that this is not the case, so this likelihood equation for the germ line was modified as follows:

$$\left( b | G \right)=\frac{e_{bc}}{4}+\left( 1-e_{bc} \right)\left( (1-e_{m})\cdot{VAF}_{corrected}+\beta e_{m} \right), G=bx in germline (15)$$

## Mappability correction

To identify locations that might have high mismapping ratios, we downloaded the 50bp mappability track from UCSC (Raney et al. 2011). The mappability for each locus is encoded as 1.0 divided by the number of 50bp genome sections that, with up to two mismatches, can map to this locus. We include this in the mapping error probability calculations, by adapting this probability as follows:

$$e_{m}=\max\left( e_{m}, 1.0- mappability \right)(16)$$

## Depth correction

Alternatively, an anomalously high read depth can also identify mismapped reads. This might be an indication of repeat regions that are mapped to the same locus on the reference genome. Given an expected read depth of 80x, we calculated a mapping quality based on depth as follows:

$$e_{depth}=1-\frac{depth \cdot2.0\cdot1.5}{depth in skin+depth in blood} (17)$$

Similarly, as for the mappability correction, we included this in the mapping quality:

$$e_{m}=\max\left( e_{m}, 1.0- mappability \right) (18)$$

## Sommix Scores

The optimal genotypes pair is the one that maximizes $log(P\left( G_{b},G_{s} \right|D))$. The Sommix score is calculated as the logarithm of the likelihood ratio between the two most probable genotypes. This score is 0 when two genotypes pairs are equally likely and infinity when only one genotypes pair has non-zero likelihood (e.g. if there are only perfect reads for the base A in both blood and skin in a certain locus, only the pair AA-AA would have non-zero likelihood). Loci with a homozygous reference genotype in skin and a heterozygous genotype in blood that had a positive Sommix score were selected for further analysis.

## Additional filtering

To obtain a high quality set of calls, three additional filtering steps were performed:

*Strand Bias*

To identify positions with high strand bias, we used the hypergeometric test to test whether strand bias was present. Every cell of the contingency table contains the number of reads that supports the corresponding allele and map to the corresponding strand in both blood and skin. We used a conservative threshold, were loci with a p-value lower than 0.1 were excluded.

*Tri-allelic variants*

Conservatively, loci with 3 alleles were not considered, as these are more likely to constitute mismapping reads rather than a true complex somatic event.

*Concordance with GATK’s called variant types*

Putative blood mutations were excluded from further analysis in positions where Sommix detected a somatic single nucleotide substitution (SNV) whereas GATK detected a somatic indel.

## Division into Confidence Tiers

Application of Sommix yielded 929 bi-allelic somatic single nucleotide mutations in blood. Subsequent filtering removed 262 with a strand bias, and 17 with a discordant variant type called by GATK. The remaining 650 putative single nucleotide somatic mutations were classified into three nearly equal-sized levels of confidence: Tier 1 Sommix score > 1.7; Tier 2 Sommix score > 0.65; Tier 3 Sommix score > 0.

# SM5: Amplicon re-sequencing

Amplicon re-sequencing was performed to 1] validate the putative somatic mutations identified by whole genome SOLiD sequencing in peripheral blood and skin 2] to assess and compare the VAFs of validated somatic SNVs in peripheral blood at age 103, 110 and 111 3] to assess and compare the VAFs of validated somatic SNVs in sorted immune subsets. Ion Torrent amplicon re-sequencing was performed at 6000x average read-depth (Proton Ampliseq sequencing, Thermo Fisher Scientific, **Table ST2**). Variants were called using Torrent Variant Caller (TVC) version 5.0.0 (Life Technologies).

For this purpose, amplicon design software (Ion AmpliSeq Designer V4.2) was employed to automatically design 100bp insert size amplicons covering the putative somatic variants. AmpliSeq libraries were prepared according to the Ion AmpliSeq™ Library Preparation protocol (MAN0006735, Revision A.0). Sequencing was performed according to Ion PI™ IC 200 Kit protocol (MAN0010078, Revision B.0). Obtained data was analyzed with Torrent Suite (V4.4.2) to verify whether the targeted genomic areas were sufficiently covered, using the following parameters:

**BeadFind Args:** justBeadFind –beadfind-minlivesnr 3 –region-size=216,224

–total-timeout 600

**Analysis Args:** Analysis –from-beadfind –clonal-filter-bkgmodel true

–region-size=216,224 –bkg-bfmask-update false

–gpuWorkLoad 1 –total-timeout 600

–gopt /opt/ion/config/goptp1.1.17ampliseqexome.param.json

**Pre-BaseCaller Args for calibration:** BaseCaller –barcode-filter 0.01

–barcode-filter-minreads 10 –keypass-filter on

–phasing-residual-filter=2.0 –num-unfiltered 1000

–max-phasing-levels 2

**Calibration Args:** calibrate –skipDroop

**BaseCaller Args:** BaseCaller –barcode-filter 0.01

–barcode-filter-minreads 10 –keypass-filter on

–phasing-residual-filter=2.0 –num-unfiltered 1000

–barcode-filter-postpone 1

**Alignment Args:** stage1 map4

**IonStats Args:**

**Analysis Parameters:** default

# SM6: Identification of candidate driver mutations

WGS data was also analyzed for the presence of candidate driver mutations in 16 genes previously reported to be recurrently mutated in the blood of apparently healthy elderly individuals: *DNMT3A*, *TET2*, *ASXL1*, *TP53*, *JAK2*, *SF3B1*, *GNB1*, *CBL*, *SRSF2*, *GNAS*, *BRCC3*, *CREBBP*, *NRAS*, *RAD21*, *U2AF1*, *PPM1D*^7-11^. For this purpose, SNPs and short indels called by GATK Haplotypecaller were consecutively filtered according to the following five criteria. *i*) Positioned within or near, max 5 nucleotides, of the coding sequence of a transcript annotated to the aforementioned genes (hg19, UCSC Refgene definitions, UCSC’s Variant Annotation Integrator (<https://genome.ucsc.edu/cgi-bin/hgVai>). *ii*) A minimal read depth of the mutant allele of 3 and 6 for respectively SNVs and indels. *iii*) A protein altering predicted impact (UCSC Variant Annotation Integrator, NCBI Refseq curated subset). *iv*) Adhering to the gene specific mutational profiles, as previously compiled^5^(**Table ST3**). *v*) Called in blood, but not in brain. A *DNMT3A* splice mutation was identified and confirmed by re-sequencing the peripheral sample blood drawn at 110 years and 3 months using the Illumina TruSight Myeloid panel and MiSeq instrument at 961x and visualized in Integrative Genome Viewer (IGV, Broad Institute, version 2.4.9).

# SM7: Mutational Signatures

Analyses of their tri-nucleotide context and resemblance to previously published and curated mutational signatures (COSMIC, V2 - March 2015) was performed with the R package MutationalPatterns^15^.

# SM8: Inferring the subclonal architecture

Somatic mutations were clustered by their Variant Allele Frequencies (VAFs) using SciClone version 1.1.0^16^ (**Table ST2**). Resulting cluster annotations and VAFs were subsequently analyzed with SCHISM version 1.1.2^17^, to infer a phylogenetic tree describing the most probable order in which subclonal expansion events (clusters of SNVs) occurred. The percentage of cells carrying a set of mutations marking a specific subclonal expansion event was computed using the median VAF across all assigned mutations. To obtain the percentage of cells that belong to a particular subclone, the median VAF across the mutations marking a specific expansion event were corrected for the median VAF across the mutations marking the immediate child subclonal expansion event(s) in the tree, e.g. A’ = A – B.

# SM9: Immuno-phenotyping by flow cytrometry

PBMCs were incubated with a titrated cocktail of antibodies for 30 minutes on ice in the dark. Cells were subsequently washed with FACS buffer (PBS supplemented with 2% FCS and Na-azide) prior to flow cytometry analysis using FACS Fortessa-X20 (BD Biosciences). The employed antibody mix consisted of: FITC-anti-CD57 (clone HCD57); PE-anti-CD28 (clone CD28.2); APC-Cy7-anti-CD4 (clone RPA-T4); PE-Cy7-anti-CD197 (CCR7, clone 3D12); Pacific Blue-anti-CD27 (clone M-T271), BV650-anti-CD8 (clone SK1), all were from BD Pharmingen (USA). Alexa Fluor 700-anti-CD45RA (clone HI100); BV605-anti-CD31 (clone WM59) were from Biolegend (San Diego, USA). Phenotypic data were analyzed using FlowJo software (TreeStar).

# SM10: T-cell Receptor Excision Circle (TREC) assays

T-cell receptor excision circle (TREC) assays are based on the rearrangement of the T-cell receptor occurring early during T-cell development^6^. This rearrangement results in the formation of a coding joint (CJ) which remains stably present in the genomic DNA, and a signal joint (SJ) on the corresponding excision circle. As with every cell division, SJ is diluted, while CJ is stably maintained in the genomic DNA, it is possible to derive the number of divisions the assayed cells have undergone from the difference between CJ and SJ^18^. TRECs analyses were essentially performed as previously described^19^. In short, DNA was extracted from peripheral blood or sorted T-cell subsets, after which two parallel real-time PCRs are performed that quantify the signal joint (SJ) or the coding joint (Cj) relative to an internal control of Albumin.

# SM11: Mixed Leukocyte Reactions (MLR)

Antigen-dependent T-cell proliferation capacity was determined using a mixed leukocyte reaction (MLR). PBMC’s from test samples were cultured with irradiated HLA-mismatched PBMCs (3000 rad). To assess the general capacity of T-cells to proliferate, PBMCs were stimulated with Interleukin-2 (IL-2, 25 IU/ml). Cells were incubated in triplo in the 96-well round bottom plates in IMDM medium conditioned with Glutamine, Pen/Strep and 10% inactivated Human Serum (HS, Sanquin, Amsterdam) for 5 days. Radioactive thymidine (2 µCi/ml) was added in the last 18 hrs of the culture to enable incorporation in the DNA of proliferating cells and the radioactivity in DNA recovered from the cells was measured using a scintillation beta-counter. Obtained data (counts per minute, CPM) were used to calculate stimulation index by dividing the CPM values of the test samples by the CPM of test PBMC cultured in medium alone.

Supplementary Results - SR

# SR1: Cataloguing somatic mutations in peripheral blood

To identify somatic mutations that had accumulated in the hematopoietic stem cell compartment of W111, we compared WGS of DNA derived from the peripheral blood collected at age 110 years and 3 months (PB1) with WGS of DNA derived from the skin biopsy collected on the same day (**Supplementary Methods 1**). We identified 650 putative single nucleotide somatic variations (SNVs) (**Figure SF2A, Table ST1**). Concurrent with previous work on the non-leukemic accumulation of somatic mutations in peripheral blood^1^ or in cultures of single expanded hematopoietic stem cells^2-4^, the vast majority of these mutations were non-coding (**Figure SF2B**), did not exhibit any positional preferences in the genome (**Figure SF2**), and were dominated by C>T and T>C transitions (**Figure SF2D**). Moreover, the tri-nucleotide sequence context of the identified mutations (**Figure SF2E, Figure SF4**) largely overlapped with established ‘clock-like’ mutational signatures that represent relatively benign ageing processes^4,5^(**Figure SF4**).

Next, we screened the 650 identified SNVs for potential candidate driver mutations using the mutation definitions compiled by Jaiswal *et al.*^6^ (definitions in **Table ST2**) for 16 genes frequently mutated in the blood of healthy elderly individuals^7-11^. We identified a splice-donor site mutation in intron 11 of DNA (cytosine-5)-methyltransferase 3A (*DNMT3A,* NM_022552.4, chr2:25,469,028 C>T, c.1429+1 G>A). Targeted re-sequencing (**Supplementary Methods 2**) confirmed this variant at 961x read depth, with an estimated variant allele frequency (VAF) of 0.378 (**Figure SF2C**). This suggests that 75.6% of the peripheral blood cells are derived from a single clone carrying a *DNMT3A* splice-donor mutation.

Additionally, of the 650 somatic mutations, one additional mutation modulated protein function: a missense mutation in the *PTPRD* gene (geneID: ENSG00000153707): chr9:8517886:G>A. We confirmed this mutation with amplicon sequencing, and with a VAF of 0.35, it mapped to founding clone A. The mutation was predicted as “probably damaging” by SIFT/Polyphen, and had a CADD score of 24.3 (CADD GRCh37-v1.6)^20^.This gene is considered a tumor suppressor gene since its protein product inhibits cell growth by dephosphorylating the STAT3 protein^21^. This gene is mutated, epigenetically silenced or deleted in several cancer types^21^. However, this gene was not listed as one of the genes that includes potential driver mutations for ARCH/CHIP, according to the definitions compiled by Jaiswal *et al.*^6^

**SR2: Design of an amplicon assay for clonal tracing**

To obtain a set of somatic mutations that can serve as genetic markers for clonal tracing, we performed validation experiments for the 650 identified putative SNVs (**Figure SF3F**). Custom target re-sequencing panels were successfully designed for 474 out of 650 (72.9%) identified somatic mutations. Using brain-cortex as a control tissue, we confirm the somatic origin of 20.1% of the mutations with the lowest evidence for being somatic (Tier 3), 61.6 % of the mutation with intermediate certainty Tier 2), and 97.8% of the mutations with the highest evidence of being somatic (Tier 1) (**Figure SF3F**). Overall, we were able to confirm 307 (64.8%) mutations to be of somatic origin.

Amplicon sequencing in peripheral blood samples collected at ages 103 and 111 revealed that all 307 mutations identified at age 110 years were detectable (**Figure 1A – main paper**). While VAFs generally increased between ages 103 and 110 years, VAFs remained equal or decreased between ages 110 and 111 years. Nevertheless, VAFs were highly inter-correlated between timepoints (Pearson’s r_103->110_ = 0.983 and r_110->111_ = 0.988, **Figure SF6**), and VAF density distributions looked highly similar across time points.

Supplementary Figures - SF

# SF1: Study design


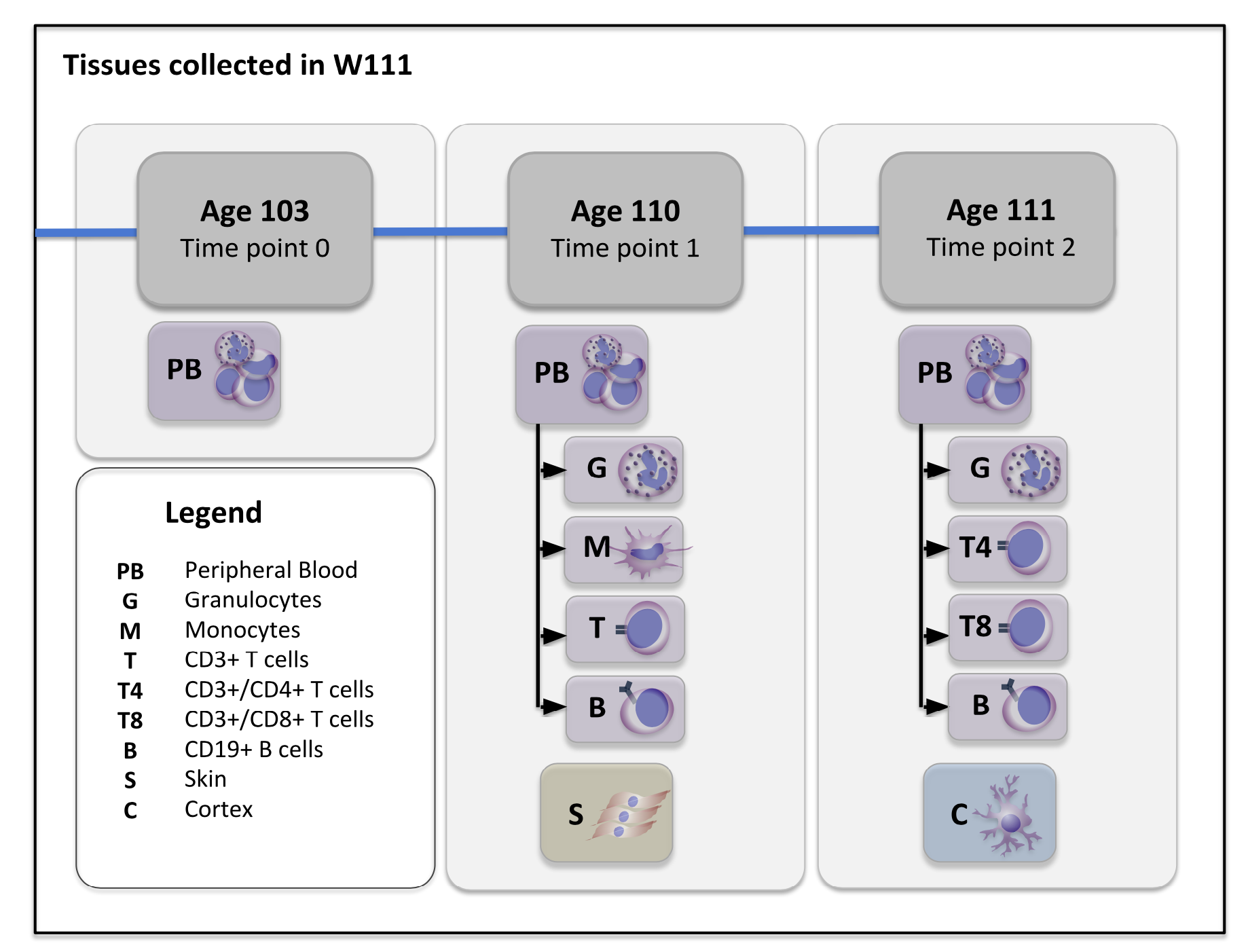


**Figure SF1:** An overview of the tissues collected at three different time points.

# SF2: Cataloguing somatic mutations in peripheral blood


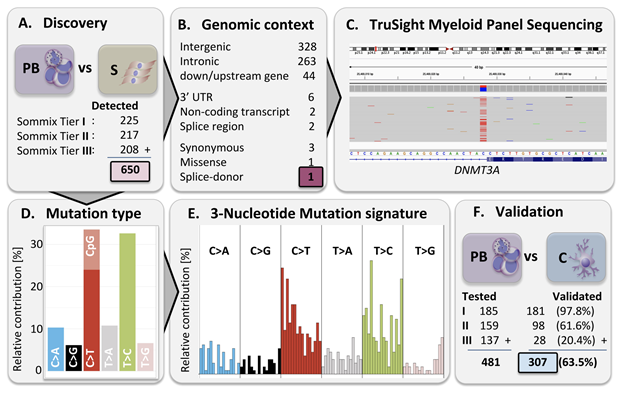
**Figure SF2:** [**A**] Whole genome sequencing of peripheral blood (PB) versus skin (S) and subsequent analysis with Sommix (**Supplementary Methods 4**) identified 650 putative somatic mutations assigned to three confidence tiers (Tier I most confident). [**B**] Identified mutations mostly reside in non-coding genomic locations (UCSC’s Variant Annotation Integrator, hg19, Refgene definitions) [**C**] IGV plot of the validated splice-donor site mutation in *DNMT3A* (NM_022552.4, chr2:25,469,028 C>T, c.1429+1 G>A), altering the G nucleotide of the highly conserved GT intronic sequence^22^. Amplicon sequencing performed at 961x and indicated a VAF of 37.8% for the mutant allele. [**D**] Identified mutations stratified to nucleotide changes show a high frequency of C>T and T>C changes. Part of the C>T nucleotide changes coincide with a CpG site, potentially also affecting DNA methylation. [**E**] 3-Nucleotide sequence context of the identified mutations exhibits a high resemblance to the clock-like mutational signatures 1 and 5 (**Figure SF4**). [**F**] Validation of the identified somatic mutations using amplicon sequencing in peripheral blood (PB) versus Cortex (C), split per confidence tier.

# SF3: Rainfall plot of 650 putative SNVs


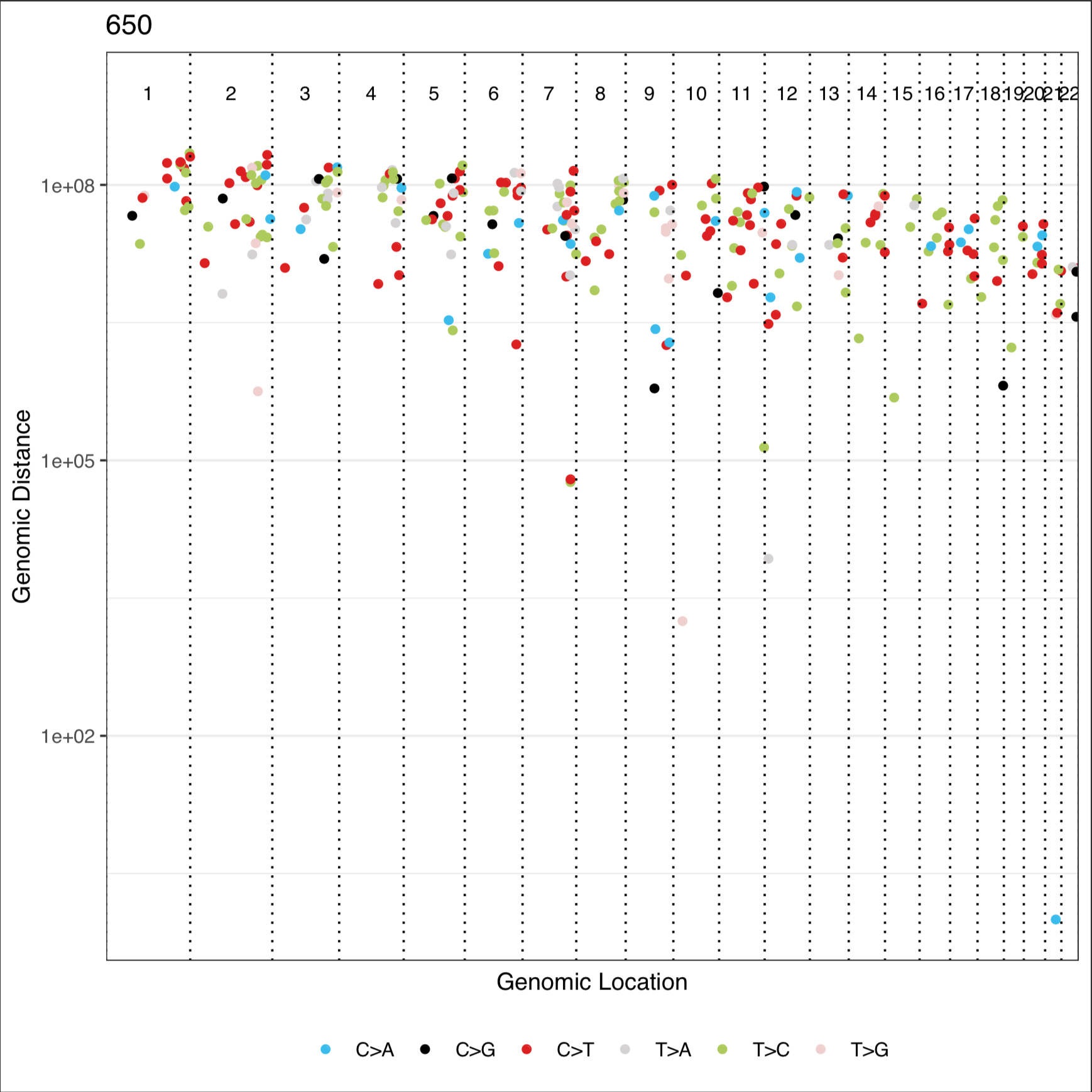


**Figure SF3:** Rainfall plot of the 650 putative somatic SNVs as produced by the package MutationalPatterns^15^. A rainfall plot visualizes the types of mutations (colors), their genomic positioning (x-axis), and the distance between them (y-axis, log distance). Mutation hotspots would appear as clusters of mutations with lower intermutation distance.

# SF4: Tri-nucleotide context of 650 putative SNVs


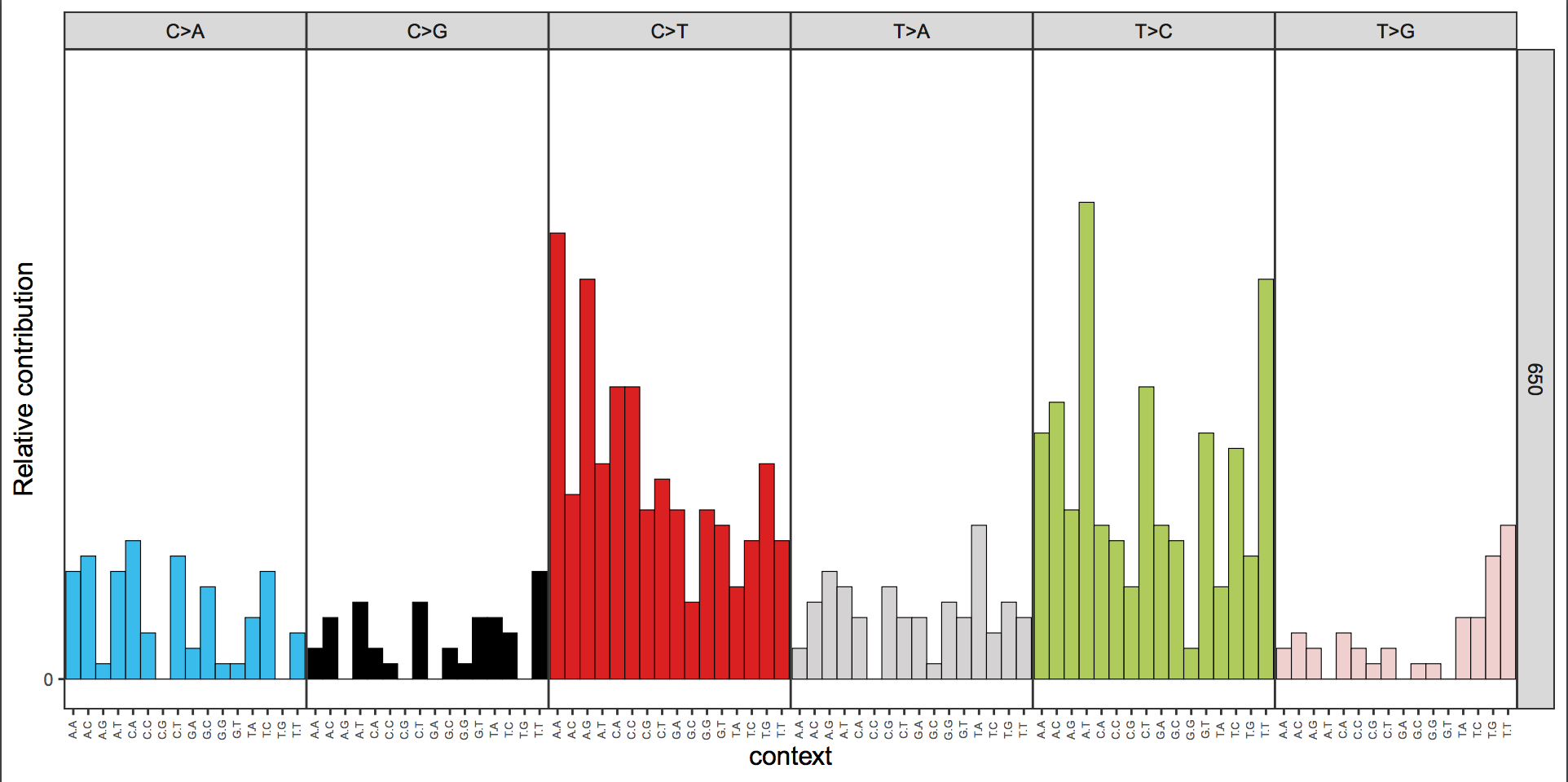


**Figure SF4**: Relative contribution of each of the 96 possible trinucleotide combinations to the 650 putative SNVs as produced by the package MutationalPatterns^15^. Each category of single nucleotide substitutions (indicated at top) is further divided into 16 groups according to the neighboring nucleotides (indicated at bottom). Resulting trinucleotide patterns are typically produced in cancer studies and have been associated with particular oncogenic processes^23,24^. Interestingly, some of these trinucleotide patterns discovered in a pan-cancer setting have been associated with the age of diagnosis, and seem to represent ageing processes^4,5^.

# SF5: Mutational signatures contributing to 650 putative SNVs


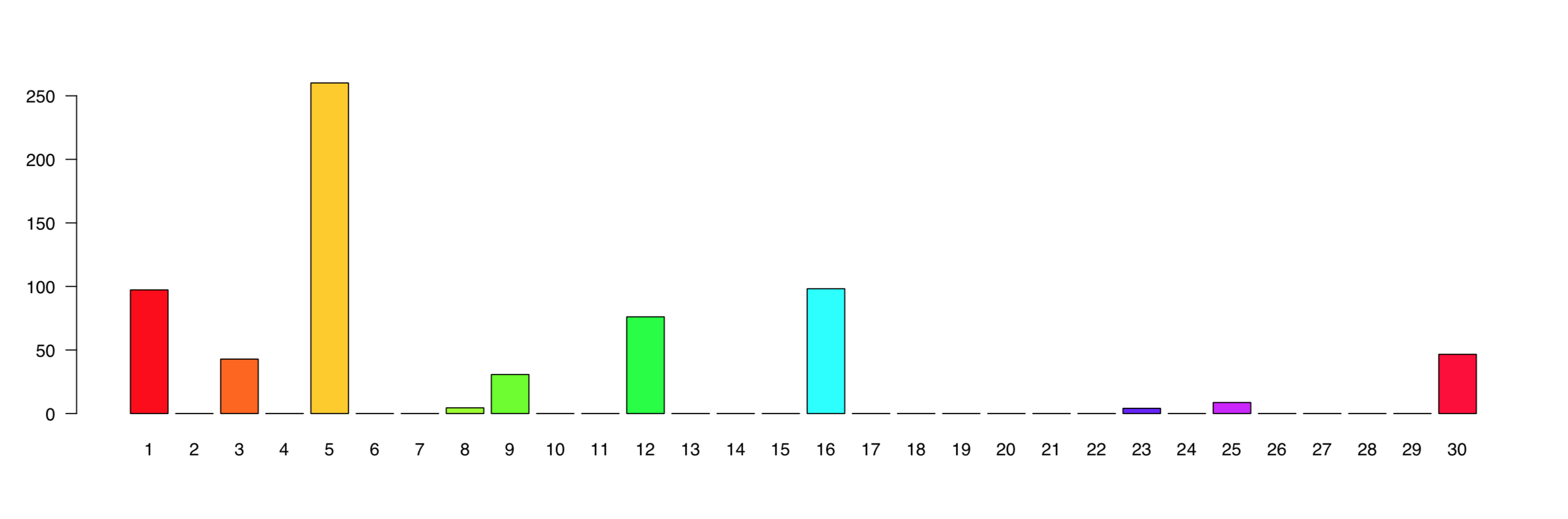


**Figure SF5:**  The number of mutations predicted to be derived from each of the 30 mutational signatures (COSMIC V2 – March 2015). Predictions are based on a linear combination of mutational signatures that most closely reconstructs the mutational pattern observed in this study (**Figure SF3**), as computed by the package MutationalPatterns^15^. Next to signatures 1 and 5, considered to be ageing signatures, also signatures 12 and 16 seem to explain a lot of the observed mutational pattern. Both these signatures are typically observed in liver cancer, however, and typically exhibit a strong transcriptional strand bias. A closer inspection of our mutational profile did not reveal such a transcriptional strand bias (data not shown), hence associations with signatures 12 and 16 were discarded.

# SF6: Pairwise scatter plots of 307 SNVs at PB0, PB1 & PB2


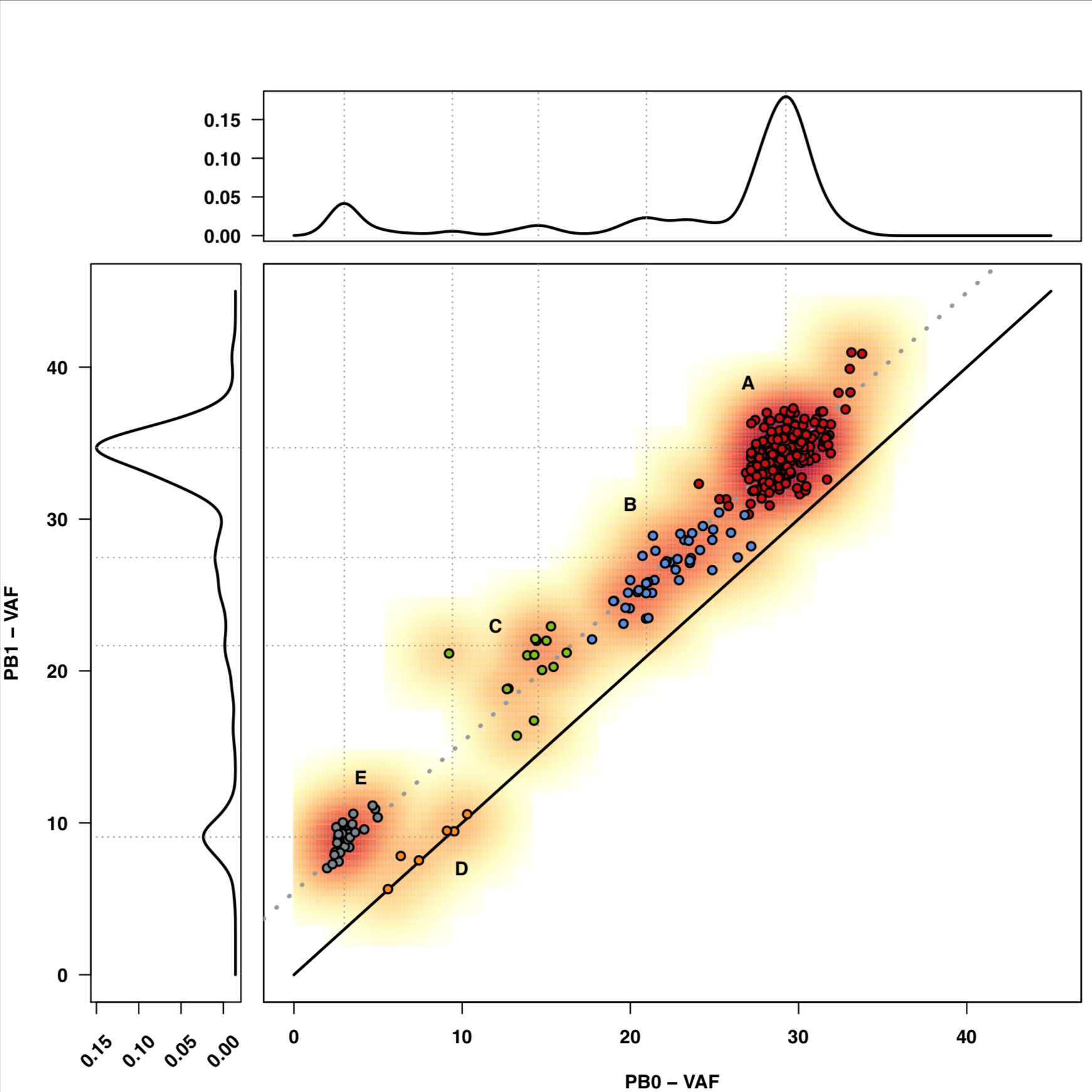


**Figure SF6A:** Pairwise scatter plot of Variant Allele Frequencies (VAF) in peripheral blood measured at age 103 (PB0) and 110 (PB1). Each dot indicates a SNV, and is colored according to subclonal event (A-E). Shades of red represent the local density. Density distributions at the sides represent the density distributions of VAFs at age 110 (y-axis; PB1) and age 103 (x-axis; PB0) respectively.

#
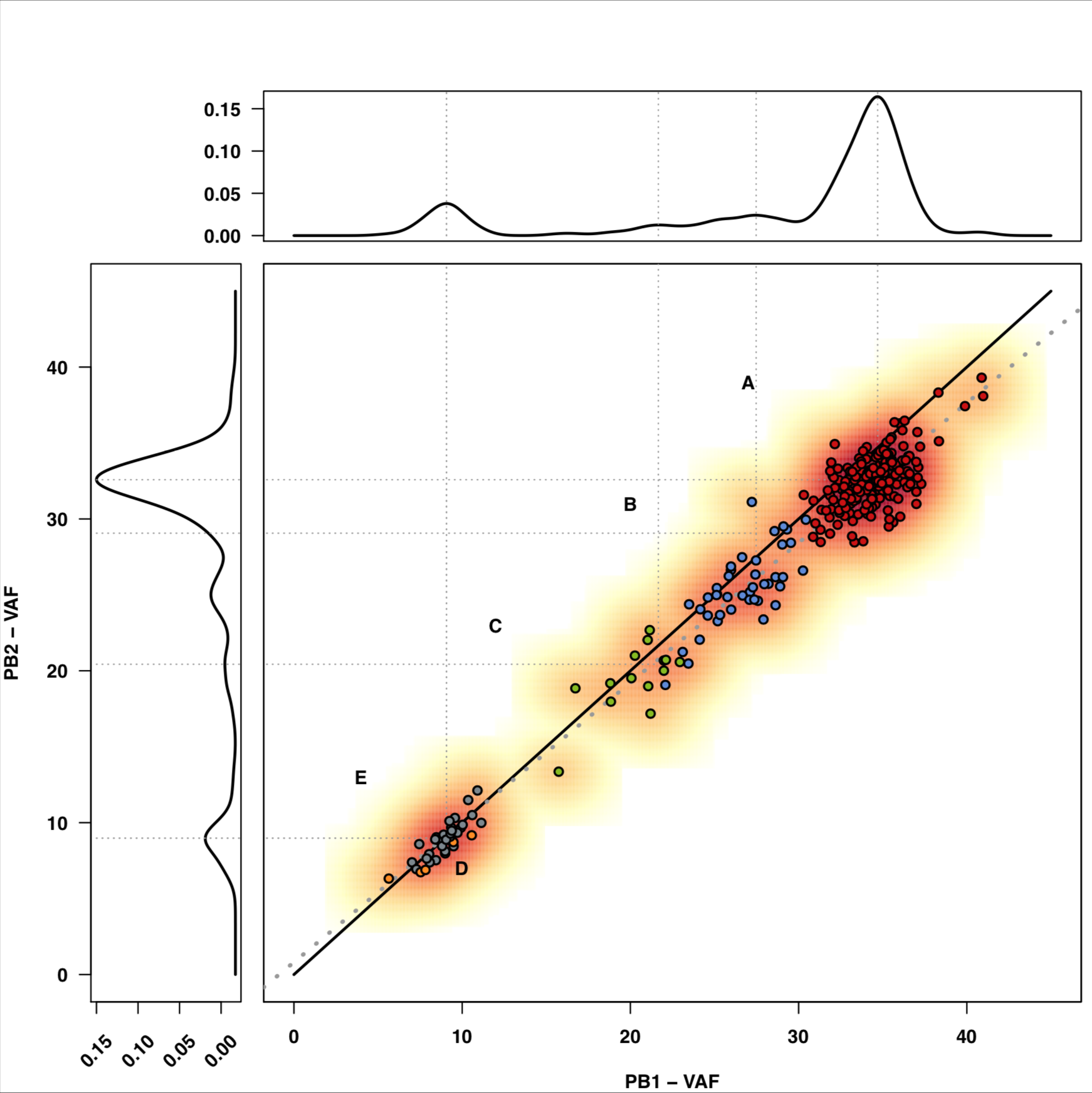


**Figure SF6B:** Pairwise scatter plot of Variant Allele Frequencies (VAF) in peripheral blood measured at age 110 (PB1) and 111 (PB2). Each dot indicates a SNV, and is colored according to subclonal event (A-E). Shades of red represent the local density. Density distributions at the sides represent the density distributions of VAFs at age 110 (y-axis; PB1) and age 111 (x-axis; PB2) respectively.

#
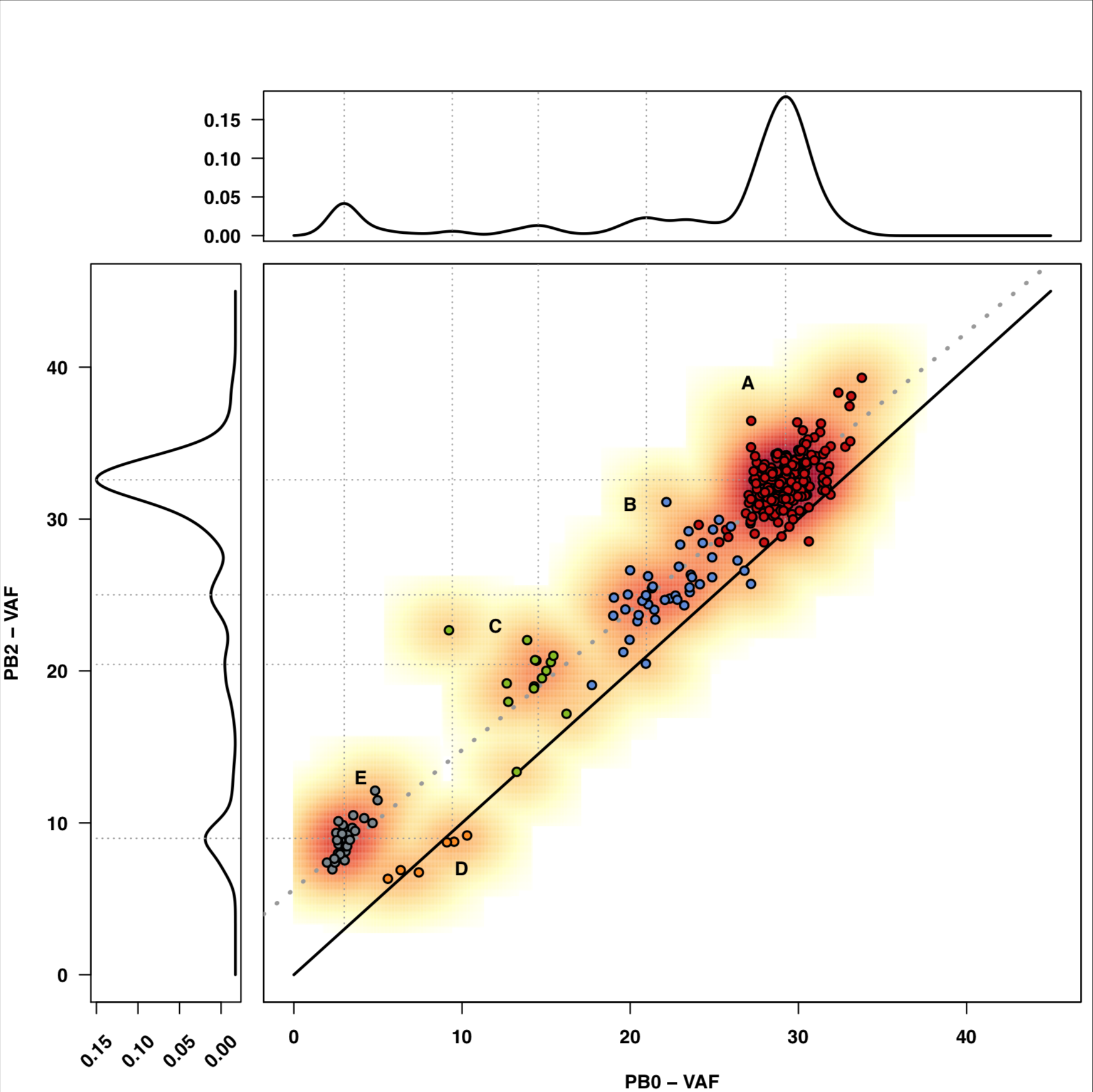


**Figure SF6C:** Pairwise scatter plot of Variant Allele Frequencies (VAF) in peripheral blood measured at age 103 (PB0) and 111 (PB2). Each dot indicates a SNV, and is colored according to subclonal event (A-E). Shades of red represent the local density. Density distributions at the sides represent the density distributions of VAFs at age 103 (y-axis; PB0) and age 111 (x-axis; PB2) respectively.

# SF7: Testing for suspect clonal BCR/TCR gene recombinations


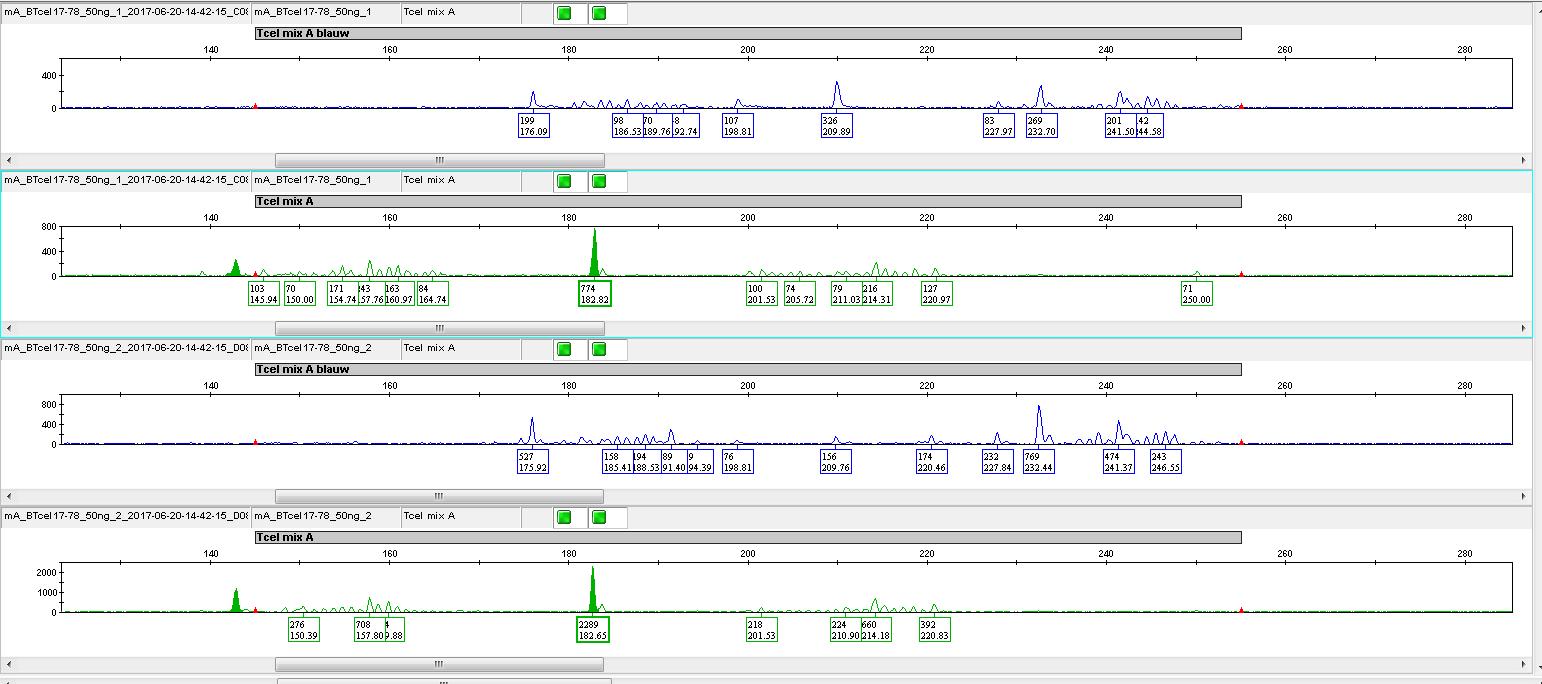


**Figure SF7A**: **T-cell mix A**


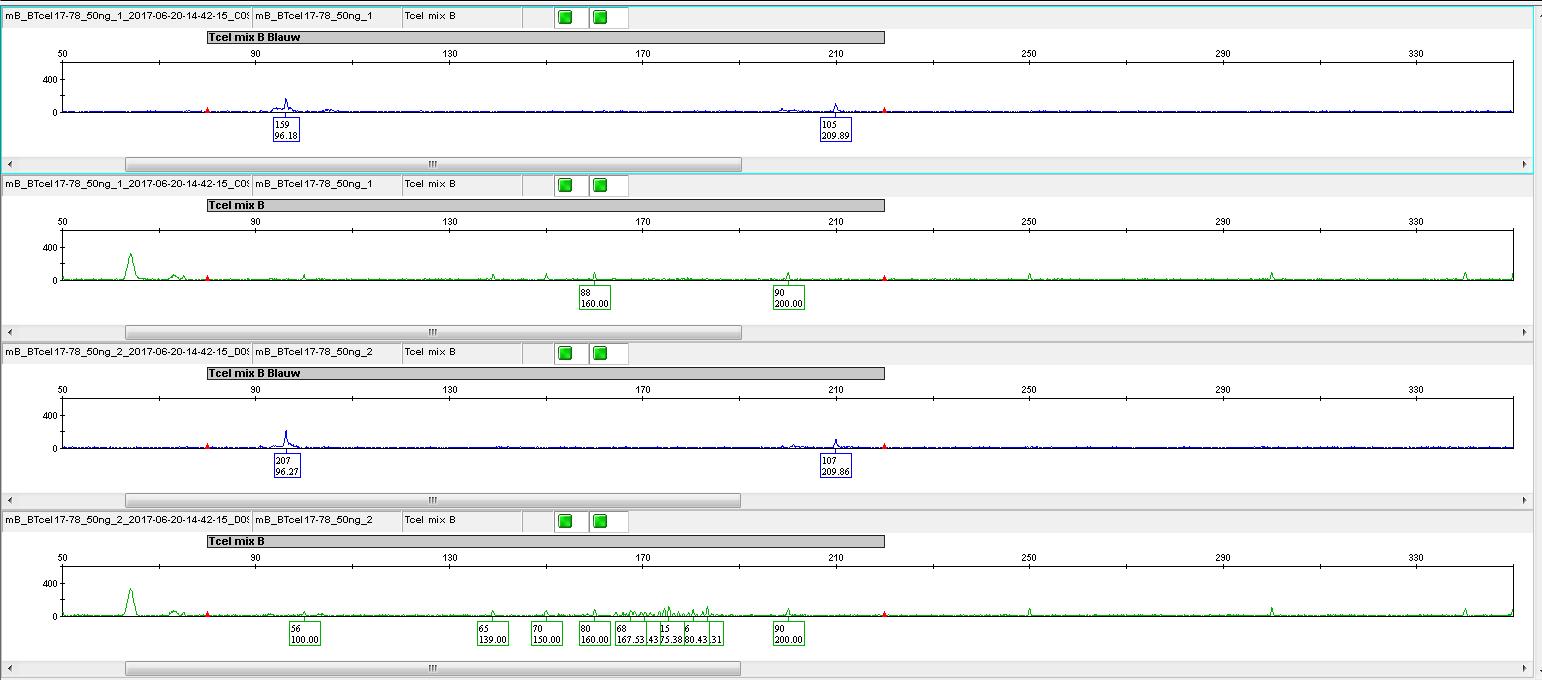


**Figure SF7B**: **T-cell mix B**


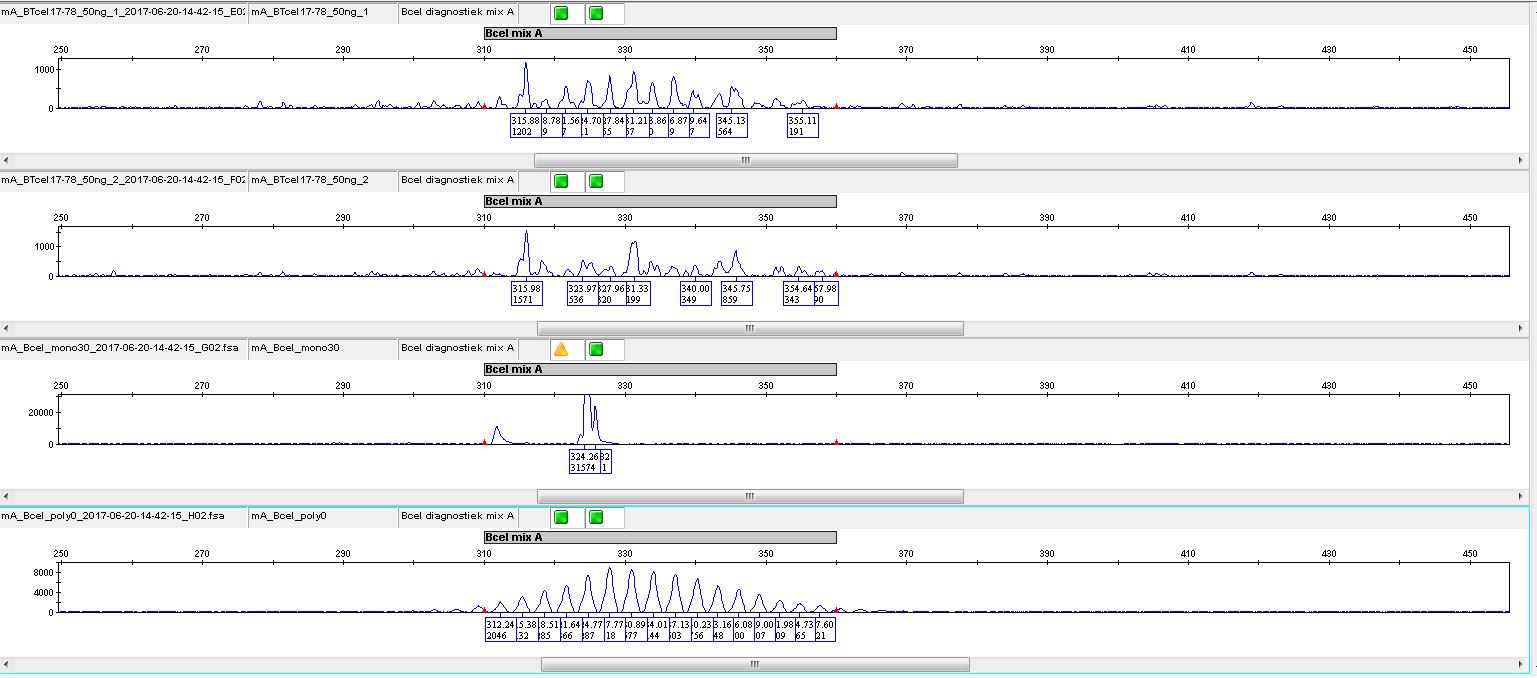


**Figure SF7C**: **B-cell mix A**


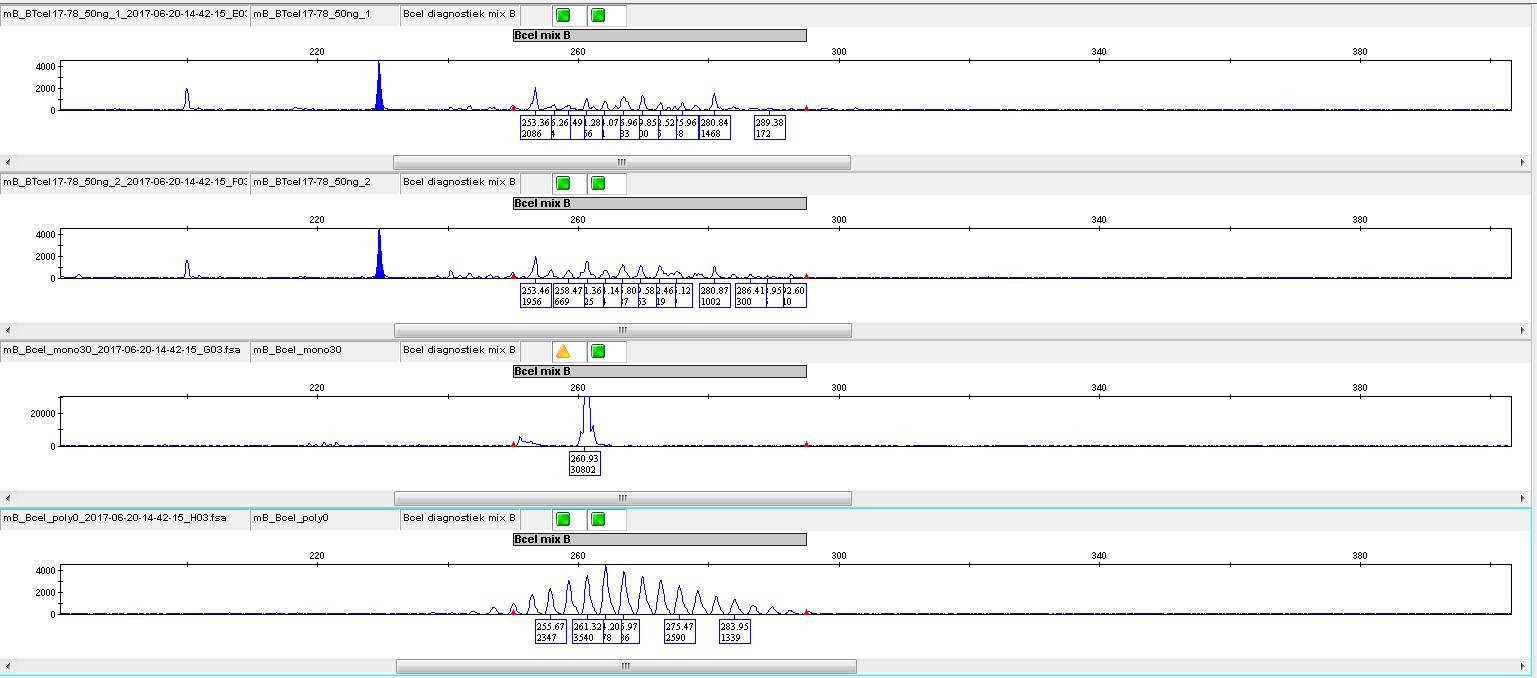


**Figure SF7D**: **B-cell mix B**


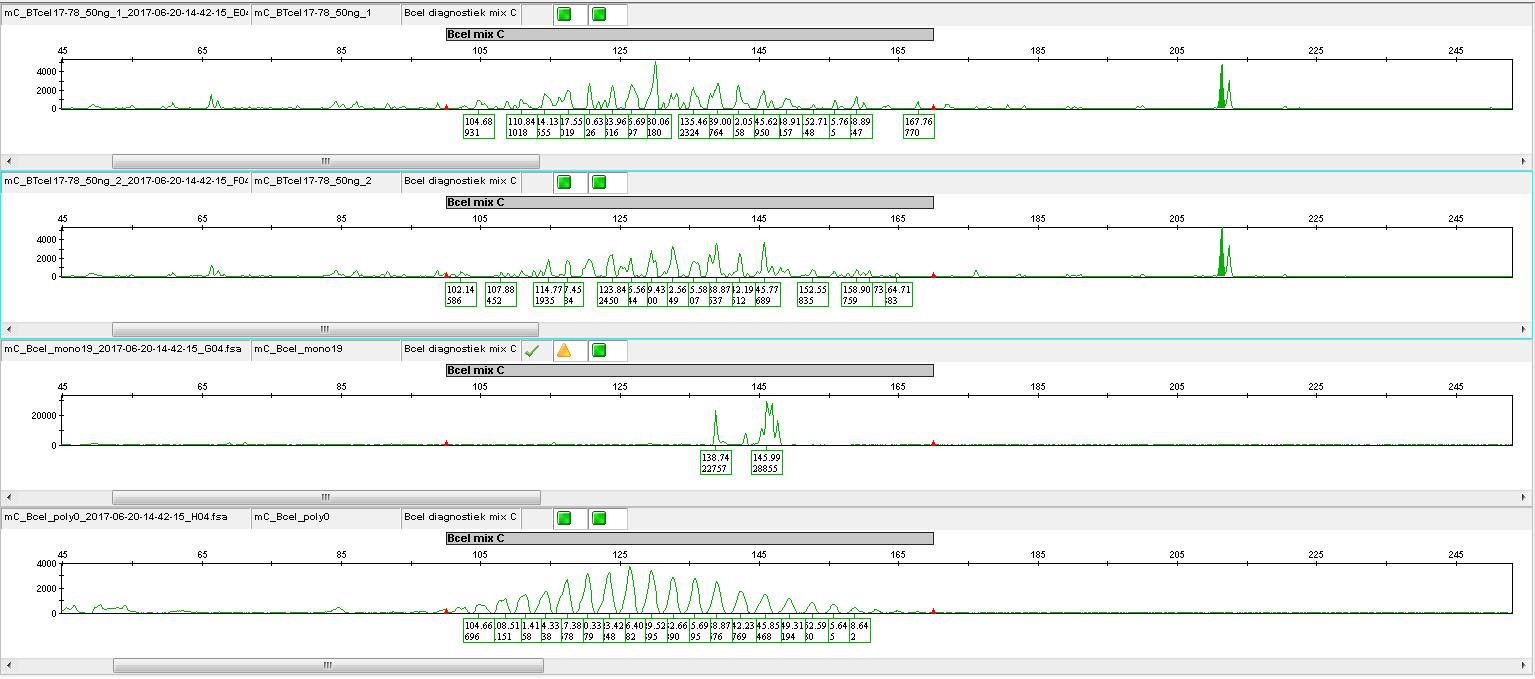


**Figure SF7E**: **B-cell mix C**


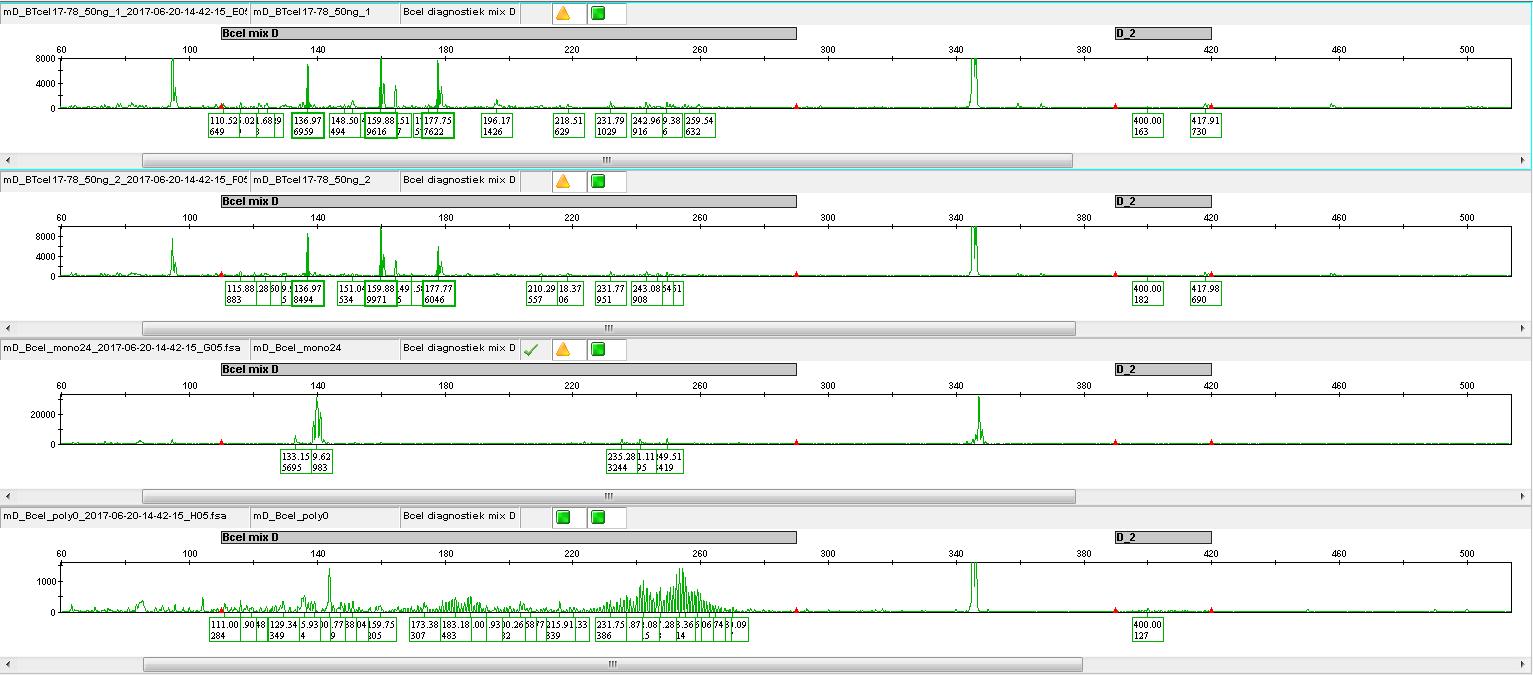


**Figure SF7F**: **B-cell mix D**


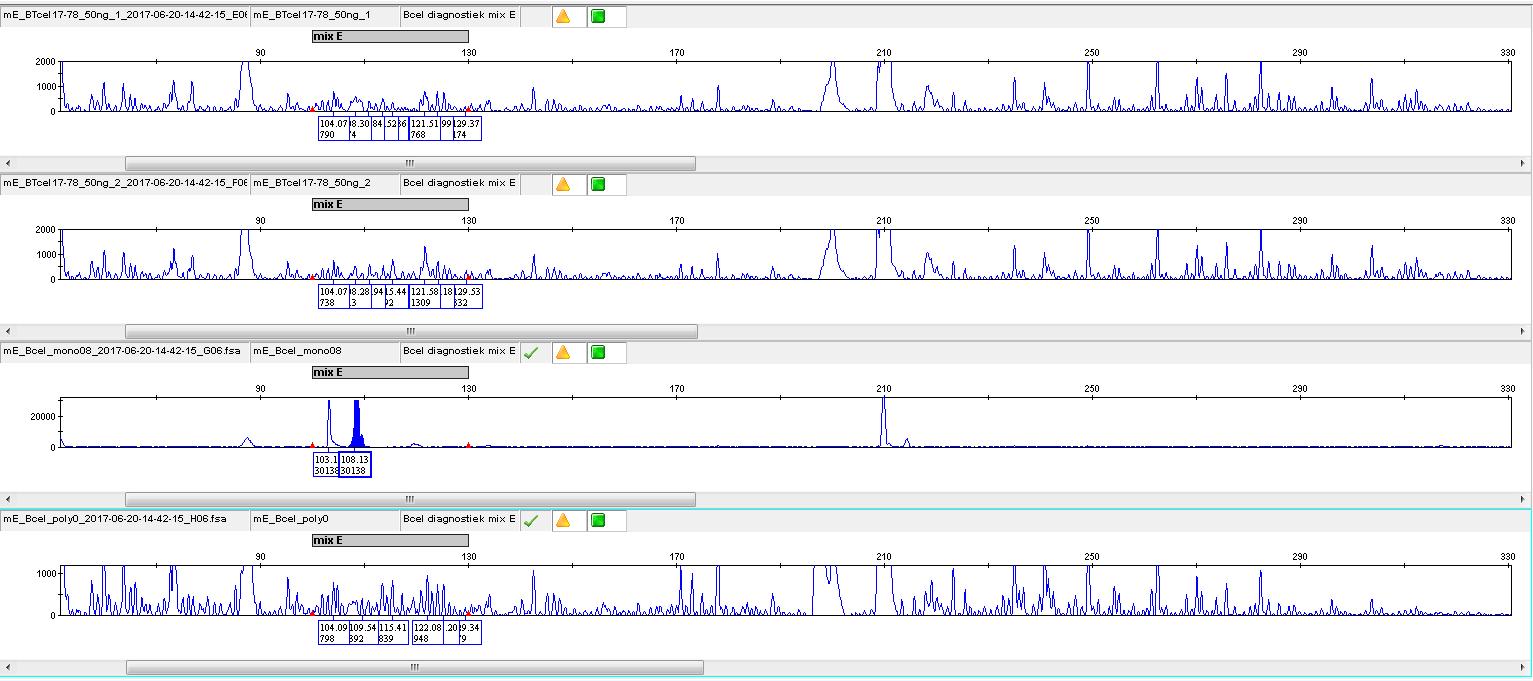
**Figure SF7G**: **B-cell mix E**


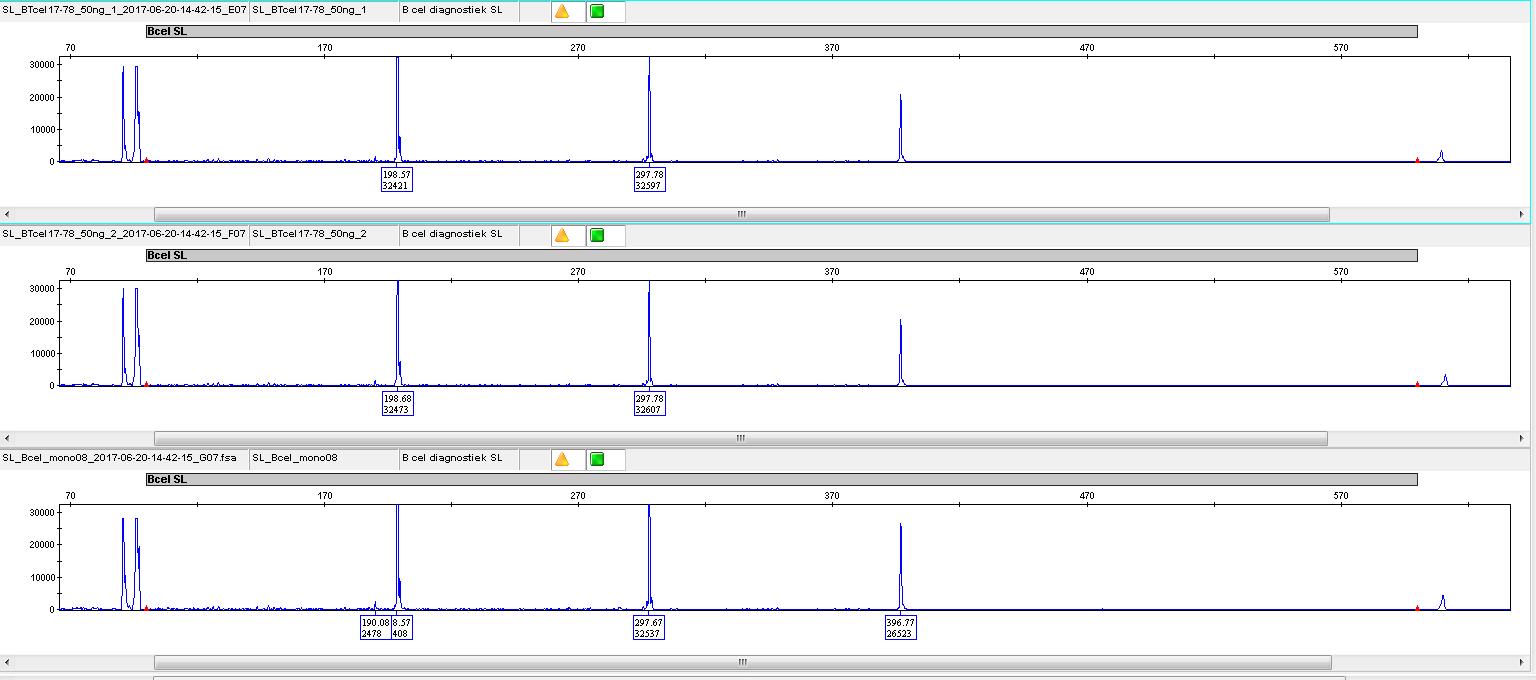


**Figure SF7H**: **B-cell SL**

# SF8: T-cell characterization at age 110 by flow cytometry


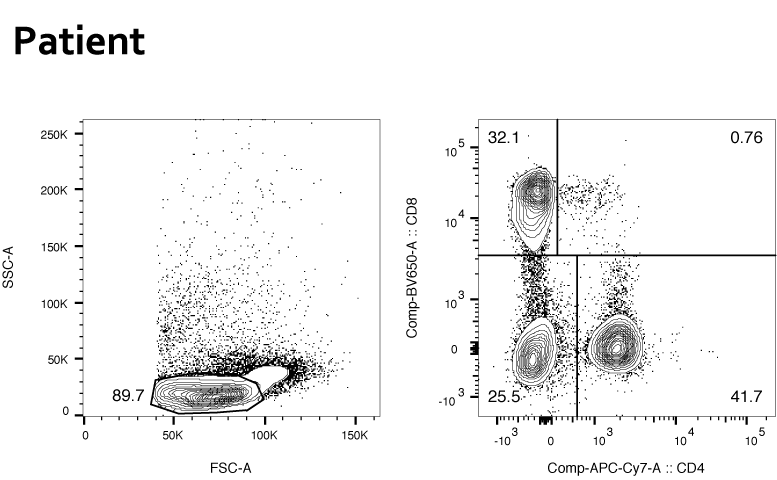


**Figure SF8A**: **Lymphocyte gate in W110:** 44,330 cells enter (left), are gated to 39,771 cells to be further gated (right) to 16,591 (41.7%) CD4+ T-cells, and 12,574 (32.1%) CD8+ T-cells.


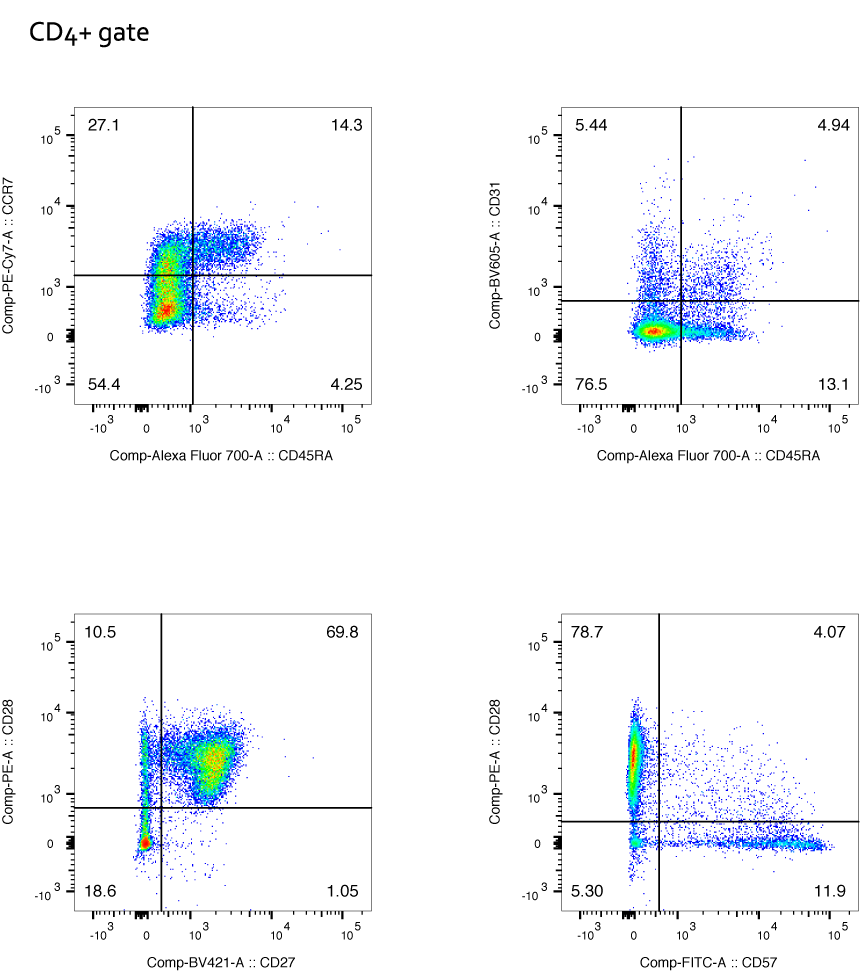


**Figure SF8B: CD4+ Tcell gating in patient W110:** 16,591 CD4+ T-cells are analyzed in each of these pairwise scatters; top left: CCR7-CD45RA+ (CD4+TEMRA); top right CD31+ CD45RA+ (recent thymic immigrants); bottom left: CD27+CD28+; bottom right: CD57+CD28- (senescent).


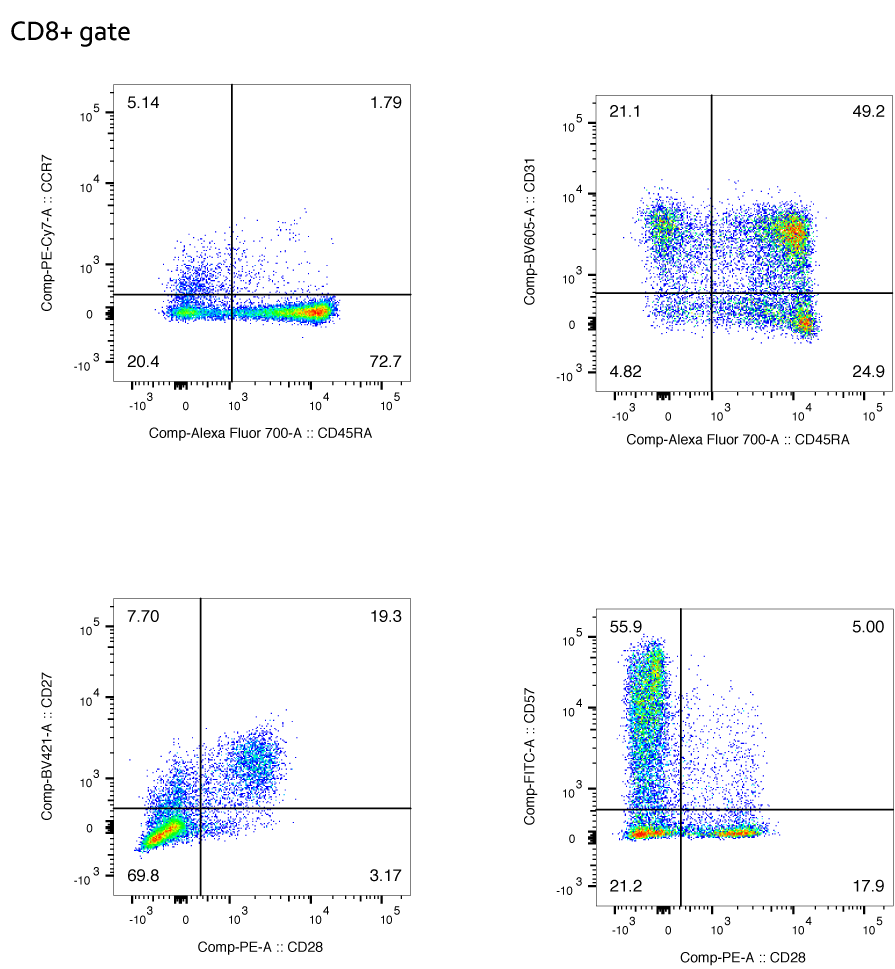
**Figure SF8C: CD8+ Tcell gating in patient W110:** 12,574 CD8+ T-cells are analyzed in each of these pairwise scatters; top left: CCR7-CD45RA+ (CD8+TEMRA); top right CD31+ CD45RA+ (done for consistency); bottom left: CD27+CD28+; bottom right: CD57+CD28- (senescent).


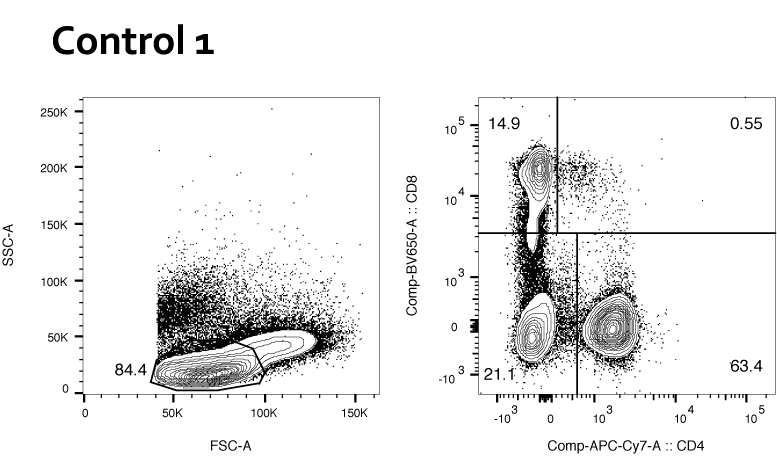


**Figure SF8D**: **Lymphocyte gate in Control 1:** 117,956 cells enter (left), are gated to 99,514 cells to be further gated (right) to 63,105 (63.4%) CD4+ T-cells, and 14,862 (14,9%) CD8+ T-cells.


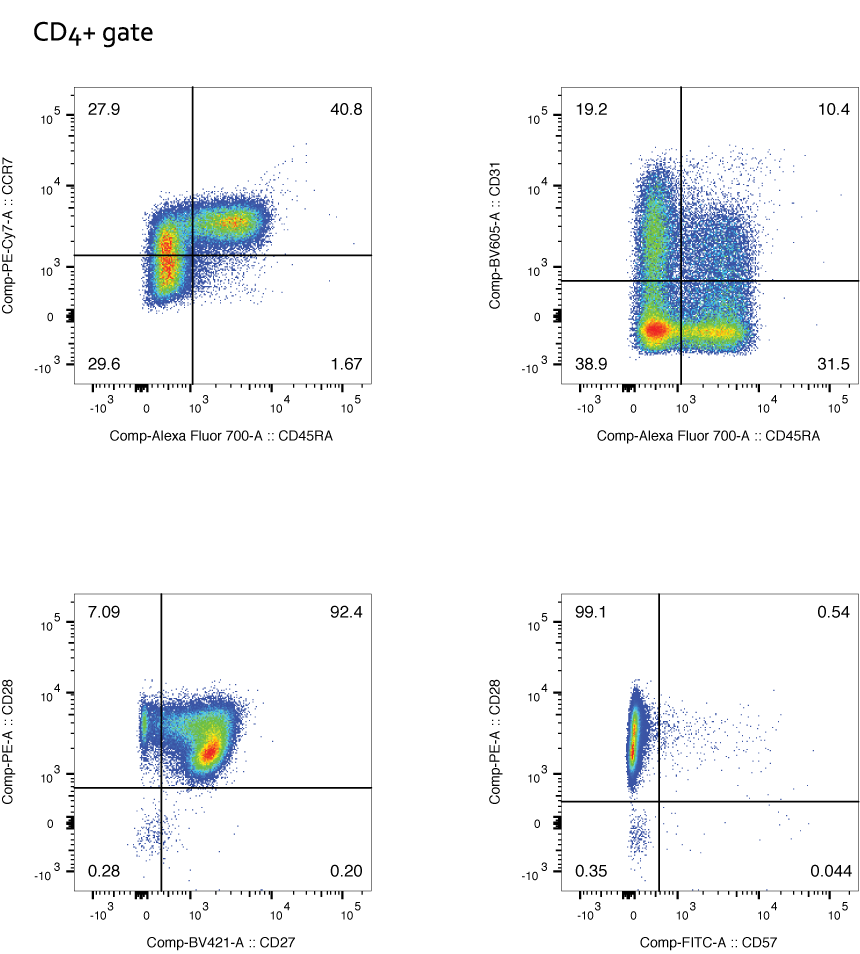
**Figure SF8E: CD4+ Tcell gating in control 1:** 63,105 CD4+ T are analyzed in each of these pairwise scatters; top left: CCR7-CD45RA+ (CD4+TEMRA); top right CD31+ CD45RA+ (recent thymic immigrants); bottom left: CD27+CD28+; bottom right: CD57+CD28- (senescent).


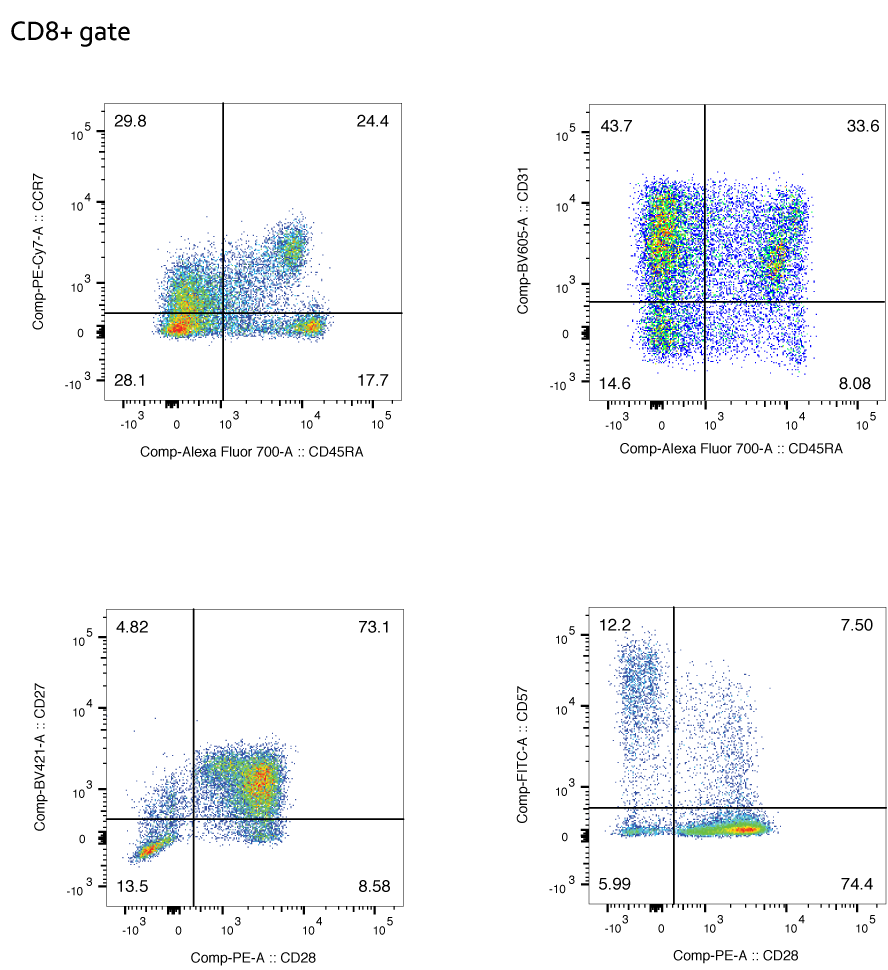
**Figure SF8F: CD8+ Tcell gating in control 1:** 14,862 CD8+ T-cells are analyzed in each of these pairwise scatters; top left: CCR7-CD45RA+ (CD8+TEMRA); top right CD31+ CD45RA+ (done for consistency); bottom left: CD27+CD28+; bottom right: CD57+CD28- (senescent).


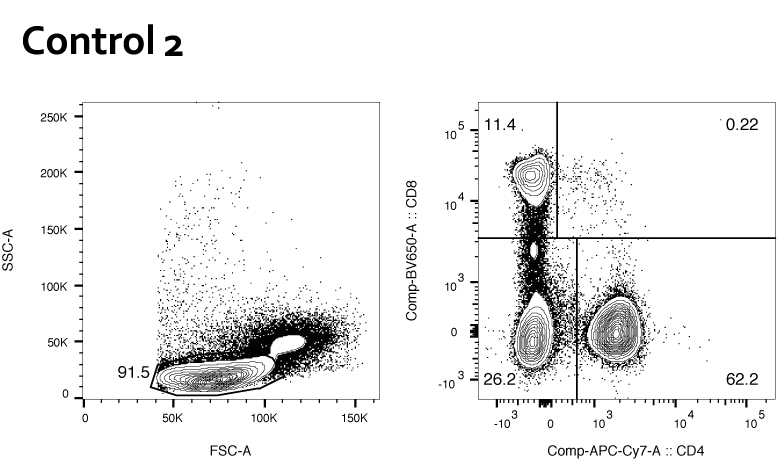


**Figure SF8G**: **Lymphocyte gate in Control 2:** 114,564 cells enter (left), are gated to 104,799 cells to be further gated (right) to 65,196 (62.2%) CD4+ T-cells, and 11,968 (11,4%) CD8+ T-cells.


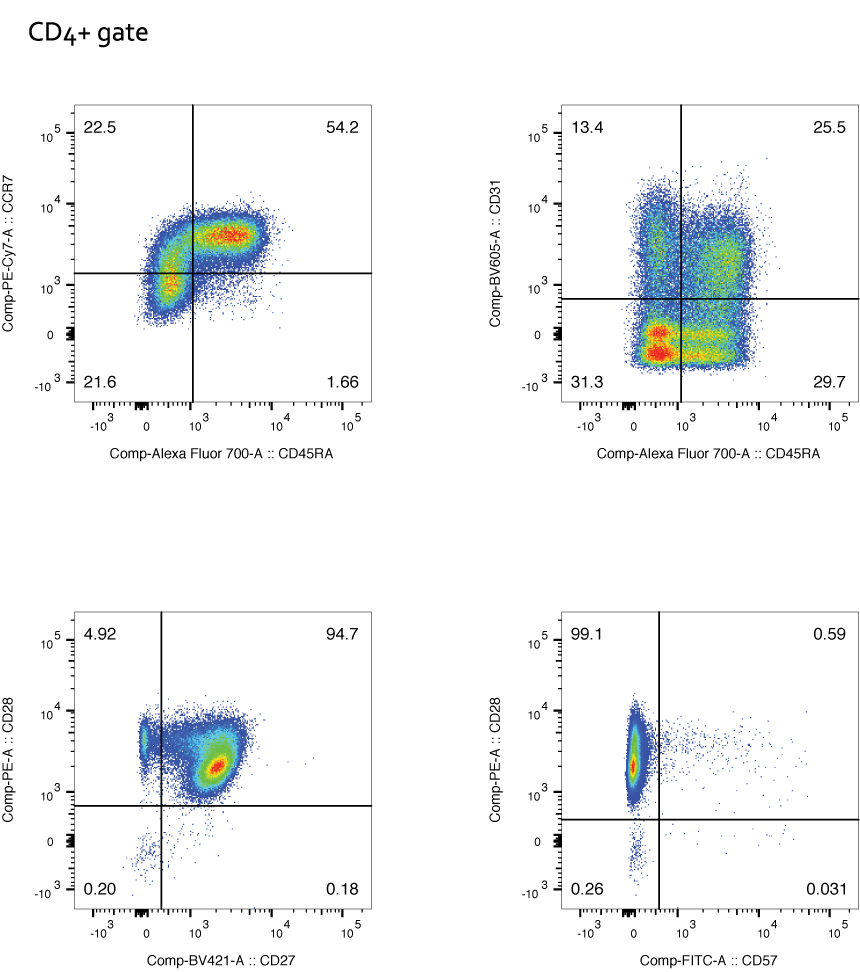


**Figure SF8H: CD4+ Tcell gating in control 2:** 65,196 CD4+ T are analyzed in each of these pairwise scatters; top left: CCR7-CD45RA+ (CD4+TEMRA); top right CD31+ CD45RA+ (recent thymic immigrants); bottom left: CD27+CD28+; bottom right: CD57+CD28- (senescent).


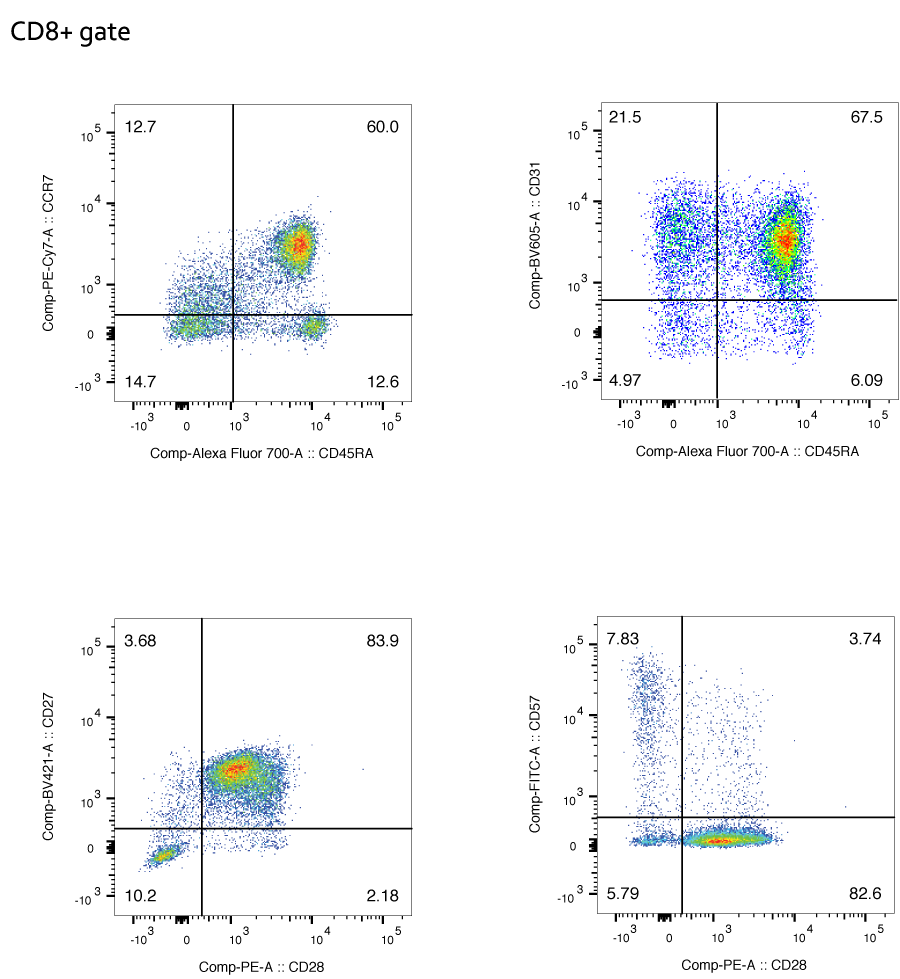


**Figure SF8I: CD8+ Tcell gating in control 2:** 11,968 CD8+ T-cells are analyzed in each of these pairwise scatters; top left: CCR7-CD45RA+ (CD8+TEMRA); top right CD31+ CD45RA+ (done for consistency); bottom left: CD27+CD28+; bottom right: CD57+CD28- (senescent).

# SF9: T-cell characterization at age 111 by flow cytometry


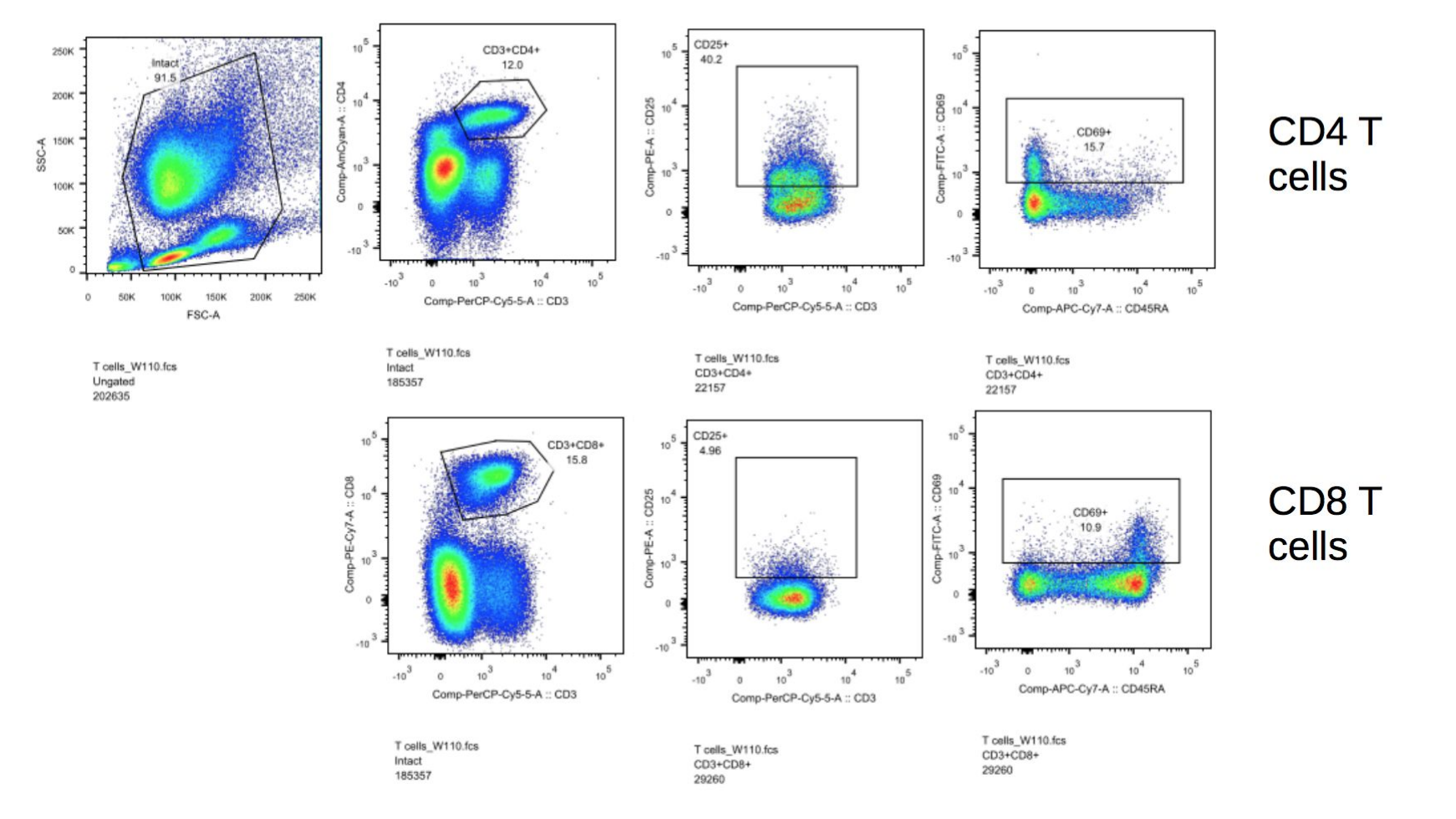


**Figure SF9: T-cell characterization at age 111:** 202,635 peripheral blood cells were gated to 185,357 intact T-cells. 40.2% of the 22,157 CD3^+^CD4^+^ T-cells were also CD25^+^; 15.7% of the CD3^+^CD4^+^ T-cells were also CD69^+^. Similarly, 5.0% of the 29,260 CD3^+^CD8^+^ T-cells were also CD25^+^; 10.9% of the CD3^+^CD8^+^ T-cells were also CD69^+^.

Supplementary Tables - ST

## ST1: 650 Putative Somatic Mutations

| **gvarID** | **ref** | **alt** | **tier** | **VAF** |
| --- | --- | --- | --- | --- |
| chr17__66672231 | C | T | Blood_Tier1 | 0.467 |
| chr20__14934240 | C | T | Blood_Tier1 | 0.488 |
| chr11__123959954 | G | C | Blood_Tier1 | 0.468 |
| chr6__79616543 | A | G | Blood_Tier1 | 0.469 |
| chr6__156444677 | C | T | Blood_Tier1 | 0.438 |
| chr3__110499760 | A | G | Blood_Tier1 | 0.373 |
| chr2__164563143 | A | G | Blood_Tier1 | 0.417 |
| chr5__84789837 | G | A | Blood_Tier1 | 0.412 |
| chr3__192960239 | T | G | Blood_Tier1 | 0.338 |
| chr22__40724186 | G | A | Blood_Tier1 | 0.455 |
| chr2__15721915 | T | C | Blood_Tier1 | 0.369 |
| chr7__29217270 | A | G | Blood_Tier1 | 0.472 |
| chr1__79469387 | T | C | Blood_Tier1 | 0.425 |
| chrX__34276891 | T | G | Blood_Tier1 | 0.420 |
| chr6__150816248 | G | A | Blood_Tier1 | 0.330 |
| chr4__145441371 | A | T | Blood_Tier1 | 0.364 |
| chr10__71833394 | G | A | Blood_Tier1 | 0.333 |
| chr1__231042202 | C | T | Blood_Tier1 | 0.333 |
| chr2__228258480 | G | A | Blood_Tier1 | 0.458 |
| chr9__37342120 | C | T | Blood_Tier1 | 0.376 |
| chr4__24761759 | C | A | Blood_Tier1 | 0.367 |
| chr5__26329781 | G | C | Blood_Tier1 | 0.366 |
| chr13__105958180 | A | G | Blood_Tier1 | 0.500 |
| chr19__29093087 | C | G | Blood_Tier1 | 0.327 |
| chr6__152647273 | G | A | Blood_Tier1 | 0.354 |
| chr7__109719194 | A | G | Blood_Tier2 | 0.500 |
| chr20__25617183 | G | A | Blood_Tier1 | 0.432 |
| chr8__13478301 | T | C | Blood_Tier1 | 0.329 |
| chrX__129344146 | C | T | Blood_Tier1 | 0.389 |
| chr14__25504442 | T | C | Blood_Tier1 | 0.355 |
| chr15__101885527 | T | A | Blood_Tier1 | 0.444 |
| chr9__137633163 | G | A | Blood_Tier1 | 0.397 |
| chr14__64495445 | G | A | Blood_Tier1 | 0.395 |
| chr17__57689194 | T | C | Blood_Tier1 | 0.370 |
| chr2__141131460 | C | T | Blood_Tier1 | 0.393 |
| chr4__137409760 | T | C | Blood_Tier1 | 0.365 |
| chr4__55441256 | C | T | Blood_Tier1 | 0.383 |
| chr6__126113509 | T | C | Blood_Tier1 | 0.373 |
| chr15__77477740 | A | C | Blood_Tier1 | 0.431 |
| chr11__119595518 | C | T | Blood_Tier1 | 0.345 |
| chr9__19516480 | C | A | Blood_Tier1 | 0.299 |
| chr6__44324229 | C | G | Blood_Tier1 | 0.266 |
| chr5__166531089 | C | T | Blood_Tier1 | 0.330 |
| chr7__82293864 | A | G | Blood_Tier1 | 0.333 |
| chr3__176430705 | G | A | Blood_Tier1 | 0.328 |
| chr1__170805330 | A | G | Blood_Tier1 | 0.348 |
| chr5__92280354 | C | A | Blood_Tier1 | 0.346 |
| chr20__39862424 | T | C | Blood_Tier1 | 0.315 |
| chr17__8280395 | G | A | Blood_Tier1 | 0.382 |
| chr4__171755715 | C | G | Blood_Tier1 | 0.325 |
| chr8__28291900 | G | A | Blood_Tier1 | 0.292 |
| chr6__81591019 | C | G | Blood_Tier1 | 0.348 |
| chr4__169089352 | T | A | Blood_Tier1 | 0.333 |
| chr14__26630906 | G | A | Blood_Tier1 | 0.295 |
| chr15__24051898 | C | A | Blood_Tier1 | 0.386 |
| chrX__84392105 | A | G | Blood_Tier1 | 0.342 |
| chr4__30984482 | G | T | Blood_Tier1 | 0.329 |
| chr6__51310525 | C | G | Blood_Tier1 | 0.271 |
| chr12__86416207 | G | A | Blood_Tier1 | 0.384 |
| chr7__122972688 | A | G | Blood_Tier1 | 0.333 |
| chr7__155804425 | A | T | Blood_Tier1 | 0.296 |
| chr11__18060193 | G | T | Blood_Tier1 | 0.435 |
| chr13__35027179 | A | T | Blood_Tier1 | 0.301 |
| chr8__126819256 | T | G | Blood_Tier1 | 0.340 |
| chr9__13732822 | T | A | Blood_Tier1 | 0.258 |
| chr6__69085881 | C | A | Blood_Tier1 | 0.340 |
| chr11__24024971 | G | A | Blood_Tier1 | 0.288 |
| chr8__17407813 | G | A | Blood_Tier1 | 0.286 |
| chr7__40599736 | G | A | Blood_Tier1 | 0.308 |
| chr4__11638973 | C | T | Blood_Tier1 | 0.323 |
| chr11__44526879 | T | C | Blood_Tier1 | 0.373 |
| chr16__53424032 | T | A | Blood_Tier1 | 0.371 |
| chr17__31969569 | C | A | Blood_Tier1 | 0.360 |
| chr3__71590489 | G | T | Blood_Tier1 | 0.255 |
| chr6__87120455 | T | C | Blood_Tier1 | 0.333 |
| chr10__99642604 | C | T | Blood_Tier1 | 0.356 |
| chr10__25106383 | A | C | Blood_Tier1 | 0.315 |
| chr1__237498066 | G | A | Blood_Tier1 | 0.257 |
| chr16__24450792 | G | A | Blood_Tier1 | 0.313 |
| chr8__14220884 | T | C | Blood_Tier1 | 0.342 |
| chr13__57257821 | A | T | Blood_Tier1 | 0.409 |
| chrX__66876515 | T | C | Blood_Tier1 | 0.290 |
| chr20__53722528 | G | A | Blood_Tier1 | 0.333 |
| chr2__38652340 | C | T | Blood_Tier2 | 0.458 |
| chr16__50955663 | T | C | Blood_Tier1 | 0.339 |
| chr1__216608363 | C | T | Blood_Tier1 | 0.296 |
| chr1__37164781 | T | A | Blood_Tier1 | 0.327 |
| chr2__199917885 | A | G | Blood_Tier1 | 0.278 |
| chr1__113621193 | T | G | Blood_Tier1 | 0.355 |
| chr8__125435359 | A | G | Blood_Tier1 | 0.294 |
| chr20__36061512 | G | A | Blood_Tier1 | 0.308 |
| chrX__137615166 | A | T | Blood_Tier1 | 0.308 |
| chr17__51301725 | C | T | Blood_Tier1 | 0.373 |
| chr11__22783931 | T | C | Blood_Tier1 | 0.238 |
| chr10__84756078 | T | C | Blood_Tier2 | 0.429 |
| chr14__50105572 | A | G | Blood_Tier1 | 0.232 |
| chr5__175742143 | A | G | Blood_Tier1 | 0.364 |
| chr1__48839483 | T | G | Blood_Tier1 | 0.302 |
| chr5__42290327 | C | T | Blood_Tier1 | 0.257 |
| chr4__157045476 | T | A | Blood_Tier1 | 0.305 |
| chr4__17857259 | T | A | Blood_Tier1 | 0.277 |
| chr5__88243810 | C | G | Blood_Tier1 | 0.241 |
| chr18__9313391 | A | G | Blood_Tier2 | 0.321 |
| chr16__2645613 | C | T | Blood_Tier1 | 0.282 |
| chr2__200482779 | A | C | Blood_Tier1 | 0.341 |
| chr13__83427582 | G | C | Blood_Tier1 | 0.350 |
| chr10__125225251 | G | T | Blood_Tier1 | 0.235 |
| chr8__34502821 | G | C | Blood_Tier1 | 0.269 |
| chr2__227317709 | T | C | Blood_Tier2 | 0.375 |
| chr4__149877106 | G | A | Blood_Tier1 | 0.191 |
| chr18__1392644 | A | C | Blood_Tier1 | 0.300 |
| chr2__203150320 | T | G | Blood_Tier1 | 0.315 |
| chr2__99565440 | G | T | Blood_Tier2 | 0.238 |
| chr11__62037391 | T | C | Blood_Tier1 | 0.279 |
| chr3__50930603 | G | A | Blood_Tier1 | 0.264 |
| chr5__123025785 | A | G | Blood_Tier1 | 0.316 |
| chr5__140487053 | A | T | Blood_Tier1 | 0.250 |
| chr8__141389872 | T | C | Blood_Tier1 | 0.292 |
| chrX__18897311 | C | G | Blood_Tier1 | 0.224 |
| chr22__44385037 | C | G | Blood_Tier1 | 0.232 |
| chr14__21773358 | G | A | Blood_Tier1 | 0.311 |
| chr3__83880717 | C | A | Blood_Tier1 | 0.222 |
| chr2__198609762 | G | A | Blood_Tier1 | 0.274 |
| chr6__100169580 | C | T | Blood_Tier1 | 0.308 |
| chrX__132397792 | A | C | Blood_Tier1 | 0.237 |
| chr3__164622904 | A | T | Blood_Tier1 | 0.241 |
| chr17__68940187 | C | T | Blood_Tier1 | 0.333 |
| chr4__148542277 | C | A | Blood_Tier1 | 0.219 |
| chr18__60094786 | T | C | Blood_Tier2 | 0.182 |
| chr1__222054608 | G | A | Blood_Tier1 | 0.213 |
| chr15__94305134 | T | C | Blood_Tier1 | 0.265 |
| chr2__190340213 | T | C | Blood_Tier2 | 0.200 |
| chr4__169675257 | G | A | Blood_Tier1 | 0.147 |
| chr12__79160128 | C | A | Blood_Tier1 | 0.313 |
| chr11__55523417 | C | A | Blood_Tier1 | 0.221 |
| chr16__7739039 | G | A | Blood_Tier1 | 0.200 |
| chr20__53456479 | G | A | Blood_Tier1 | 0.213 |
| chr7__73217590 | G | A | Blood_Tier1 | 0.128 |
| chr3__141002881 | A | G | Blood_Tier2 | 0.250 |
| chr3__77327697 | A | C | Blood_Tier2 | 0.474 |
| chr2__61401283 | A | G | Blood_Tier1 | 0.314 |
| chr2__227183785 | C | T | Blood_Tier1 | 0.391 |
| chr6__16390236 | T | C | Blood_Tier1 | 0.321 |
| chr2__136630897 | T | A | Blood_Tier1 | 0.250 |
| chrX__50069526 | T | C | Blood_Tier2 | 0.235 |
| chr9__100480485 | G | A | Blood_Tier1 | 0.277 |
| chr19__56255629 | A | G | Blood_Tier1 | 0.322 |
| chr18__75296947 | T | C | Blood_Tier1 | 0.167 |
| chr1__86284096 | C | T | Blood_Tier1 | 0.204 |
| chr12__59303326 | T | C | Blood_Tier1 | 0.258 |
| chr11__3766585 | A | G | Blood_Tier1 | 0.286 |
| chr12__81010387 | A | G | Blood_Tier2 | 0.200 |
| chr4__102280086 | T | C | Blood_Tier1 | 0.229 |
| chrX__114116246 | A | C | Blood_Tier1 | 0.305 |
| chr1__53590585 | C | T | Blood_Tier1 | 0.225 |
| chr2__176211302 | C | T | Blood_Tier1 | 0.317 |
| chr7__45276483 | G | T | Blood_Tier1 | 0.197 |
| chrX__33045447 | A | G | Blood_Tier1 | 0.289 |
| chr4__24092455 | C | T | Blood_Tier1 | 0.184 |
| chr15__36040233 | G | A | Blood_Tier1 | 0.292 |
| chr10__131873809 | G | C | Blood_Tier1 | 0.222 |
| chr2__123856347 | C | G | Blood_Tier1 | 0.489 |
| chr5__167871848 | A | G | Blood_Tier2 | 0.200 |
| chr3__147908859 | T | C | Blood_Tier1 | 0.333 |
| chr14__101629779 | A | G | Blood_Tier2 | 0.160 |
| chr12__80759750 | A | G | Blood_Tier1 | 0.246 |
| chr4__21814933 | T | C | Blood_Tier2 | 0.182 |
| chr9__137585155 | T | G | Blood_Tier1 | 0.238 |
| chr8__47359871 | C | T | Blood_Tier2 | 0.333 |
| chr2__28226905 | G | C | Blood_Tier1 | 0.294 |
| chr6__121901998 | G | A | Blood_Tier1 | 0.184 |
| chr7__103470996 | T | A | Blood_Tier2 | 0.444 |
| chr10__86358071 | C | T | Blood_Tier1 | 0.239 |
| chr15__26802929 | A | G | Blood_Tier2 | 0.304 |
| chr22__34167420 | C | T | Blood_Tier1 | 0.234 |
| chr9__8517886 (PTPRD) | G | A | Blood_Tier1 | 0.198 |
| chr21__27807421 | T | C | Blood_Tier1 | 0.293 |
| chr16__26597017 | A | G | Blood_Tier1 | 0.162 |
| chr10__53125352 | A | T | Blood_Tier1 | 0.274 |
| chr5__34012260 | A | G | Blood_Tier1 | 0.263 |
| chr5__151631087 | G | A | Blood_Tier1 | 0.167 |
| chr13__36278145 | C | T | Blood_Tier2 | 0.121 |
| chr11__85068516 | G | A | Blood_Tier1 | 0.357 |
| chr3__31655114 | T | C | Blood_Tier2 | 0.174 |
| chr22__46538743 | G | A | Blood_Tier2 | 0.372 |
| chr17__67216242 | T | C | Blood_Tier2 | 0.111 |
| chrX__34047538 | A | T | Blood_Tier1 | 0.280 |
| chr20__46878505 | C | A | Blood_Tier1 | 0.325 |
| chr9__84717849 | G | T | Blood_Tier1 | 0.272 |
| chr5__79447974 | C | T | Blood_Tier1 | 0.227 |
| chr5__116573374 | T | C | Blood_Tier2 | 0.133 |
| chr5__84711280 | A | T | Blood_Tier1 | 0.155 |
| chr14__35339308 | G | C | Blood_Tier1 | 0.188 |
| chr4__159243915 | A | G | Blood_Tier1 | 0.337 |
| chr7__131572995 | C | T | Blood_Tier1 | 0.216 |
| chr16__20829538 | T | C | Blood_Tier2 | 0.148 |
| chr21__31669351 | T | G | Blood_Tier2 | 0.242 |
| chrX__85300646 | T | C | Blood_Tier1 | 0.114 |
| chr10__95590751 | G | A | Blood_Tier1 | 0.138 |
| chr3__12400772 | C | T | Blood_Tier2 | 0.143 |
| chr6__160372588 | C | A | Blood_Tier1 | 0.316 |
| chr20__20453847 | G | A | Blood_Tier1 | 0.258 |
| chr16__18023205 | G | A | Blood_Tier1 | 0.253 |
| chrX__147904819 | C | G | Blood_Tier1 | 0.172 |
| chr15__86765022 | A | T | Blood_Tier1 | 0.103 |
| chr6__20794608 | A | G | Blood_Tier1 | 0.279 |
| chr1__220183809 | A | G | Blood_Tier2 | 0.108 |
| chr8__54468375 | T | C | Blood_Tier1 | 0.232 |
| chr1__186581699 | T | A | Blood_Tier1 | 0.216 |
| chr12__29141533 | C | T | Blood_Tier1 | 0.217 |
| chr8__41745949 | G | A | Blood_Tier1 | 0.286 |
| chr6__73053278 | T | C | Blood_Tier2 | 0.357 |
| chr11__134641812 | G | T | Blood_Tier1 | 0.218 |
| chr10__78249857 | T | C | Blood_Tier2 | 0.308 |
| chr11__4399767 | A | T | Blood_Tier1 | 0.184 |
| chr3__166901364 | G | A | Blood_Tier1 | 0.303 |
| chr11__55240858 | T | C | Blood_Tier2 | 0.121 |
| chr11__91653831 | C | T | Blood_Tier1 | 0.333 |
| chr19__52010257 | G | A | Blood_Tier1 | 0.281 |
| chr20__57909208 | C | T | Blood_Tier1 | 0.174 |
| chr5__41912087 | T | C | Blood_Tier3 | 0.500 |
| chr1__244390672 | T | C | Blood_Tier1 | 0.253 |
| chr4__107474761 | A | T | Blood_Tier2 | 0.118 |
| chr5__84069060 | C | T | Blood_Tier3 | 0.444 |
| chr7__42936683 | A | G | Blood_Tier2 | 0.200 |
| chr18__75948056 | C | G | Blood_Tier2 | 0.160 |
| chr12__32994254 | G | A | Blood_Tier2 | 0.480 |
| chr7__293540 | C | T | Blood_Tier2 | 0.450 |
| chr7__103470997 | T | A | Blood_Tier3 | 0.500 |
| chr7__98695779 | A | G | Blood_Tier2 | 0.235 |
| chr8__27484960 | C | T | Blood_Tier1 | 0.160 |
| chr11__62926293 | G | A | Blood_Tier2 | 0.154 |
| chr15__73296442 | C | T | Blood_Tier1 | 0.147 |
| chr11__37183794 | A | G | Blood_Tier1 | 0.127 |
| chr6__166226981 | G | A | Blood_Tier1 | 0.116 |
| chr16__6613470 | G | A | Blood_Tier1 | 0.230 |
| chr10__109656568 | C | T | Blood_Tier1 | 0.242 |
| chr5__130022910 | G | A | Blood_Tier1 | 0.259 |
| chr7__126483539 | G | C | Blood_Tier2 | 0.235 |
| chr7__78431782 | C | T | Blood_Tier1 | 0.222 |
| chr21__31669352 | G | T | Blood_Tier2 | 0.229 |
| chr21__35721670 | G | A | Blood_Tier1 | 0.228 |
| chr1__201313101 | A | T | Blood_Tier1 | 0.085 |
| chr1__7358343 | T | C | Blood_Tier1 | 0.242 |
| chr11__133074959 | G | C | Blood_Tier2 | 0.176 |
| chr5__133385008 | C | A | Blood_Tier2 | 0.255 |
| chr19__20809490 | C | A | Blood_Tier2 | 0.484 |
| chr7__119749743 | G | T | Blood_Tier2 | 0.225 |
| chr3__20628725 | T | C | Blood_Tier3 | 0.500 |
| chr9__85325108 | C | G | Blood_Tier2 | 0.167 |
| chr4__115824013 | G | A | Blood_Tier2 | 0.136 |
| chr13__112600346 | G | T | Blood_Tier1 | 0.280 |
| chr3__130030623 | A | T | Blood_Tier2 | 0.286 |
| chr12__43843430 | A | G | Blood_Tier2 | 0.147 |
| chr16__52979969 | A | G | Blood_Tier2 | 0.190 |
| chr22__29259269 | C | T | Blood_Tier2 | 0.320 |
| chrX__14959214 | G | A | Blood_Tier1 | 0.078 |
| chr3__52315833 | T | C | Blood_Tier1 | 0.350 |
| chr2__8958069 | T | C | Blood_Tier2 | 0.100 |
| chr18__70491670 | A | G | Blood_Tier2 | 0.271 |
| chr2__149796259 | C | T | Blood_Tier1 | 0.111 |
| chr3__165184061 | T | C | Blood_Tier2 | 0.235 |
| chr10__20491854 | G | A | Blood_Tier1 | 0.119 |
| chr8__54188756 | A | G | Blood_Tier2 | 0.375 |
| chr5__120286007 | C | A | Blood_Tier2 | 0.328 |
| chr1__180644884 | G | A | Blood_Tier1 | 0.108 |
| chr19__56322749 | G | A | Blood_Tier1 | 0.164 |
| chr5__68689451 | A | G | Blood_Tier2 | 0.133 |
| chr14__82551829 | T | A | Blood_Tier1 | 0.254 |
| chr16__87305621 | G | A | Blood_Tier1 | 0.230 |
| chr8__116308909 | T | C | Blood_Tier2 | 0.114 |
| chr12__90867035 | C | G | Blood_Tier2 | 0.200 |
| chr9__88013515 | G | T | Blood_Tier2 | 0.255 |
| chr19__48149785 | G | A | Blood_Tier1 | 0.075 |
| chr17__29741611 | G | A | Blood_Tier2 | 0.245 |
| chr3__21941024 | C | A | Blood_Tier1 | 0.244 |
| chr10__10034173 | C | T | Blood_Tier2 | 0.139 |
| chr6__146584713 | T | C | Blood_Tier2 | 0.154 |
| chr3__138121382 | G | C | Blood_Tier2 | 0.232 |
| chr11__133213386 | A | G | Blood_Tier1 | 0.169 |
| chr14__33343977 | T | A | Blood_Tier2 | 0.410 |
| chrX__109490016 | T | C | Blood_Tier1 | 0.228 |
| chr5__144949915 | C | T | Blood_Tier1 | 0.213 |
| chr2__63601769 | T | C | Blood_Tier2 | 0.133 |
| chr4__184500714 | T | G | Blood_Tier2 | 0.375 |
| chr8__69906311 | G | A | Blood_Tier1 | 0.108 |
| chr11__103189459 | A | G | Blood_Tier3 | 0.273 |
| chr20__38230941 | G | A | Blood_Tier1 | 0.337 |
| chr7__129772266 | G | A | Blood_Tier1 | 0.139 |
| chr8__138683067 | C | G | Blood_Tier2 | 0.182 |
| chr20__36457105 | G | A | Blood_Tier1 | 0.294 |
| chr1__233558620 | T | C | Blood_Tier2 | 0.125 |
| chr6__51587361 | G | A | Blood_Tier1 | 0.095 |
| chr14__31631966 | T | C | Blood_Tier2 | 0.159 |
| chr17__72851459 | C | T | Blood_Tier2 | 0.123 |
| chr7__119749744 | G | T | Blood_Tier2 | 0.195 |
| chr11__21892126 | A | G | Blood_Tier2 | 0.125 |
| chr1__179142889 | A | G | Blood_Tier2 | 0.212 |
| chr2__11641476 | G | A | Blood_Tier2 | 0.104 |
| chr11__115664774 | C | T | Blood_Tier2 | 0.328 |
| chr16__15603964 | G | C | Blood_Tier2 | 0.304 |
| chr7__142480345 | C | A | Blood_Tier2 | 0.108 |
| chr12__95625935 | T | C | Blood_Tier3 | 0.429 |
| chr21__47278674 | G | A | Blood_Tier2 | 0.194 |
| chr13__90075654 | A | G | Blood_Tier3 | 0.286 |
| chr7__83446721 | C | T | Blood_Tier1 | 0.265 |
| chr4__171153608 | A | G | Blood_Tier2 | 0.094 |
| chr4__171058550 | C | T | Blood_Tier1 | 0.194 |
| chr6__14860391 | G | T | Blood_Tier1 | 0.301 |
| chr2__116215885 | G | A | Blood_Tier2 | 0.143 |
| chr7__130645986 | G | A | Blood_Tier2 | 0.292 |
| chr20__18999983 | A | G | Blood_Tier2 | 0.118 |
| chr18__5952052 | A | G | Blood_Tier3 | 0.273 |
| chr11__44366993 | C | T | Blood_Tier1 | 0.240 |
| chr3__153752188 | C | G | Blood_Tier2 | 0.200 |
| chr2__97487365 | T | C | Blood_Tier2 | 0.109 |
| chr2__211063832 | T | C | Blood_Tier2 | 0.167 |
| chr7__141023239 | A | T | Blood_Tier2 | 0.317 |
| chr7__158650954 | A | G | Blood_Tier2 | 0.167 |
| chr11__63759135 | G | A | Blood_Tier2 | 0.224 |
| chr15__40074575 | T | C | Blood_Tier2 | 0.229 |
| chr6__11618300 | G | A | Blood_Tier2 | 0.133 |
| chr12__87948328 | C | T | Blood_Tier2 | 0.085 |
| chr7__100913097 | G | T | Blood_Tier2 | 0.086 |
| chr4__33840364 | A | G | Blood_Tier2 | 0.143 |
| chr2__208166004 | A | G | Blood_Tier2 | 0.194 |
| chr1__31427695 | A | G | Blood_Tier2 | 0.102 |
| chr5__24947460 | T | C | Blood_Tier2 | 0.125 |
| chr3__138220877 | C | T | Blood_Tier2 | 0.160 |
| chr4__131585241 | A | G | Blood_Tier2 | 0.208 |
| chr7__100913094 | A | G | Blood_Tier2 | 0.093 |
| chr10__113806265 | G | A | Blood_Tier2 | 0.133 |
| chr2__185184871 | C | T | Blood_Tier2 | 0.279 |
| chr2__213904794 | A | G | Blood_Tier3 | 0.182 |
| chrX__102549956 | G | A | Blood_Tier3 | 0.250 |
| chr2__29007303 | T | C | Blood_Tier2 | 0.167 |
| chr17__63146103 | T | C | Blood_Tier2 | 0.314 |
| chr18__74348273 | T | C | Blood_Tier2 | 0.099 |
| chr2__183127666 | T | G | Blood_Tier2 | 0.143 |
| chr10__93546985 | G | A | Blood_Tier2 | 0.340 |
| chr21__27791896 | C | T | Blood_Tier2 | 0.222 |
| chrX__126808708 | C | A | Blood_Tier2 | 0.250 |
| chr2__148198348 | G | T | Blood_Tier2 | 0.279 |
| chr8__109617037 | T | C | Blood_Tier2 | 0.208 |
| chrX__34206854 | C | T | Blood_Tier2 | 0.154 |
| chr13__81353501 | G | T | Blood_Tier2 | 0.148 |
| chr10__58835229 | G | C | Blood_Tier2 | 0.190 |
| chr14__77817270 | G | A | Blood_Tier2 | 0.195 |
| chr1__77567898 | G | C | Blood_Tier2 | 0.286 |
| chr1__100321512 | A | G | Blood_Tier2 | 0.182 |
| chr8__99588635 | G | C | Blood_Tier2 | 0.233 |
| chr11__35956176 | G | A | Blood_Tier2 | 0.081 |
| chr10__55246987 | C | T | Blood_Tier2 | 0.429 |
| chr13__97517961 | C | T | Blood_Tier2 | 0.123 |
| chr2__28920772 | G | A | Blood_Tier2 | 0.103 |
| chr5__142792230 | C | G | Blood_Tier2 | 0.250 |
| chr2__42979897 | C | T | Blood_Tier2 | 0.333 |
| chr9__118893196 | A | C | Blood_Tier2 | 0.136 |
| chr2__164553954 | G | A | Blood_Tier2 | 0.244 |
| chr4__32354940 | A | G | Blood_Tier2 | 0.167 |
| chr6__147249464 | A | T | Blood_Tier2 | 0.083 |
| chrX__152227031 | G | A | Blood_Tier3 | 0.174 |
| chr7__153381781 | C | T | Blood_Tier2 | 0.119 |
| chr5__145397325 | A | G | Blood_Tier2 | 0.237 |
| chr7__142480349 | A | T | Blood_Tier2 | 0.103 |
| chr17__52417510 | A | G | Blood_Tier2 | 0.127 |
| chr12__104001108 | C | A | Blood_Tier2 | 0.196 |
| chr2__123856244 | A | G | Blood_Tier2 | 0.281 |
| chr2__166298204 | T | C | Blood_Tier2 | 0.087 |
| chr15__74911715 | A | G | Blood_Tier2 | 0.125 |
| chr5__77992219 | C | T | Blood_Tier2 | 0.171 |
| chr18__5194040 | A | G | Blood_Tier2 | 0.255 |
| chr8__79944479 | T | C | Blood_Tier2 | 0.138 |
| chrX__126940482 | G | A | Blood_Tier2 | 0.233 |
| chr4__126361802 | T | A | Blood_Tier3 | 0.125 |
| chr2__183771999 | A | T | Blood_Tier2 | 0.231 |
| chr7__54540043 | C | T | Blood_Tier2 | 0.280 |
| chr13__75165964 | G | A | Blood_Tier3 | 0.133 |
| chr3__52977475 | G | A | Blood_Tier2 | 0.237 |
| chr3__158785637 | T | C | Blood_Tier2 | 0.186 |
| chr1__236402269 | A | G | Blood_Tier2 | 0.194 |
| chrX__14888241 | T | C | Blood_Tier2 | 0.232 |
| chr11__82829818 | C | T | Blood_Tier2 | 0.320 |
| chr12__59238155 | C | T | Blood_Tier2 | 0.354 |
| chr8__97577593 | G | A | Blood_Tier3 | 0.143 |
| chr12__132446865 | A | G | Blood_Tier3 | 0.125 |
| chr4__123669774 | C | A | Blood_Tier2 | 0.239 |
| chr2__183127665 | T | G | Blood_Tier2 | 0.162 |
| chr11__24685015 | T | C | Blood_Tier2 | 0.146 |
| chrX__143418060 | T | C | Blood_Tier3 | 0.118 |
| chr12__11210419 | A | C | Blood_Tier2 | 0.063 |
| chr12__8149812 | C | T | Blood_Tier2 | 0.222 |
| chr7__88148508 | A | G | Blood_Tier3 | 0.375 |
| chr12__11210420 | G | A | Blood_Tier2 | 0.063 |
| chrX__99060469 | G | A | Blood_Tier2 | 0.211 |
| chr16__65890985 | T | C | Blood_Tier3 | 0.333 |
| chr6__82175337 | A | G | Blood_Tier3 | 0.125 |
| chr12__33853021 | C | T | Blood_Tier2 | 0.178 |
| chrX__97811601 | T | A | Blood_Tier2 | 0.100 |
| chr14__71731010 | G | T | Blood_Tier2 | 0.089 |
| chrX__130195968 | C | A | Blood_Tier2 | 0.065 |
| chr6__32432565 | C | T | Blood_Tier2 | 0.102 |
| chr6__84964258 | T | C | Blood_Tier3 | 0.160 |
| chr2__123918052 | T | C | Blood_Tier3 | 0.194 |
| chr13__85569601 | T | G | Blood_Tier3 | 0.190 |
| chr9__120691738 | C | T | Blood_Tier2 | 0.155 |
| chr9__118123925 | C | T | Blood_Tier2 | 0.197 |
| chr10__126339325 | T | C | Blood_Tier2 | 0.310 |
| chr7__57634252 | C | G | Blood_Tier2 | 0.087 |
| chrX__123798197 | A | T | Blood_Tier2 | 0.129 |
| chr1__63581312 | T | C | Blood_Tier2 | 0.115 |
| chr21__39764905 | T | C | Blood_Tier2 | 0.315 |
| chr13__20371060 | C | A | Blood_Tier2 | 0.089 |
| chr3__179937559 | T | C | Blood_Tier3 | 0.214 |
| chr6__70361621 | T | A | Blood_Tier2 | 0.106 |
| chr20__40341458 | C | A | Blood_Tier2 | 0.170 |
| chr1__180893060 | C | T | Blood_Tier2 | 0.281 |
| chr1__43017274 | A | G | Blood_Tier3 | 0.133 |
| chr16__88255777 | C | T | Blood_Tier2 | 0.214 |
| chr1__220238441 | C | T | Blood_Tier3 | 0.106 |
| chr12__14130513 | C | T | Blood_Tier3 | 0.098 |
| chr6__156071675 | G | A | Blood_Tier3 | 0.222 |
| chr3__38961324 | T | C | Blood_Tier2 | 0.214 |
| chr7__122029852 | A | G | Blood_Tier3 | 0.214 |
| chr2__95585053 | G | C | Blood_Tier2 | 0.105 |
| chr21__44826808 | A | G | Blood_Tier3 | 0.190 |
| chr4__175404073 | T | C | Blood_Tier3 | 0.235 |
| chr6__32432575 | T | C | Blood_Tier3 | 0.091 |
| chr11__94335617 | C | T | Blood_Tier3 | 0.176 |
| chr11__102729686 | C | T | Blood_Tier2 | 0.318 |
| chr8__61215776 | C | T | Blood_Tier3 | 0.286 |
| chr14__93904956 | T | C | Blood_Tier2 | 0.349 |
| chr1__35656873 | A | T | Blood_Tier2 | 0.124 |
| chr5__166164023 | G | A | Blood_Tier2 | 0.247 |
| chr5__38148040 | G | A | Blood_Tier2 | 0.307 |
| chr1__107865400 | C | T | Blood_Tier2 | 0.271 |
| chr4__45093115 | A | C | Blood_Tier3 | 0.121 |
| chr7__43402191 | C | T | Blood_Tier2 | 0.135 |
| chr5__11265045 | T | A | Blood_Tier3 | 0.212 |
| chr3__25263891 | T | A | Blood_Tier2 | 0.164 |
| chrX__136332201 | T | C | Blood_Tier2 | 0.152 |
| chr11__17058370 | G | C | Blood_Tier2 | 0.260 |
| chr2__222988298 | C | A | Blood_Tier2 | 0.141 |
| chr1__203505501 | C | A | Blood_Tier2 | 0.208 |
| chr9__127654540 | A | C | Blood_Tier2 | 0.339 |
| chrX__107726986 | T | C | Blood_Tier2 | 0.333 |
| chr2__160880663 | C | T | Blood_Tier3 | 0.125 |
| chr17__61953832 | T | C | Blood_Tier2 | 0.074 |
| chr18__11198650 | T | C | Blood_Tier2 | 0.281 |
| chr4__161063756 | T | C | Blood_Tier2 | 0.265 |
| chr2__90265077 | A | G | Blood_Tier2 | 0.093 |
| chr3__37745882 | G | A | Blood_Tier2 | 0.114 |
| chr7__142153278 | T | C | Blood_Tier2 | 0.091 |
| chr10__59279600 | T | C | Blood_Tier3 | 0.273 |
| chr21__41193413 | C | A | Blood_Tier2 | 0.313 |
| chr7__29940225 | C | T | Blood_Tier3 | 0.394 |
| chr2__194613483 | A | G | Blood_Tier3 | 0.158 |
| chr13__99419311 | G | A | Blood_Tier2 | 0.100 |
| chr13__106148454 | A | G | Blood_Tier2 | 0.172 |
| chr2__237158632 | C | A | Blood_Tier2 | 0.089 |
| chr2__222988297 | C | A | Blood_Tier2 | 0.139 |
| chr2__151857888 | A | T | Blood_Tier3 | 0.219 |
| chr10__27239514 | G | A | Blood_Tier2 | 0.127 |
| chr4__91726950 | G | A | Blood_Tier3 | 0.114 |
| chrX__49972551 | C | T | Blood_Tier2 | 0.078 |
| chr3__94257342 | C | T | Blood_Tier2 | 0.233 |
| chr3__163911788 | A | T | Blood_Tier2 | 0.138 |
| chr20__33824956 | C | T | Blood_Tier2 | 0.234 |
| chrX__121955937 | C | T | Blood_Tier3 | 0.119 |
| chr1__91688375 | T | G | Blood_Tier2 | 0.082 |
| chr8__127828459 | A | G | Blood_Tier3 | 0.250 |
| chr19__20799880 | A | G | Blood_Tier2 | 0.092 |
| chr19__20799879 | G | A | Blood_Tier2 | 0.092 |
| chrX__49972548 | G | A | Blood_Tier2 | 0.094 |
| chr12__11176428 | G | C | Blood_Tier2 | 0.079 |
| chr8__21514835 | T | C | Blood_Tier3 | 0.214 |
| chr2__25469028 (DNMT3A) | C | T | Blood_Tier3 | 0.224 |
| chr1__72999818 | A | G | Blood_Tier3 | 0.375 |
| chr14__30593452 | A | C | Blood_Tier3 | 0.161 |
| chr8__138681711 | A | T | Blood_Tier3 | 0.263 |
| chrX__61884169 | G | A | Blood_Tier2 | 0.101 |
| chrX__65369257 | T | G | Blood_Tier3 | 0.159 |
| chr7__14288496 | G | A | Blood_Tier3 | 0.071 |
| chr12__48861370 | C | T | Blood_Tier3 | 0.214 |
| chr3__54633194 | C | T | Blood_Tier2 | 0.097 |
| chr2__96516676 | G | C | Blood_Tier2 | 0.194 |
| chr12__26365989 | T | C | Blood_Tier2 | 0.208 |
| chr8__138681710 | A | T | Blood_Tier3 | 0.263 |
| chr9__129576038 | G | T | Blood_Tier3 | 0.241 |
| chr5__174418500 | T | C | Blood_Tier2 | 0.068 |
| chr1__27210525 | C | T | Blood_Tier3 | 0.118 |
| chr4__184500715 | G | T | Blood_Tier3 | 0.357 |
| chr7__108229549 | T | A | Blood_Tier3 | 0.214 |
| chrX__121955915 | A | C | Blood_Tier3 | 0.100 |
| chr3__36839096 | C | T | Blood_Tier3 | 0.200 |
| chr3__192399169 | G | T | Blood_Tier2 | 0.217 |
| chr16__65320183 | G | T | Blood_Tier3 | 0.122 |
| chr18__52712073 | A | G | Blood_Tier3 | 0.097 |
| chr9__86052888 | T | A | Blood_Tier2 | 0.203 |
| chr15__26686717 | G | T | Blood_Tier3 | 0.316 |
| chr10__27241290 | A | C | Blood_Tier2 | 0.101 |
| chr6__116473002 | T | C | Blood_Tier3 | 0.158 |
| chr11__97875462 | A | G | Blood_Tier3 | 0.333 |
| chr5__165415490 | G | A | Blood_Tier3 | 0.500 |
| chr11__128142166 | T | G | Blood_Tier3 | 0.120 |
| chr2__18299562 | C | T | Blood_Tier3 | 0.138 |
| chr7__146112371 | A | C | Blood_Tier3 | 0.111 |
| chr3__54979489 | C | T | Blood_Tier2 | 0.189 |
| chr10__37533452 | C | T | Blood_Tier2 | 0.132 |
| chr10__19005639 | C | T | Blood_Tier3 | 0.133 |
| chr12__19795500 | A | G | Blood_Tier3 | 0.123 |
| chr5__46351383 | A | T | Blood_Tier3 | 0.154 |
| chr8__109849541 | G | A | Blood_Tier3 | 0.188 |
| chr7__57708912 | C | T | Blood_Tier3 | 0.082 |
| chr12__17994764 | A | T | Blood_Tier3 | 0.139 |
| chr17__72097012 | G | A | Blood_Tier3 | 0.152 |
| chr7__142112777 | T | A | Blood_Tier3 | 0.065 |
| chr22__20734031 | A | C | Blood_Tier3 | 0.200 |
| chr12__94310061 | C | T | Blood_Tier3 | 0.231 |
| chr4__55890156 | A | G | Blood_Tier3 | 0.200 |
| chr12__59590041 | C | T | Blood_Tier3 | 0.077 |
| chr6__72812320 | T | A | Blood_Tier3 | 0.239 |
| chrX__10342784 | G | A | Blood_Tier3 | 0.087 |
| chr14__106773919 | C | T | Blood_Tier3 | 0.050 |
| chr8__48253994 | A | T | Blood_Tier3 | 0.182 |
| chr5__109463919 | C | T | Blood_Tier3 | 0.200 |
| chr2__53347865 | A | G | Blood_Tier3 | 0.182 |
| chr12__81828865 | T | A | Blood_Tier3 | 0.300 |
| chr2__181402658 | A | G | Blood_Tier3 | 0.148 |
| chr8__41646406 | T | C | Blood_Tier3 | 0.228 |
| chr14__30443917 | G | T | Blood_Tier3 | 0.114 |
| chr5__25439865 | C | T | Blood_Tier3 | 0.167 |
| chr12__75466133 | A | G | Blood_Tier3 | 0.333 |
| chr18__28026822 | T | A | Blood_Tier3 | 0.087 |
| chr16__84230139 | C | T | Blood_Tier3 | 0.235 |
| chr16__80313752 | G | T | Blood_Tier3 | 0.064 |
| chr12__11201928 | C | A | Blood_Tier3 | 0.111 |
| chr7__142170774 | T | C | Blood_Tier3 | 0.068 |
| chr7__70914724 | A | T | Blood_Tier3 | 0.103 |
| chr22__33479147 | A | T | Blood_Tier3 | 0.133 |
| chr7__57701642 | A | T | Blood_Tier3 | 0.111 |
| chr3__193363447 | T | C | Blood_Tier3 | 0.267 |
| chr3__64061172 | G | A | Blood_Tier3 | 0.130 |
| chr4__128738035 | A | G | Blood_Tier3 | 0.231 |
| chr12__11210413 | T | A | Blood_Tier3 | 0.071 |
| chr6__157861588 | G | A | Blood_Tier3 | 0.265 |
| chr9__34743193 | C | T | Blood_Tier3 | 0.154 |
| chr9__85102481 | T | C | Blood_Tier3 | 0.500 |
| chr3__58299010 | A | G | Blood_Tier3 | 0.194 |
| chr2__208730681 | A | G | Blood_Tier3 | 0.182 |
| chr12__17164642 | C | A | Blood_Tier3 | 0.167 |
| chr11__102543946 | G | T | Blood_Tier3 | 0.143 |
| chr6__147163290 | T | C | Blood_Tier3 | 0.176 |
| chr7__142112781 | G | A | Blood_Tier3 | 0.069 |
| chr3__100305657 | A | T | Blood_Tier3 | 0.071 |
| chr5__66754035 | A | G | Blood_Tier3 | 0.176 |
| chr7__142112780 | A | C | Blood_Tier3 | 0.068 |
| chr5__148127300 | A | T | Blood_Tier3 | 0.113 |
| chr2__170983636 | T | C | Blood_Tier3 | 0.114 |
| chr22__44812734 | G | C | Blood_Tier3 | 0.200 |
| chr11__818321 | G | T | Blood_Tier3 | 0.172 |
| chr2__194109480 | A | C | Blood_Tier3 | 0.136 |
| chrX__22267527 | C | A | Blood_Tier3 | 0.231 |
| chr8__74400493 | T | C | Blood_Tier3 | 0.160 |
| chr2__194109479 | G | T | Blood_Tier3 | 0.109 |
| chr5__90035124 | A | G | Blood_Tier3 | 0.200 |
| chr13__71965717 | T | C | Blood_Tier3 | 0.400 |
| chr12__71818168 | T | C | Blood_Tier3 | 0.325 |
| chr13__105774795 | A | G | Blood_Tier3 | 0.194 |
| chr12__11208089 | T | C | Blood_Tier3 | 0.069 |
| chr8__126979801 | C | A | Blood_Tier3 | 0.160 |
| chr17__22217028 | A | G | Blood_Tier3 | 0.169 |
| chr16__85272383 | T | C | Blood_Tier3 | 0.207 |
| chr4__167260078 | T | A | Blood_Tier3 | 0.235 |
| chr14__78702011 | G | A | Blood_Tier3 | 0.194 |
| chr9__118893195 | A | C | Blood_Tier3 | 0.140 |
| chr1__26062022 | T | C | Blood_Tier3 | 0.375 |
| chr15__27169321 | T | C | Blood_Tier3 | 0.125 |
| chr3__159270357 | A | G | Blood_Tier3 | 0.214 |
| chr2__124948132 | A | T | Blood_Tier3 | 0.108 |
| chr12__95000294 | C | A | Blood_Tier3 | 0.348 |
| chr10__5854557 | G | A | Blood_Tier3 | 0.418 |
| chr2__89078968 | T | G | Blood_Tier3 | 0.130 |
| chr10__23074430 | T | C | Blood_Tier3 | 0.167 |
| chr8__41412171 | T | C | Blood_Tier3 | 0.133 |
| chr7__142174972 | G | A | Blood_Tier3 | 0.091 |
| chr4__177646963 | C | T | Blood_Tier3 | 0.231 |
| chr18__48949657 | A | G | Blood_Tier3 | 0.093 |
| chr11__41614000 | G | A | Blood_Tier3 | 0.318 |
| chr1__247287449 | T | C | Blood_Tier3 | 0.326 |
| chr6__1083453 | G | A | Blood_Tier3 | 0.148 |
| chr8__126968905 | T | C | Blood_Tier3 | 0.188 |
| chr6__107437250 | C | T | Blood_Tier3 | 0.250 |
| chr5__125454760 | A | T | Blood_Tier3 | 0.184 |
| chr9__79491520 | G | T | Blood_Tier3 | 0.275 |
| chr5__3489699 | A | G | Blood_Tier3 | 0.091 |
| chr8__34584827 | T | C | Blood_Tier3 | 0.150 |
| chrX__111336821 | G | C | Blood_Tier3 | 0.246 |
| chrX__61950819 | T | A | Blood_Tier3 | 0.092 |
| chr2__95585054 | T | A | Blood_Tier3 | 0.108 |
| chr9__132197219 | A | T | Blood_Tier3 | 0.048 |
| chr16__12463267 | A | T | Blood_Tier3 | 0.355 |
| chr19__22493999 | T | C | Blood_Tier3 | 0.167 |
| chr7__9011291 | A | G | Blood_Tier3 | 0.174 |
| chr10__8961853 | G | A | Blood_Tier3 | 0.167 |
| chr6__32601760 | A | G | Blood_Tier3 | 0.085 |
| chr14__27688768 | G | A | Blood_Tier3 | 0.226 |
| chr12__56932190 | T | C | Blood_Tier3 | 0.207 |
| chr13__58595985 | G | A | Blood_Tier3 | 0.231 |
| chr6__166140601 | T | G | Blood_Tier3 | 0.179 |
| chrX__105747658 | C | G | Blood_Tier3 | 0.311 |
| chr14__29818566 | T | C | Blood_Tier3 | 0.167 |
| chr7__151243817 | G | A | Blood_Tier3 | 0.250 |
| chr11__30101778 | T | C | Blood_Tier3 | 0.188 |
| chr2__133007221 | C | T | Blood_Tier3 | 0.094 |
| chr14__88428978 | A | C | Blood_Tier3 | 0.088 |
| chr17__55146827 | C | A | Blood_Tier3 | 0.246 |
| chr10__125358902 | A | G | Blood_Tier3 | 0.133 |
| chr13__81777002 | A | G | Blood_Tier3 | 0.214 |
| chr21__30330594 | G | C | Blood_Tier3 | 0.245 |
| chr17__16808692 | G | T | Blood_Tier3 | 0.288 |
| chr7__85996958 | A | T | Blood_Tier3 | 0.214 |
| chr1__73751623 | T | C | Blood_Tier3 | 0.200 |
| chr8__58904251 | G | A | Blood_Tier3 | 0.070 |
| chr1__46105145 | T | G | Blood_Tier3 | 0.118 |
| chr6__81335455 | T | C | Blood_Tier3 | 0.214 |
| chr4__133649937 | A | C | Blood_Tier3 | 0.143 |
| chr8__140603870 | T | G | Blood_Tier3 | 0.043 |
| chr20__26135983 | T | A | Blood_Tier3 | 0.075 |
| chr6__166593308 | T | A | Blood_Tier3 | 0.222 |
| chr1__248907327 | G | A | Blood_Tier3 | 0.071 |
| chr6__128779460 | C | T | Blood_Tier3 | 0.344 |
| chr3__120233618 | G | T | Blood_Tier3 | 0.194 |
| chrX__130838278 | T | G | Blood_Tier3 | 0.121 |
| chr16__33969302 | C | A | Blood_Tier3 | 0.076 |
| chr5__106638979 | T | C | Blood_Tier3 | 0.076 |
| chr14__106902786 | C | T | Blood_Tier3 | 0.115 |
| chr7__67019719 | C | T | Blood_Tier3 | 0.290 |
| chrX__71907477 | G | T | Blood_Tier3 | 0.167 |
| chr11__38061266 | A | G | Blood_Tier3 | 0.300 |
| chr11__16074445 | T | A | Blood_Tier3 | 0.181 |
| chr20__54408043 | C | A | Blood_Tier3 | 0.156 |
| chr18__57933503 | C | T | Blood_Tier3 | 0.340 |
| chr3__19741718 | T | C | Blood_Tier3 | 0.158 |
| chr4__72193370 | T | C | Blood_Tier3 | 0.211 |
| chr9__10009074 | A | G | Blood_Tier3 | 0.273 |
| chr12__7168848 | T | A | Blood_Tier3 | 0.105 |
| chr7__131727222 | A | C | Blood_Tier3 | 0.250 |
| chr1__196140095 | T | C | Blood_Tier3 | 0.167 |
| chr4__31280818 | G | A | Blood_Tier3 | 0.180 |

## ST2: Variant Allele Fractions of 307 Validated Somatic Mutations

| **gvarID** | **PB0** | **PB1** | **PB2** | **M1** | **G1** | **G2** | **B1** | **B2** | **T1** | **T4.2** | **T8.2** | **cluster** |
| --- | --- | --- | --- | --- | --- | --- | --- | --- | --- | --- | --- | --- |
| chr1__107865400 | 28.21 | 35.27 | 32.54 | 37.13 | 43.42 | 44.84 | 4 | 3.14 | 5.34 | 11.3 | 3.11 | 1 |
| chr1__113621193 | 31.34 | 35.99 | 36.29 | 42.39 | 46.41 | 46.99 | 4.28 | 3.06 | 6.53 | 11.74 | 4.19 | 1 |
| chr1__170805330 | 30.54 | 34.33 | 32.1 | 43.78 | 46.22 | 44.7 | 4.96 | 2.84 | 5.36 | 11.28 | 3.99 | 1 |
| chr1__179142889 | 29.6 | 36.1 | 33.77 | 37.7 | 45.95 | 43.76 | 4.23 | 4.01 | 6.74 | 12.09 | 4.27 | 1 |
| chr1__180644884 | 3.15 | 9.27 | 8.84 | 10.7 | 12.95 | 12.45 | 1.22 | 0.65 | 0.08 | 1.88 | 0.21 | 5 |
| chr1__180893060 | 30.51 | 34.84 | 31.6 | 43.76 | 43.46 | 44.62 | 4.12 | 3.25 | 5.55 | 11.48 | 3.15 | 1 |
| chr1__186581699 | 28.78 | 35.52 | 34.34 | 42.09 | 45.58 | 45.64 | 3.84 | 2.7 | 5.81 | 11.8 | 3.27 | 1 |
| chr1__201313101 | 1.97 | 7.02 | 7.39 | 9.06 | 9.74 | 10.6 | 1.17 | 0.83 | 0.15 | 1.46 | 0.1 | 5 |
| chr1__216608363 | 29.16 | 37.11 | 33.4 | 41.66 | 46.06 | 45.97 | 4.58 | 3.35 | 5.21 | 12.6 | 3.82 | 1 |
| chr1__222054608 | 19.99 | 25.98 | 26.63 | 31.72 | 35.93 | 34.97 | 2.92 | 2.63 | 2.96 | 7.28 | 1.4 | 2 |
| chr1__231042202 | 29.62 | 36.19 | 33.79 | 43.78 | 45.33 | 47.59 | 3.85 | 3.25 | 5.54 | 11.76 | 3.85 | 1 |
| chr1__237498066 | 28.34 | 33.46 | 31.94 | 38.2 | 44.08 | 44.34 | 3.75 | 2.81 | 4.51 | 10.98 | 3.1 | 1 |
| chr1__244390672 | 20.93 | 23.45 | 20.48 | 28.78 | 33.14 | 33.03 | 2.37 | 2.17 | 3.08 | 7.48 | 1.97 | 2 |
| chr1__35656873 | 9.53 | 9.44 | 8.76 | 12.07 | 13.78 | 11.55 | 0.7 | 1.14 | 0.42 | 1.92 | 0.56 | 4 |
| chr1__37164781 | 29.27 | 34.63 | 34.2 | 40.87 | 44.18 | 45.74 | 4.44 | 3.49 | 5.79 | 12.55 | 3.08 | 1 |
| chr1__48839483 | 28.86 | 31.94 | 30.64 | 38.08 | 44.58 | 43.04 | 3.68 | 2.77 | 5.27 | 10.46 | 3.25 | 1 |
| chr1__53590585 | 22.36 | 27.16 | 24.77 | 30.03 | 36.21 | 34.58 | 3.13 | 2.68 | 3.36 | 8.12 | 2.11 | 2 |
| chr1__7358343 | 29.16 | 34.35 | 33.19 | 37.68 | 45.04 | 43.57 | 4.29 | 3.83 | 5.61 | 10.86 | 3.51 | 1 |
| chr1__77567898 | 28.88 | 33.75 | 32.61 | 39.16 | 44.33 | 45.23 | 4.13 | 3.08 | 5.74 | 11.94 | 3.58 | 1 |
| chr1__79469387 | 31.69 | 32.6 | 32.05 | 37.89 | 43.79 | 43.38 | 4.32 | 2.91 | 5.84 | 10.24 | 3.36 | 1 |
| chr1__86284096 | 26.88 | 33.04 | 30.38 | 38.82 | 42.24 | 41.39 | 3.71 | 2.75 | 5.46 | 11.32 | 3.18 | 1 |
| chr10__109656568 | 30.09 | 33.73 | 33.18 | 41.62 | 45.61 | 45.75 | 5.26 | 2.59 | 5.89 | 11.79 | 3.69 | 1 |
| chr10__125225251 | 21.43 | 25.99 | 24.03 | 30.13 | 33.44 | 35.87 | 2.84 | 2.44 | 2.87 | 6.13 | 2.2 | 2 |
| chr10__126339325 | 27.18 | 28.21 | 25.73 | 32.38 | 36.45 | 37.93 | 2.3 | 3.63 | 4.45 | 9.32 | 2.94 | 2 |
| chr10__131873809 | 29.04 | 34.49 | 34.12 | 38.76 | 45.49 | 44.83 | 3.82 | 3.27 | 5.64 | 11.42 | 3.57 | 1 |
| chr10__20491854 | 2.66 | 7.45 | 8.59 | 10.58 | 10.82 | 12.57 | 0.74 | 0.59 | 0.15 | 1.26 | 0.41 | 5 |
| chr10__37533452 | 4.83 | 10.91 | 12.12 | 14.14 | 14.78 | 14.91 | 1.33 | 1.24 | 0.77 | 2.61 | 0.53 | 5 |
| chr10__55246987 | 27.81 | 33.1 | 31.01 | 38.72 | 42.41 | 41.44 | 3.54 | 2.68 | 5.49 | 8.8 | 3.51 | 1 |
| chr10__5854557 | 24.07 | 32.32 | 29.62 | 35 | 37.4 | 39.52 | 6.1 | 5.92 | 7.68 | 13.3 | 5.34 | 1 |
| chr10__58835229 | 28.79 | 35.62 | 29.78 | 38.67 | 43.9 | 43.73 | 3.18 | 3.79 | 5.71 | 10.84 | 3.43 | 1 |
| chr10__71833394 | 19.96 | 24.12 | 22.05 | 30.72 | 33.17 | 35.11 | 2.81 | 2.13 | 3.47 | 8.25 | 2.41 | 2 |
| chr10__84756078 | 28.56 | 33.89 | 32.63 | 40.51 | 46.65 | 45.07 | 3.48 | 2.81 | 6.41 | 12.22 | 2.61 | 1 |
| chr10__86358071 | 27.04 | 30.31 | 31.58 | 39.32 | 42.01 | 41.5 | 4.15 | 2.51 | 5.09 | 11.27 | 3.16 | 1 |
| chr10__93546985 | 25.7 | 31.3 | 29.29 | 37.52 | 41.81 | 40.67 | 3.17 | 2.82 | 4.37 | 9.85 | 2.83 | 1 |
| chr10__95590751 | 12.75 | 18.84 | 17.97 | 21.57 | 23.29 | 25.32 | 2.95 | 1.45 | 0.94 | 3.57 | 1.28 | 3 |
| chr11__102729686 | 29.74 | 36.25 | 32.97 | 40.88 | 43.64 | 42.88 | 4.01 | 3.45 | 5.43 | 11.34 | 3.36 | 1 |
| chr11__115664774 | 26.38 | 27.47 | 27.27 | 36.58 | 40.24 | 38.42 | 2.82 | 2.06 | 4.91 | 10.14 | 3.26 | 2 |
| chr11__119595518 | 28.13 | 34.83 | 33.12 | 39.53 | 43.97 | 44.29 | 4.1 | 3.11 | 5 | 12.1 | 3.15 | 1 |
| chr11__123959954 | 29.4 | 34.57 | 30.95 | 33.93 | 41.58 | 42.32 | 3.49 | 3.25 | 5.11 | 10.67 | 3.27 | 1 |
| chr11__133074959 | 27.4 | 31.87 | 29.03 | 35.28 | 40.79 | 41.82 | 3.6 | 2.64 | 4.82 | 10.14 | 2.38 | 1 |
| chr11__133213386 | 23.21 | 28.63 | 24.32 | 28.66 | 35.9 | 35.16 | 3.23 | 2.72 | 3.04 | 7.48 | 2.11 | 2 |
| chr11__134641812 | 21.06 | 25.85 | 26.24 | 27.72 | 34.02 | 34.4 | 3.35 | 2.93 | 3.31 | 7.37 | 2.64 | 2 |
| chr11__16074445 | 17.72 | 22.08 | 19.07 | 22.42 | 26.6 | 26.79 | 1.84 | 2.12 | 2 | 4.82 | 1.21 | 2 |
| chr11__17058370 | 30.35 | 34.81 | 33.52 | 41.09 | 44.48 | 47.15 | 3.77 | 3.87 | 5.3 | 12.23 | 4 | 1 |
| chr11__18060193 | 30.61 | 34.37 | 30.77 | 39.5 | 46.09 | 44.82 | 3.59 | 3.45 | 6.31 | 9.7 | 3.21 | 1 |
| chr11__24024971 | 29.46 | 35.36 | 29.5 | 38.02 | 44.33 | 46.11 | 3.28 | 3.71 | 4.86 | 11.91 | 3.12 | 1 |
| chr11__35956176 | 4.68 | 11.14 | 9.99 | 12.49 | 13.84 | 14.2 | 1.74 | 1.18 | 0.31 | 2 | 0.43 | 5 |
| chr11__37183794 | 3.47 | 9.92 | 9.67 | 12.88 | 11.33 | 12.99 | 0.97 | 0.6 | 0.07 | 1.35 | 0.15 | 5 |
| chr11__3766585 | 24.87 | 26.64 | 27.48 | 33.08 | 39.91 | 37.22 | 2.62 | 3.02 | 4.26 | 10.16 | 2.84 | 2 |
| chr11__41614000 | 16.21 | 21.2 | 17.18 | 25.84 | 28.54 | 26.92 | 2.91 | 1.43 | 3.07 | 6.56 | 1.03 | 3 |
| chr11__44366993 | 29.77 | 35.53 | 33.9 | 41.24 | 45.23 | 44.28 | 4 | 3.14 | 5.83 | 11.99 | 3.36 | 1 |
| chr11__55523417 | 24.31 | 29.54 | 28.43 | 32.52 | 37.82 | 38.97 | 3.42 | 2.48 | 5.23 | 8.89 | 2.77 | 2 |
| chr11__62037391 | 29.34 | 34.2 | 34.09 | 39.52 | 42.7 | 44.41 | 3.78 | 3.85 | 4.85 | 12.48 | 3.24 | 1 |
| chr11__82829818 | 28.28 | 30.89 | 31.2 | 37.51 | 42.65 | 42.95 | 3.82 | 3.95 | 6.14 | 12.6 | 3.36 | 1 |
| chr11__91653831 | 31.43 | 34.76 | 31.9 | 39.95 | 44.87 | 45.98 | 4.37 | 3.35 | 4.96 | 11.8 | 3.47 | 1 |
| chr11__94335617 | 28.11 | 37 | 30.99 | 39.33 | 44.87 | 44.14 | 3.94 | 2.79 | 5.93 | 10.92 | 3.83 | 1 |
| chr11__99367399 | 28.45 | 34.84 | 32.8 | 40.48 | 45.4 | 46.27 | 3.58 | 3.31 | 5.48 | 11.45 | 3.83 | 1 |
| chr12__14130513 | 2.91 | 8.88 | 8.92 | 11.35 | 11.43 | 13.34 | 0.81 | 0.67 | 0.16 | 1.33 | 0.33 | 5 |
| chr12__26365989 | 27.52 | 31.94 | 33.72 | 40.25 | 42.51 | 42.39 | 3.05 | 2.58 | 6.05 | 9.46 | 3.48 | 1 |
| chr12__29141533 | 28.64 | 34.47 | 32.11 | 41.18 | 45.01 | 45.4 | 3.71 | 2.32 | 5.59 | 11.52 | 3.71 | 1 |
| chr12__33853021 | 27.96 | 33.32 | 28.46 | 37.33 | 43.69 | 43.57 | 3.6 | 2.05 | 4.58 | 10.98 | 3.52 | 1 |
| chr12__48861370 | 13.25 | 15.74 | 13.36 | 18.24 | 21.86 | 19.19 | 0.91 | 0.87 | 2 | 4.32 | 1.04 | 3 |
| chr12__59238155 | 27.26 | 31.84 | 30.1 | 35.97 | 42.48 | 42.89 | 3.41 | 2.35 | 4.93 | 11.19 | 3.31 | 1 |
| chr12__59303326 | 27.42 | 36.52 | 34.14 | 39.73 | 46.63 | 45.16 | 2.96 | 2.41 | 5.83 | 11.76 | 3.33 | 1 |
| chr12__80759750 | 21.07 | 23.49 | 24.38 | 29.03 | 30.27 | 32.82 | 2.54 | 1.88 | 4.01 | 8.63 | 2.86 | 2 |
| chr12__86416207 | 30.29 | 34.23 | 33.21 | 38.28 | 45.07 | 45.09 | 3.46 | 2.12 | 5.97 | 11.89 | 3.36 | 1 |
| chr12__87948328 | 2.52 | 9.72 | 9.34 | 11.97 | 12.82 | 14.61 | 1 | 0.91 | 0.07 | 1.6 | 0.36 | 5 |
| chr12__90867035 | 23.6 | 27.43 | 26.34 | 32.95 | 35.47 | 36.68 | 2.78 | 2.24 | 3.05 | 7.65 | 2.18 | 2 |
| chr13__105958180 | 29.14 | 35.9 | 33.4 | 40.01 | 44.66 | 45.92 | 3.89 | 3.43 | 5.76 | 12.37 | 3.72 | 1 |
| chr13__112600346 | 23.47 | 28.57 | 29.2 | 32.59 | 39.32 | 38.64 | 2.21 | 2.49 | 1.8 | 5.56 | 2.44 | 2 |
| chr13__35027179 | 29.24 | 32.58 | 31.94 | 39.52 | 43.94 | 44.7 | 3.93 | 3.23 | 4.87 | 10.99 | 2.93 | 1 |
| chr13__57257821 | 28.06 | 35 | 31.86 | 39.49 | 42.29 | 43.45 | 3.03 | 2.5 | 5.1 | 10.69 | 3.83 | 1 |
| chr13__58595985 | 27.8 | 31.36 | 30.6 | 39.4 | 44.37 | 41.86 | 2.99 | 2.82 | 4.93 | 11.29 | 3.14 | 1 |
| chr13__81353501 | 3.04 | 8.47 | 9.04 | 10.49 | 10.74 | 12.7 | 0.73 | 0.69 | 0.17 | 1.37 | 0.18 | 5 |
| chr13__83427582 | 23.54 | 27.11 | 25.19 | 31.26 | 36.54 | 35.56 | 2.94 | 2.39 | 3.04 | 6.97 | 2.41 | 2 |
| chr13__97517961 | 24.14 | 27.96 | 25.71 | 32.4 | 37.25 | 38.39 | 2.71 | 2.35 | 2.7 | 7.78 | 2.18 | 2 |
| chr14__26630906 | 19.59 | 23.11 | 21.24 | 24.58 | 29.64 | 27.96 | 2.19 | 1.87 | 3.15 | 7.39 | 1.61 | 2 |
| chr14__31631966 | 28.13 | 33.01 | 31.84 | 37.83 | 45.13 | 42.02 | 3.54 | 2.98 | 5.53 | 11.27 | 4.09 | 1 |
| chr14__33343977 | 26.79 | 30.26 | 26.6 | 37.33 | 38.74 | 35.96 | 3.3 | 2.19 | 5.51 | 10.96 | 2.89 | 2 |
| chr14__35339308 | 20.94 | 25.77 | 24.86 | 31.85 | 35.01 | 36.01 | 3 | 2.24 | 2.79 | 7.9 | 2.32 | 2 |
| chr14__50105572 | 28.29 | 34.43 | 32.28 | 42.87 | 45.48 | 45.48 | 3.91 | 4.94 | 5.63 | 13.05 | 3.17 | 1 |
| chr14__64495445 | 29.31 | 34.59 | 34.06 | 36.96 | 43.37 | 47.11 | 3.96 | 3.99 | 5.48 | 11.8 | 3.6 | 1 |
| chr14__77817270 | 29.56 | 33 | 32.45 | 39.58 | 45.19 | 43.79 | 3.54 | 4 | 5.52 | 11.34 | 3.39 | 1 |
| chr14__78702011 | 27.15 | 31 | 29.71 | 38.14 | 40.72 | 42.55 | 4.05 | 2.97 | 4.75 | 10.37 | 2.99 | 1 |
| chr14__82551829 | 28.73 | 32.72 | 32.34 | 39.6 | 42.84 | 44.29 | 3.33 | 3.05 | 5.75 | 10.98 | 3.45 | 1 |
| chr14__93904956 | 27.63 | 34.2 | 32.82 | 40.18 | 44.13 | 44.6 | 3.99 | 3.65 | 5.25 | 11.12 | 3.68 | 1 |
| chr15__24051898 | 29.18 | 32.91 | 33.36 | 38.9 | 45.22 | 45.89 | 3.63 | 3.83 | 5.55 | 10.04 | 3.52 | 1 |
| chr15__36040233 | 19.86 | 25.15 | 25.03 | 30.68 | 36.92 | 33.62 | 2.48 | 2.05 | 3.31 | 6.6 | 2.28 | 2 |
| chr15__77477740 | 30.45 | 33.86 | 32.57 | 38.08 | 44.14 | 44.33 | 3.66 | 3.13 | 5.4 | 10.42 | 3.26 | 1 |
| chr15__86765022 | 2.62 | 8.99 | 7.98 | 11.73 | 12.47 | 13.32 | 1.19 | 0.63 | 0.19 | 1.66 | 0.29 | 5 |
| chr15__94305134 | 30.21 | 35.05 | 33.76 | 41.54 | 43.77 | 45.27 | 4.54 | 2.92 | 6.86 | 11.46 | 3.55 | 1 |
| chr16__15603964 | 29.61 | 35.53 | 32.67 | 40.33 | 45.05 | 43.48 | 3.63 | 3.42 | 5.36 | 11.99 | 3.61 | 1 |
| chr16__24450792 | 20.72 | 27.58 | 24.61 | 29.21 | 33.35 | 34.92 | 2.09 | 2.5 | 3.7 | 8.2 | 2.46 | 2 |
| chr16__26597017 | 3.08 | 8.99 | 8.12 | 10.38 | 11.65 | 13.7 | 0.84 | 0.57 | 0.17 | 1.18 | 0.32 | 5 |
| chr16__50955663 | 29.41 | 36.6 | 32.86 | 39.69 | 44.88 | 45.6 | 5.19 | 3.84 | 5.86 | 12.05 | 3.69 | 1 |
| chr16__53424032 | 33.14 | 40.97 | 38.09 | 45.76 | 49.52 | 49.04 | 3.62 | 3.59 | 6.01 | 13.69 | 3.12 | 1 |
| chr16__6613470 | 28.93 | 32.76 | 31.58 | 38.04 | 43.55 | 43.2 | 3.93 | 2.95 | 5.94 | 11.03 | 3.27 | 1 |
| chr16__7739039 | 13.87 | 21.03 | 22.03 | 25.2 | 27.92 | 29.58 | 2.27 | 1.38 | 1.04 | 4.67 | 1.02 | 3 |
| chr16__80313752 | 3.11 | 8.5 | 8.88 | 10.23 | 10.95 | 11.94 | 1.49 | 0.93 | 0.2 | 1.25 | 0.18 | 5 |
| chr16__87305621 | 29.06 | 33.6 | 33.99 | 39.53 | 45.56 | 44.68 | 4.46 | 4.02 | 6.15 | 12.69 | 4.24 | 1 |
| chr16__88255777 | 21.29 | 25.14 | 25.45 | 28.41 | 35.72 | 33.99 | 3.13 | 2.88 | 2.95 | 7.53 | 2.19 | 2 |
| chr17__31969569 | 33.04 | 39.89 | 37.43 | 45.68 | 51 | 48.65 | 4.27 | 2.73 | 5.92 | 12.25 | 3.41 | 1 |
| chr17__52417510 | 3.08 | 9.49 | 8.45 | 11.18 | 12.03 | 11.6 | 1.1 | 0.93 | 0.11 | 1.59 | 0.37 | 5 |
| chr17__57689194 | 25.27 | 30.43 | 29.95 | 34.74 | 42.71 | 39.49 | 3.1 | 2.48 | 4.75 | 8.88 | 2.87 | 2 |
| chr17__63146103 | 27.18 | 34.05 | 34.73 | 39.01 | 43.04 | 43.07 | 3.97 | 3.53 | 5.8 | 10.78 | 2.9 | 1 |
| chr17__66672231 | 28.86 | 35.83 | 32.28 | 37.83 | 43.85 | 43.15 | 3.67 | 3.4 | 5.91 | 11.47 | 2.77 | 1 |
| chr17__68940187 | 22.69 | 26.67 | 24.96 | 31.76 | 37.29 | 35.81 | 2.8 | 1.7 | 3.88 | 7.55 | 2.52 | 2 |
| chr17__72097012 | 14.42 | 21.99 | 20.68 | 26.55 | 30.05 | 28.31 | 2.92 | 1.5 | 1.73 | 5.99 | 0.87 | 3 |
| chr17__72851459 | 9.1 | 9.48 | 8.72 | 11.6 | 13.46 | 12.35 | 0.78 | 0.85 | 0.81 | 2.34 | 0.54 | 4 |
| chr17__8280395 | 29.8 | 35.18 | 32.57 | 39.54 | 44.75 | 43.13 | 3.62 | 3.34 | 5.84 | 13.53 | 3.32 | 1 |
| chr18__11198650 | 29.39 | 34.82 | 32.2 | 41.89 | 44.25 | 46.67 | 3.8 | 3.42 | 6.11 | 11.58 | 3.24 | 1 |
| chr18__1392644 | 28.64 | 35.02 | 34.26 | 42.68 | 45.2 | 43.07 | 4.26 | 3.3 | 5.5 | 12.46 | 3.75 | 1 |
| chr18__5194040 | 28.7 | 34.91 | 34.01 | 37.57 | 43.75 | 44.87 | 3.91 | 2.95 | 6.13 | 12.36 | 3.6 | 1 |
| chr18__70491670 | 29.5 | 34.47 | 32.94 | 40.68 | 43.7 | 45.31 | 3.84 | 3.28 | 5.11 | 11.95 | 3.85 | 1 |
| chr18__75948056 | 2.57 | 8.69 | 8.86 | 11.1 | 12.85 | 12.29 | 0.92 | 0.66 | 0.02 | 1.42 | 0.44 | 5 |
| chr19__29093087 | 30.33 | 35.23 | 35.03 | 40.76 | 46.43 | 46.77 | 4.11 | 3.09 | 5.97 | 11.94 | 3.98 | 1 |
| chr19__48149785 | 14.29 | 21.06 | 18.99 | 23.71 | 26.14 | 26.56 | 2.48 | 2.64 | 1.45 | 4.39 | 0.87 | 3 |
| chr19__56255629 | 30.07 | 31.64 | 30.55 | 40.17 | 43.71 | 46.46 | 3.5 | 3.02 | 4.86 | 11.11 | 3.4 | 1 |
| chr2__123856244 | 19.03 | 24.61 | 24.83 | 27.83 | 32.98 | 33.73 | 1.89 | 2.07 | 4.05 | 8.5 | 2.47 | 2 |
| chr2__141131460 | 29.71 | 35.01 | 33.15 | 40.13 | 45.17 | 45.93 | 3.87 | 2.91 | 6.02 | 11.13 | 2.93 | 1 |
| chr2__148198348 | 28.48 | 32.74 | 30.2 | 37.88 | 41.27 | 41.79 | 3.59 | 2.44 | 5.59 | 10.62 | 3.01 | 1 |
| chr2__157173187 | 28.62 | 33.08 | 32.89 | 40.11 | 44.73 | 45.92 | 3.53 | 3.11 | 4.62 | 12.42 | 3.3 | 1 |
| chr2__15721915 | 29.77 | 36.99 | 33.76 | 40.62 | 44.82 | 46.3 | 4.07 | 3.23 | 5.13 | 11.39 | 3.47 | 1 |
| chr2__164563143 | 29.53 | 33.8 | 31.9 | 39.81 | 43.6 | 44 | 3.12 | 3.35 | 4.89 | 10.96 | 3.75 | 1 |
| chr2__176211302 | 29.32 | 34.08 | 33 | 38.73 | 45.13 | 43.65 | 3.9 | 2.85 | 5.87 | 12.88 | 3.69 | 1 |
| chr2__183771999 | 31.32 | 35.92 | 33.39 | 41.66 | 46.91 | 45.96 | 4.13 | 3.29 | 6.53 | 11.88 | 4.1 | 1 |
| chr2__185184871 | 15.01 | 21.99 | 20.01 | 25.7 | 28.19 | 28.35 | 2.43 | 1.66 | 1.66 | 5.1 | 1.12 | 3 |
| chr2__194613483 | 27.72 | 34.44 | 32.51 | 38.8 | 44.26 | 45.29 | 4.49 | 2.62 | 5.87 | 11.18 | 3.36 | 1 |
| chr2__198609762 | 21.34 | 28.9 | 25.56 | 30.98 | 33.69 | 36.13 | 3.29 | 2.2 | 3.19 | 6.73 | 2.23 | 2 |
| chr2__199917885 | 29.25 | 35.05 | 32.41 | 40.36 | 47.22 | 45.68 | 4.74 | 3.04 | 6.27 | 11 | 3.85 | 1 |
| chr2__200482779 | 29.09 | 34.33 | 31.78 | 38.78 | 44.25 | 44.57 | 3.71 | 3.19 | 5.33 | 11.53 | 4.08 | 1 |
| chr2__203150320 | 31 | 34.02 | 33.94 | 42.51 | 45.81 | 45.27 | 3.16 | 3.55 | 5.56 | 12.72 | 2.97 | 1 |
| chr2__222988297 | 29.79 | 34.27 | 32.3 | 39.44 | 43.59 | 46.98 | 4.12 | 3.05 | 5.62 | 11.08 | 3.08 | 1 |
| chr2__227183785 | 25.83 | 30.86 | 28.82 | 37.07 | 40.85 | 40.62 | 3.43 | 3.09 | 4.37 | 8.75 | 2.44 | 1 |
| chr2__227317709 | 32.79 | 37.22 | 34.76 | 41.66 | 47.47 | 47.16 | 4.95 | 2.15 | 5.93 | 11.69 | 3.95 | 1 |
| chr2__228258480 | 31.29 | 37.06 | 35.72 | 39.71 | 46.56 | 46.37 | 4.42 | 3.54 | 5.45 | 11.62 | 4.56 | 1 |
| chr2__237158632 | 3.17 | 8.81 | 8.46 | 9.61 | 9.87 | 14.11 | 0.61 | 0.57 | 0.07 | 1.17 | 0.3 | 5 |
| chr2__28226905 | 30.62 | 33.84 | 28.53 | 36.42 | 40.54 | 38.6 | 3.23 | 2.75 | 6.22 | 11.48 | 2.76 | 1 |
| chr2__42979897 | 27.7 | 34.41 | 31.53 | 40.56 | 43.69 | 45.26 | 3.85 | 3.51 | 5.83 | 11.53 | 3.4 | 1 |
| chr2__61401283 | 31.83 | 35.52 | 33.48 | 42.15 | 45.38 | 47.6 | 3.67 | 2.88 | 5.63 | 11.39 | 3.97 | 1 |
| chr2__96516676 | 29.61 | 34.09 | 30.32 | 39.08 | 44.22 | 43.9 | 3.84 | 3.37 | 5.76 | 11.08 | 2.66 | 1 |
| chr2__99565440 | 27.33 | 33.33 | 32.56 | 38.3 | 44.08 | 44.26 | 3.63 | 2.26 | 4.75 | 12.12 | 2.7 | 1 |
| chr20__33824956 | 27.87 | 32.4 | 32.56 | 40.11 | 42.79 | 43.95 | 3 | 2.56 | 4.93 | 11.31 | 3.22 | 1 |
| chr20__36061512 | 30.95 | 35.53 | 35.38 | 43.45 | 47.53 | 47.69 | 4.63 | 3.25 | 6.09 | 13.99 | 3.85 | 1 |
| chr20__36457105 | 29.91 | 32.03 | 32.83 | 38.84 | 44.93 | 44.53 | 3.35 | 3.25 | 5.58 | 11.02 | 3.62 | 1 |
| chr20__38230941 | 27.93 | 32.43 | 30.4 | 38.32 | 41.14 | 44.62 | 3.21 | 3.21 | 4.94 | 10.57 | 3.27 | 1 |
| chr20__39862424 | 31.91 | 34.33 | 31.61 | 41.19 | 45.72 | 42.72 | 4.45 | 3.97 | 5.63 | 11.88 | 3.46 | 1 |
| chr20__40341458 | 14.75 | 20.06 | 19.52 | 24.81 | 28.19 | 26.87 | 2.09 | 1.59 | 1.09 | 4.73 | 1.14 | 3 |
| chr20__46878505 | 31.92 | 36.23 | 34.79 | 41.19 | 46.48 | 46.16 | 3.75 | 2.78 | 6.75 | 12.23 | 3.44 | 1 |
| chr20__53456479 | 29 | 33.18 | 28.86 | 40.98 | 42.97 | 44.8 | 3.39 | 2.44 | 6.28 | 10.78 | 3.27 | 1 |
| chr20__53722528 | 30.41 | 31.87 | 31.54 | 39.57 | 45.82 | 43.74 | 3.85 | 2.65 | 6.86 | 10.71 | 3.52 | 1 |
| chr20__54408043 | 28.38 | 35.75 | 33.39 | 38.37 | 43.88 | 46.41 | 4.57 | 3.42 | 5.59 | 11.96 | 3.53 | 1 |
| chr20__57909208 | 2.98 | 9.35 | 9.68 | 10.25 | 11.58 | 11.96 | 0.82 | 1.17 | 0.25 | 1.95 | 0.34 | 5 |
| chr21__27807421 | 28.74 | 33.06 | 33.12 | 39.42 | 42.55 | 43.35 | 4.53 | 3.58 | 5.58 | 11.87 | 3.75 | 1 |
| chr21__30330594 | 28.89 | 32.8 | 31.81 | 37.85 | 43.83 | 41.96 | 3.88 | 3.68 | 5.63 | 11.44 | 3.45 | 1 |
| chr21__35721670 | 30.15 | 33.71 | 33.78 | 41.01 | 43.76 | 44.36 | 4.28 | 3.17 | 5.62 | 11.65 | 3.71 | 1 |
| chr21__39764905 | 28.34 | 34.18 | 32.58 | 39.3 | 43.31 | 42.59 | 4.07 | 2.95 | 5.34 | 10.54 | 3.62 | 1 |
| chr21__41193413 | 22.97 | 29.03 | 28.32 | 32.63 | 39.49 | 37.12 | 4.45 | 3.27 | 4.73 | 9.92 | 3.32 | 2 |
| chr21__47278674 | 29.8 | 36.16 | 33.85 | 40.84 | 44.89 | 45.91 | 4.37 | 3.99 | 5.06 | 11.75 | 3.38 | 1 |
| chr22__34167420 | 30.13 | 34.8 | 32.33 | 42.24 | 45.2 | 45.24 | 4.12 | 2.81 | 6.37 | 11.9 | 2.91 | 1 |
| chr22__40724186 | 30.67 | 34.76 | 32.14 | 41.11 | 43.72 | 44.45 | 3.99 | 3.67 | 5.04 | 11.54 | 3.39 | 1 |
| chr22__44385037 | 31.46 | 36.29 | 32.41 | 41.89 | 47.97 | 47.43 | 3.43 | 5.06 | 5.96 | 11.48 | 3.44 | 1 |
| chr22__46538743 | 28.65 | 34.83 | 31.37 | 37.31 | 45.08 | 43.13 | 3.93 | 3.74 | 4.72 | 11.48 | 3.26 | 1 |
| chr3__130030623 | 30.26 | 34.7 | 32.4 | 41.25 | 46.08 | 43.96 | 3.69 | 3.22 | 6.17 | 11.46 | 3.87 | 1 |
| chr3__138121382 | 29.53 | 35.75 | 32.92 | 39.97 | 44.56 | 44.87 | 4.45 | 3.3 | 5.69 | 12.68 | 3.4 | 1 |
| chr3__147908859 | 29.2 | 33.75 | 32.27 | 40.88 | 43.01 | 44.56 | 3.99 | 3.1 | 5.51 | 10.83 | 3.82 | 1 |
| chr3__153752188 | 29.46 | 35.23 | 32.56 | 40.35 | 43.93 | 45.23 | 4.41 | 2.86 | 5.72 | 12.59 | 2.8 | 1 |
| chr3__158785637 | 14.34 | 22.11 | 20.72 | 24.98 | 27.04 | 29.21 | 2.12 | 1.51 | 1.68 | 5.6 | 0.96 | 3 |
| chr3__163911788 | 3.53 | 10.6 | 10.5 | 12.32 | 14.51 | 14.03 | 1.24 | 1.03 | 0.1 | 1.43 | 0.37 | 5 |
| chr3__176430705 | 30.56 | 35.45 | 35.24 | 40.5 | 47.04 | 46.91 | 4.19 | 2.95 | 5.84 | 12.5 | 3.09 | 1 |
| chr3__192399169 | 30.3 | 33.83 | 34.47 | 39.54 | 44.82 | 45.2 | 4.15 | 3.15 | 5.76 | 11.07 | 3.01 | 1 |
| chr3__192960239 | 29.7 | 34.3 | 31.21 | 40.77 | 45.19 | 44.98 | 3.38 | 2.92 | 5.69 | 12.05 | 3.28 | 1 |
| chr3__21941024 | 28.38 | 32.78 | 32.47 | 37.83 | 45.12 | 43.66 | 4.36 | 2.86 | 5.58 | 10.99 | 3.45 | 1 |
| chr3__37745882 | 2.29 | 7.28 | 6.94 | 7.52 | 9.18 | 9.64 | 0.76 | 0.69 | 0.05 | 0.79 | 0.25 | 5 |
| chr3__38961324 | 29.53 | 37 | 31.79 | 42.82 | 46.03 | 45.04 | 4 | 3.46 | 5.37 | 10.75 | 3.37 | 1 |
| chr3__50930603 | 20.94 | 25.12 | 24.99 | 28.31 | 33.72 | 33.95 | 3.12 | 2.31 | 2.06 | 6.54 | 2.11 | 2 |
| chr3__52315833 | 30.34 | 35.17 | 33.75 | 41.04 | 42.22 | 45.51 | 4.09 | 3.93 | 5.29 | 12.01 | 3.29 | 1 |
| chr3__52977475 | 27.19 | 33.45 | 31.29 | 40.26 | 43.97 | 43.04 | 3.34 | 4.15 | 5.79 | 11.43 | 3.19 | 1 |
| chr3__54633194 | 3.29 | 8.4 | 8.9 | 11.09 | 11.19 | 12.56 | 1.09 | 0.82 | 0.17 | 1.83 | 0.22 | 5 |
| chr3__54979489 | 12.66 | 18.81 | 19.18 | 23.13 | 25.43 | 25.93 | 1.43 | 1.36 | 1.12 | 3.59 | 0.86 | 3 |
| chr3__71590489 | 22.89 | 25.98 | 26.87 | 30.27 | 35.77 | 35.55 | 3.41 | 3.26 | 4.44 | 10.42 | 2.81 | 2 |
| chr3__94257342 | 28.83 | 32.16 | 32.05 | 38.16 | 41.96 | 43.41 | 4.07 | 3.21 | 4.63 | 10.1 | 3.19 | 1 |
| chr4__102280086 | 29.37 | 35.29 | 31.88 | 41.01 | 45.39 | 44.55 | 3.58 | 2.49 | 5.74 | 11.35 | 3.31 | 1 |
| chr4__115824013 | 7.43 | 7.53 | 6.74 | 9.04 | 10.15 | 10.14 | 0.75 | 0.42 | 0.77 | 1.63 | 0.6 | 4 |
| chr4__11638973 | 19.71 | 24.16 | 24.05 | 31.36 | 35.32 | 35.74 | 2.51 | 2.56 | 3.46 | 7.81 | 2.54 | 2 |
| chr4__123669774 | 29.68 | 37.29 | 32.31 | 42.82 | 45.1 | 42.06 | 3.97 | 2.36 | 5.66 | 12.53 | 3.82 | 1 |
| chr4__137409760 | 28.42 | 35.23 | 32.04 | 39.72 | 45.85 | 46.16 | 4.17 | 3.32 | 6.11 | 13.31 | 3.42 | 1 |
| chr4__145441371 | 29.22 | 33.45 | 33.41 | 41.12 | 45.02 | 43.01 | 4.34 | 3.98 | 4.9 | 11.48 | 3.1 | 1 |
| chr4__148542277 | 27.99 | 33.74 | 33.61 | 39.57 | 45.32 | 43.16 | 3.57 | 2.72 | 5.28 | 12.8 | 3.15 | 1 |
| chr4__149877106 | 28.61 | 34.18 | 30.71 | 39.38 | 43.32 | 44.77 | 3.44 | 3.22 | 6.01 | 11.12 | 3.39 | 1 |
| chr4__157045476 | 28.8 | 32.7 | 32.13 | 39.13 | 42.72 | 42.81 | 5.27 | 3.73 | 5.68 | 12.71 | 4.09 | 1 |
| chr4__159243915 | 28.61 | 33.54 | 30.3 | 41.21 | 42.35 | 43.33 | 4.07 | 3.04 | 5.13 | 10.12 | 3.09 | 1 |
| chr4__161063756 | 30.2 | 34.71 | 32.69 | 38.65 | 46.3 | 44.93 | 4.59 | 2.86 | 5.52 | 12.32 | 3.96 | 1 |
| chr4__169089352 | 28.44 | 33.4 | 32.78 | 37.89 | 45.36 | 43.06 | 4.01 | 3.19 | 5.29 | 11.8 | 2.76 | 1 |
| chr4__171058550 | 20.43 | 25.19 | 23.27 | 31.03 | 32.74 | 34.2 | 2.62 | 2.92 | 2.82 | 7.7 | 2.15 | 2 |
| chr4__17857259 | 29.81 | 35.1 | 33.04 | 38.35 | 43.31 | 43.78 | 4.06 | 3.39 | 4.96 | 11.28 | 2.96 | 1 |
| chr4__183386037 | 24.92 | 29.31 | 29.31 | 34.72 | 38.21 | 39.99 | 3.3 | 1.73 | 4.29 | 9.35 | 2.55 | 2 |
| chr4__24092455 | 15.28 | 22.94 | 20.58 | 24.68 | 27.82 | 29.29 | 1.94 | 1.89 | 1.73 | 5.17 | 0.83 | 3 |
| chr4__24761759 | 31.05 | 36.59 | 34.14 | 40.2 | 46.22 | 44.59 | 4.21 | 3.35 | 4.96 | 11.5 | 3.12 | 1 |
| chr4__30984482 | 28.94 | 33.92 | 30.57 | 39.76 | 43.02 | 42.95 | 3.48 | 2.83 | 5.03 | 11.22 | 3.73 | 1 |
| chr4__31280818 | 3.28 | 9.29 | 9.08 | 11.82 | 12.37 | 12.71 | 1.14 | 0.83 | 0.03 | 1.52 | 0.38 | 5 |
| chr4__55441256 | 27.42 | 34.59 | 32.69 | 35.99 | 43.43 | 43.23 | 3.29 | 2.5 | 5.5 | 11.21 | 3.91 | 1 |
| chr4__81241530 | 22.15 | 27.22 | 31.12 | 35.3 | 38.17 | 39.76 | 3.73 | 2.36 | 3.43 | 9.63 | 2.28 | 2 |
| chr5__123025785 | 30.08 | 34.73 | 34.2 | 40.84 | 45.25 | 45.7 | 4.33 | 3.84 | 4.5 | 12.52 | 3.49 | 1 |
| chr5__125454760 | 30.38 | 34.19 | 33.08 | 42.36 | 44.3 | 44.94 | 4.16 | 3.07 | 5.82 | 11.36 | 3.57 | 1 |
| chr5__130022910 | 30.26 | 36.17 | 35.84 | 40.98 | 45.23 | 47.36 | 4.14 | 3.63 | 5.76 | 12.7 | 3.12 | 1 |
| chr5__133385008 | 28.14 | 33.03 | 31.43 | 38.88 | 43.99 | 44.45 | 3.5 | 3.56 | 5.03 | 11.82 | 3.22 | 1 |
| chr5__142792230 | 28.18 | 36.47 | 32.09 | 41.78 | 44.17 | 45.55 | 4.66 | 2.95 | 6.66 | 11.62 | 3.59 | 1 |
| chr5__144949915 | 29.8 | 35.71 | 33.48 | 41.68 | 44.13 | 44.14 | 4.43 | 3.36 | 4.82 | 11.36 | 3.19 | 1 |
| chr5__148127300 | 3.02 | 8.44 | 7.53 | 11.53 | 11.57 | 13.41 | 1.06 | 0.87 | 0 | 1.53 | 0.38 | 5 |
| chr5__166164023 | 29.08 | 35.42 | 31.51 | 39.99 | 44.37 | 44.77 | 3.84 | 3.22 | 5.04 | 11.39 | 3.73 | 1 |
| chr5__166531089 | 33.08 | 38.34 | 35.12 | 45.36 | 46.89 | 48.41 | 4.11 | 3.34 | 5.98 | 11.76 | 3.99 | 1 |
| chr5__174418500 | 2.45 | 8.07 | 7.39 | 9.98 | 11.8 | 12.77 | 1.32 | 0.64 | 0.24 | 1.47 | 0.22 | 5 |
| chr5__175742143 | 30.77 | 35.34 | 34.23 | 42.81 | 46.39 | 46.36 | 4.2 | 3.95 | 5.94 | 12.49 | 3.3 | 1 |
| chr5__26329781 | 29.5 | 34.02 | 32.78 | 39.88 | 45.38 | 43.51 | 3.72 | 3.57 | 5.1 | 12.49 | 3.48 | 1 |
| chr5__34012260 | 20.5 | 25.33 | 23.68 | 29.43 | 35.22 | 33.09 | 2.49 | 2.23 | 2.95 | 6.65 | 2.42 | 2 |
| chr5__42290327 | 28.3 | 33.71 | 31.82 | 37.73 | 43.55 | 44.73 | 3.29 | 2.79 | 5.7 | 11.15 | 3.41 | 1 |
| chr5__84711280 | 2.75 | 8.04 | 7.93 | 10.95 | 11.74 | 10.86 | 1.51 | 0.76 | 0.07 | 1.41 | 0.5 | 5 |
| chr5__88243810 | 31.47 | 35.09 | 34.27 | 41.44 | 43.66 | 44.65 | 3.77 | 4.72 | 6.57 | 12.5 | 3.48 | 1 |
| chr6__100169580 | 28.57 | 33.28 | 31.18 | 40.63 | 43.94 | 44.81 | 4.16 | 2.89 | 5.34 | 12.57 | 3.07 | 1 |
| chr6__11618300 | 4.98 | 10.36 | 11.49 | 13.59 | 14.89 | 16.25 | 0.92 | 1.12 | 0.84 | 3.12 | 0.32 | 5 |
| chr6__121901998 | 3.2 | 8.9 | 9.21 | 11.39 | 12.51 | 13.18 | 0.88 | 1 | 0.14 | 1.28 | 0.27 | 5 |
| chr6__126113509 | 29.28 | 35.01 | 33.31 | 41 | 46.38 | 45.49 | 3.89 | 3.62 | 5.45 | 12.44 | 3.96 | 1 |
| chr6__128779460 | 28.4 | 32.06 | 31.25 | 39.42 | 43.4 | 44.96 | 3.46 | 3.04 | 5.05 | 11.57 | 3.32 | 1 |
| chr6__150816248 | 28.47 | 33.2 | 33.11 | 39.7 | 43.44 | 44 | 3.92 | 3.72 | 5.67 | 11.77 | 3.26 | 1 |
| chr6__152647273 | 28.01 | 32.09 | 32.73 | 37.74 | 43.53 | 42.43 | 3.65 | 3.64 | 6 | 10.34 | 3.08 | 1 |
| chr6__157861588 | 28.26 | 31.74 | 31.88 | 38.33 | 41.44 | 42.85 | 3.92 | 3.81 | 4.93 | 11.58 | 3.05 | 1 |
| chr6__160372588 | 30.28 | 34.47 | 32.34 | 39.9 | 44.57 | 44.6 | 3.89 | 3.95 | 5.45 | 13.05 | 3.16 | 1 |
| chr6__16390236 | 28.32 | 36.47 | 33.13 | 41.76 | 45.6 | 45.6 | 4.35 | 3.99 | 5.48 | 12.44 | 3.84 | 1 |
| chr6__166226981 | 9.22 | 21.15 | 22.68 | 26.39 | 28.55 | 30.58 | 2.1 | 2.5 | 1.11 | 4.56 | 0.6 | 3 |
| chr6__20794608 | 30.17 | 34.05 | 31.9 | 38.64 | 43.9 | 45.27 | 3.57 | 3.29 | 6.5 | 11.33 | 3.62 | 1 |
| chr6__28105508 | 32.37 | 38.31 | 38.32 | 41.86 | 44.8 | 48.2 | 4.31 | 4.25 | 4.63 | 13.85 | 3.34 | 1 |
| chr6__44324229 | 27.85 | 32.97 | 31.68 | 41.22 | 46.02 | 43.41 | 4.17 | 3.79 | 4.8 | 12.84 | 3.42 | 1 |
| chr6__51587361 | 10.3 | 10.57 | 9.17 | 11.44 | 14.03 | 13 | 0.88 | 1.07 | 0.79 | 2.19 | 0.56 | 4 |
| chr6__69085881 | 28.26 | 32.4 | 30.57 | 37.91 | 42.97 | 41.79 | 3.86 | 2.6 | 5.14 | 11.4 | 3.39 | 1 |
| chr6__72812320 | 27.34 | 31.88 | 33.15 | 39.3 | 40.2 | 42.97 | 4.07 | 2.67 | 5.15 | 10.75 | 3.18 | 1 |
| chr6__79616543 | 29.32 | 33.45 | 31.4 | 41.71 | 44.72 | 43.99 | 4.11 | 2.59 | 6.27 | 11.68 | 3.75 | 1 |
| chr6__81591019 | 14.27 | 16.73 | 18.85 | 23.89 | 25.16 | 24.95 | 2.08 | 0.99 | 2.82 | 5.6 | 1.98 | 3 |
| chr7__126483539 | 29.51 | 33.12 | 31.32 | 39.65 | 44.28 | 43.3 | 3.78 | 2.38 | 4.99 | 10.61 | 2.77 | 1 |
| chr7__129772266 | 4.18 | 9.57 | 10.31 | 11.88 | 13.73 | 13.98 | 1.91 | 1.56 | 0.89 | 3.06 | 1.16 | 5 |
| chr7__130645986 | 29.99 | 34.99 | 34.55 | 41.43 | 45.98 | 45.17 | 3.8 | 4.09 | 6.11 | 12.51 | 2.94 | 1 |
| chr7__131572995 | 30.5 | 35.56 | 33.52 | 40.71 | 44.7 | 44.87 | 4.59 | 2.22 | 5.78 | 11.52 | 3.23 | 1 |
| chr7__141023239 | 28.01 | 33.65 | 31.77 | 39.71 | 47.41 | 46.08 | 3.29 | 3.62 | 5.92 | 11.61 | 2.81 | 1 |
| chr7__14288496 | 6.35 | 7.82 | 6.89 | 9.54 | 8.35 | 9.8 | 1.25 | 1.06 | 2.47 | 4.32 | 0.94 | 4 |
| chr7__153381781 | 15.43 | 20.27 | 21 | 24.99 | 28.93 | 28.97 | 2.37 | 1.31 | 1.49 | 4.73 | 0.95 | 3 |
| chr7__24212517 | 29.09 | 34.4 | 31.32 | 40.36 | 44.23 | 44.52 | 3.27 | 2.79 | 5.38 | 11.6 | 3.64 | 1 |
| chr7__29217270 | 31.6 | 35.16 | 34.5 | 40.14 | 46.59 | 46.32 | 4.09 | 3.05 | 4.87 | 11.74 | 4.02 | 1 |
| chr7__40599736 | 29.98 | 34.71 | 32.42 | 40.21 | 44.5 | 46.04 | 3.96 | 2.91 | 5.37 | 11.07 | 3.89 | 1 |
| chr7__43402191 | 3.33 | 9.05 | 8.88 | 11.39 | 12.62 | 12.47 | 0.92 | 0.95 | 0.16 | 1.32 | 0.26 | 5 |
| chr7__54540043 | 27.53 | 33.49 | 31.97 | 39.73 | 44.52 | 43.01 | 4.15 | 2.46 | 5.3 | 11.14 | 3.82 | 1 |
| chr7__67019719 | 30.25 | 34.84 | 33.08 | 39.23 | 45.28 | 44.62 | 3.78 | 3.39 | 5.42 | 12.56 | 3.23 | 1 |
| chr7__78431782 | 25.98 | 29.1 | 29.51 | 34.19 | 39.09 | 40.4 | 3.25 | 2.96 | 4.24 | 10.56 | 3.93 | 2 |
| chr7__82293864 | 31.45 | 37.08 | 32.72 | 39.52 | 48.56 | 44.75 | 4.56 | 3.49 | 5.8 | 11.71 | 3.68 | 1 |
| chr7__83446721 | 29.57 | 34.73 | 32.45 | 39.98 | 45.09 | 43.9 | 4.39 | 3.32 | 5.49 | 10.74 | 3.81 | 1 |
| chr8__109617037 | 27.95 | 36.04 | 30.15 | 40.22 | 44.41 | 44.09 | 3.88 | 3.21 | 5.96 | 12.08 | 3.61 | 1 |
| chr8__11185286 | 28.45 | 34.25 | 32.98 | 42.16 | 43.46 | 44.47 | 3.71 | 4.33 | 5.44 | 11.85 | 3.19 | 1 |
| chr8__126819256 | 29.63 | 35.77 | 33.57 | 40.76 | 43.84 | 41.83 | 3.97 | 3.43 | 4.75 | 11.33 | 3.13 | 1 |
| chr8__13478301 | 29.27 | 34.61 | 32.9 | 41.34 | 43.89 | 44.55 | 3.9 | 3.23 | 4.94 | 11.8 | 3.67 | 1 |
| chr8__138529288 | 29.34 | 36.46 | 33.14 | 39.72 | 45.41 | 46.92 | 4.05 | 3.23 | 5.71 | 11.15 | 4.05 | 1 |
| chr8__141389872 | 27.16 | 33.22 | 29.86 | 39 | 42.36 | 43.67 | 4.09 | 3.2 | 4.92 | 11.47 | 3.66 | 1 |
| chr8__17407813 | 25.29 | 31.31 | 28.48 | 36.34 | 41.22 | 41.13 | 3.7 | 3.06 | 4.58 | 9.38 | 3.3 | 1 |
| chr8__27484960 | 21.49 | 27.91 | 23.38 | 31.03 | 33.55 | 33.22 | 2.96 | 2.6 | 2.83 | 7.16 | 2.33 | 2 |
| chr8__28291900 | 29.68 | 35.4 | 29.98 | 39.71 | 44.92 | 44.68 | 3.22 | 3.63 | 5.27 | 11.53 | 3.32 | 1 |
| chr8__34502821 | 30.46 | 32.15 | 34.93 | 42.06 | 45.31 | 46.57 | 3.8 | 3.25 | 5.35 | 10.43 | 3.46 | 1 |
| chr8__41646406 | 30.18 | 32.74 | 31.48 | 38.91 | 43.58 | 41.86 | 3.2 | 3.1 | 4.68 | 10.41 | 4.19 | 1 |
| chr8__41745949 | 27.51 | 32.81 | 30.72 | 39.82 | 43.88 | 42.68 | 4.47 | 3.77 | 5.81 | 11.83 | 4.06 | 1 |
| chr8__47359871 | 29.19 | 34.44 | 33.36 | 39 | 42.66 | 44.57 | 3.67 | 3.6 | 5.94 | 11.78 | 3.64 | 1 |
| chr8__54468375 | 29.9 | 34.19 | 32.15 | 38.59 | 45.44 | 44.3 | 3.99 | 3.09 | 5.96 | 11.58 | 3.25 | 1 |
| chr8__69906311 | 2.87 | 9.31 | 9.28 | 9.98 | 11.07 | 13.24 | 1.02 | 0.87 | 0.16 | 1.6 | 0.38 | 5 |
| chr8__99588635 | 29.17 | 35.46 | 33.83 | 41.41 | 42.21 | 44.24 | 4.19 | 2.94 | 5.56 | 12.2 | 3.62 | 1 |
| chr9__100480485 | 27.06 | 32.62 | 31.07 | 38.2 | 43.94 | 41.6 | 3.17 | 3.66 | 5.26 | 10.6 | 3.4 | 1 |
| chr9__118123925 | 27.66 | 34.04 | 30.96 | 38.54 | 43.46 | 46.14 | 4.31 | 2.94 | 4.91 | 11.01 | 3.17 | 1 |
| chr9__120691738 | 18.99 | 24.6 | 23.64 | 27.99 | 32.9 | 31.14 | 3.34 | 2.57 | 2.18 | 5.92 | 1.98 | 2 |
| chr9__125193632 | 29.24 | 32.34 | 33.31 | 38.85 | 45.62 | 42.9 | 3.46 | 3 | 5.6 | 12.78 | 4.1 | 1 |
| chr9__13732822 | 27.89 | 35.12 | 33.39 | 40.76 | 45.92 | 46.57 | 4.51 | 3.56 | 5.32 | 10.6 | 3.83 | 1 |
| chr9__137585155 | 29.11 | 35.31 | 30.56 | 40.61 | 41.81 | 43.49 | 5.18 | 3.06 | 4.15 | 10.94 | 3.39 | 1 |
| chr9__19516480 | 30.45 | 35.55 | 33.72 | 40.29 | 44.35 | 44.8 | 5.14 | 2.76 | 6.04 | 12.45 | 4.01 | 1 |
| chr9__37342120 | 33.77 | 40.88 | 39.3 | 45.85 | 51.98 | 52.99 | 5.22 | 4.21 | 6.7 | 14.77 | 4.68 | 1 |
| chr9__79491520 | 24.87 | 28.63 | 26.17 | 33.35 | 38.62 | 36.33 | 2.45 | 2.83 | 4.93 | 9.84 | 2.91 | 2 |
| chr9__84717849 | 29 | 34.62 | 32.5 | 40.13 | 44.1 | 44.15 | 3.47 | 3.44 | 5.8 | 12.87 | 3.99 | 1 |
| chr9__8517886 (PTPRD) | 30.3 | 34.74 | 32.49 | 41.74 | 45.59 | 42.76 | 4.8 | 3.57 | 5.41 | 12.55 | 3.81 | 1 |
| chr9__86052888 | 30.95 | 36.37 | 33.87 | 41.12 | 44.97 | 45.7 | 3.82 | 3.77 | 4.42 | 10.64 | 3.41 | 1 |
| chr9__88013515 | 27.25 | 34.3 | 30.16 | 38.58 | 43.81 | 42.16 | 3.75 | 3.16 | 5.42 | 10.28 | 2.83 | 1 |
| chrX__105747658 | 30.04 | 34.85 | 34.45 | 41.13 | 43.36 | 45.22 | 4.74 | 2.7 | 6.49 | 12.83 | 4.23 | 1 |
| chrX__107726986 | 23.53 | 27.28 | 25.5 | 31.68 | 37.54 | 37.26 | 2.71 | 2.34 | 2.86 | 8.23 | 2.08 | 2 |
| chrX__109490016 | 31.68 | 35.39 | 32.47 | 42.72 | 48.07 | 44.21 | 4.06 | 3.28 | 6.48 | 12.58 | 3.92 | 1 |
| chrX__111336821 | 23.67 | 29.07 | 26.17 | 31.18 | 33.23 | 36.05 | 2.84 | 2.87 | 3.2 | 7.26 | 2.36 | 2 |
| chrX__114116246 | 30.17 | 35.06 | 34.54 | 41.08 | 47.36 | 47.08 | 3.86 | 3.19 | 6.57 | 12.03 | 3.96 | 1 |
| chrX__123798197 | 22.06 | 27.08 | 24.68 | 28.65 | 32.77 | 34.65 | 2.26 | 1.89 | 3.43 | 5.66 | 1.95 | 2 |
| chrX__126808708 | 27.48 | 34.95 | 32.34 | 37.14 | 42.89 | 42.36 | 3.95 | 2.55 | 5.78 | 10.84 | 3.37 | 1 |
| chrX__126940482 | 28.87 | 36.64 | 32.31 | 39.78 | 45.63 | 42.26 | 5.05 | 2.73 | 6.03 | 11.31 | 3.91 | 1 |
| chrX__129344146 | 29.96 | 35.69 | 31.49 | 40.74 | 44.56 | 42.95 | 3.55 | 4.05 | 5.89 | 12.86 | 3.81 | 1 |
| chrX__130195968 | 2.91 | 10.03 | 9.86 | 12.68 | 12.38 | 13.76 | 1.38 | 0.99 | 0.25 | 1.46 | 0.26 | 5 |
| chrX__132397792 | 22.8 | 27.37 | 24.69 | 32.74 | 34.81 | 36.66 | 3.7 | 2.51 | 4.09 | 8.63 | 1.77 | 2 |
| chrX__136332201 | 2.65 | 9.25 | 10.11 | 11.74 | 11.31 | 13.15 | 0.83 | 1.06 | 0.17 | 1.37 | 0.3 | 5 |
| chrX__137615166 | 31.61 | 35.32 | 31.87 | 41.42 | 43.82 | 45.35 | 4.02 | 3.97 | 7.36 | 12.42 | 2.56 | 1 |
| chrX__147904819 | 27.18 | 34.36 | 31.32 | 37.92 | 43.71 | 42.17 | 3.77 | 3.16 | 6.09 | 11.11 | 4.06 | 1 |
| chrX__14888241 | 30.35 | 36.6 | 32.98 | 39.57 | 42.66 | 43.77 | 4.34 | 2.47 | 5.89 | 12.85 | 3.57 | 1 |
| chrX__14959214 | 5.59 | 5.64 | 6.32 | 7.36 | 8.26 | 6.56 | 0.71 | 0.5 | 3 | 4.41 | 0.89 | 4 |
| chrX__24604702 | 29.3 | 35.99 | 33.19 | 40.53 | 43.55 | 43.55 | 3.66 | 3.69 | 5.52 | 12.36 | 3.44 | 1 |
| chrX__34047538 | 27.19 | 36.3 | 36.47 | 40.56 | 46.01 | 44.69 | 4.4 | 3.35 | 6.45 | 12.99 | 4.55 | 1 |
| chrX__34206854 | 2.41 | 7.89 | 7.64 | 8.82 | 9.43 | 11 | 0.6 | 0.44 | 0.05 | 1.29 | 0.09 | 5 |
| chrX__34276891 | 31.76 | 34.88 | 33.11 | 40.85 | 46 | 47.12 | 4.61 | 3.14 | 6.23 | 11.42 | 4.58 | 1 |
| chrX__66876515 | 29.25 | 35.92 | 31.33 | 39.6 | 44.36 | 43.2 | 3.19 | 3.18 | 6.35 | 13.1 | 4.07 | 1 |
| chrX__84392105 | 29.93 | 35.7 | 36.37 | 41.51 | 44.03 | 44.34 | 4.13 | 3.29 | 6.48 | 12.66 | 3.98 | 1 |
| chrX__97811601 | 3.64 | 9.38 | 9.47 | 11.44 | 11.9 | 13.1 | 1.11 | 0.9 | 0.21 | 1.56 | 0.26 | 5 |
| chrX__99060469 | 28.76 | 35.23 | 34.28 | 38.61 | 44.02 | 44.97 | 3.51 | 3.1 | 6.53 | 12.51 | 3.98 | 1 |

## ST3: Candidate driver definitions

| ***GENE***  **[TRANSCIPT]** | **INCLUSION CRITERIA** |
| --- | --- |
|  |  |
| *DNMT3A*  [NM_022552] | **RTV:** frameshift\|nonsense\|splice-site  **KHV:** F290I\|F290C\|V296M\|P307S\|P307R\|R326H\|R326L\|R326C\|R326S\|G332R\|G332E\|V339A\|V339M\|V339G\|L344Q\|L344P\|R366P\|R366H\|R366G\|A368T\|A368V\|R379H\|R379C\|I407T\|I407N\|I407S\|F414L\|F414S\|F414C\|A462V\|K468R\|C497G\|C497Y\|Q527H\|Q527P\|Y533C\|S535F\|C537G\|C537R\|G543A\|G543S\|G543C\|L547H\|L547P\|L547F\|M548I\|M548K\|G550R\|W581R\|W581G\|W581C\|R604Q\|R604W\|R635W\|R635Q\|S638F\|G646V\|G646E\|L653W\|L653F\|I655N\|V657A\|V657M\|R659H\|Y660C\|V665G\|V665L\|M674V\|R676W\|R676Q\|G685R\|G685E\|G685A\|D686Y\|D686G\|R688H\|G699R\|G699S\|G699D\|P700L\|P700S\|P700R\|P700Q\|P700T\|P700A\|D702N\|D702Y\|V704M\|V704G\|I705F\|I705T\|I705S\|I705N\|G707D\|G707V\|C710S\|C710Y\|S714C\|V716D\|V716F\|V716I\|N717S\|N717I\|P718L\|R720H\|R720G\|K721R\|K721T\|Y724C\|R729Q\|R729W\|R729G\|F731C\|F731L\|F731Y\|F731I\|F732del\|F732C\|F732S\|F732L\|E733G\|E733A\|F734L\|F734C\|Y735C\|Y735N\|Y735S\|R736H\|R736C\|R736P\|L737H\|L737V\|L737F\|L737R\|A741V\|P742P\|P743R\|P743L\|R749C\|R749L\|R749H\|R749G\|F751L\|F751C\|F752del\|F752C\|F752L\|F752I\|F752V\|W753G\|W753C\|W753R\|L754P\|L754R\|L754H\|F755S\|F755I\|F755L\|M761I\|M761V\|G762C\|V763I\|S770L\|S770W\|S770P\|R771Q\|F772I\|F772V\|L773R\|L773V\|E774K\|E774D\|E774G\|I780T\|D781G\|R792H\|W795C\|W795L\|G796D\|G796V\|N797Y\|N797H\|N797S\|P799S\|P799R\|P799H\|R803S\|R803W\|P804L\|P804S\|K826R\|S828N\|K829R\|T835M\|N838D\|K841Q\|Q842E\|P849L\|D857N\|W860R\|E863D\|F868S\|G869S\|G869V\|M880V\|S881R\|S881I\|R882H\|R882P\|R882C\|R882G\|A884P\|A884V\|Q886R\|L889P\|L889R\|G890D\|G890R\|G890S\|V895M\|P896L\|V897G\|V897D\|R899L\|R899H\|R899C\|L901R\|L901H\|P904L\|F909C\|P904Q\|A910P\|C911R\|C911Y |
| *TET2*  [NM_001127208] | **RTV:** frameshift\|nonsense\|splice-site  **KHV:** missense_mutations_in_catalytic_domains_(p.1104-1481_and_1843-2002) |
| *ASXL1*  [NM_015338] | **RTV:** frameshift\|nonsense\|splice-site (only in exon 11-12)  **KHV:** none |
| *TP53*  [NM_001126112] | **RTV:** frameshift\|nonsense\|splice-site  **KHV:** S46F\|G105C\|G105R\|G105D\|G108S\|G108C\|R110L\|R110C\|T118A\|T118R\|T118I\|S127F\|S127Y\|L130V\|L130F\|K132Q\|K132E\|K132W\|K132R\|K132M\|K132N\|F134V\|F134L\|F134S\|C135W\|C135S\|C135F\|C135G\|C135Y\|Q136K\|Q136E\|Q136P\|Q136R\|Q136L\|Q136H\|A138P\|A138V\|A138A\|A138T\|T140I\|C141R\|C141G\|C141A\|C141Y\|C141S\|C141F\|C141W\|V143M\|V143A\|V143E\|L145Q\|W146C\|W146L\|L145R\|V147G\|P151T\|P151A\|P151S\|P151H\|P151R\|P152S\|P152R\|P152L\|T155P\|T155A\|V157F\|R158H\|R158L\|A159V\|A159P\|A159S\|A159D\|A161T\|A161D\|Y163N\|Y163H\|Y163D\|Y163S\|Y163C\|K164E\|K164M\|K164N\|K164P\|H168Y\|H168P\|H168R\|H168L\|H168Q\|M169I\|M169T\|M169V\|E171K\|E171Q\|E171G\|E171A\|E171V\|E171D\|V172D\|V173M\|V173L\|V173G\|R174W\|R175G\|R175C\|R175H\|C176R\|C176G\|C176Y\|C176F\|C176S\|P177R\|P177R\|P177L\|H178D\|H178P\|H178Q\|H179Y\|H179R\|H179Q\|R181C\|R181Y\|D186G\|G187S\|P190L\|P190T\|H193N\|H193P\|H193L\|H193R\|L194F\|L194R\|I195F\|I195N\|I195T\|R196P\|V197L\|G199V\|Y205N\|Y205C\|Y205H\|D208V\|R213Q\|R213P\|R213L\|R213Q\|H214D\|H214R\|S215G\|S215I\|S215R\|V216M\|V217G\|Y220N\|Y220H\|Y220S\|Y220C\|E224D\|I232F\|I232N\|I232T\|I232S\|Y234N\|Y234H\|Y234S\|Y234C\|Y236N\|Y236H\|Y236C\|M237V\|M237K\|M237I\|C238R\|C238G\|C238Y\|C238W\|N239T\|N239S\|S241Y\|S241C\|S241F\|C242G\|C242Y\|C242S\|C242F\|G244S\|G244C\|G244D\|G245S\|G245R\|G245C\|G245D\|G245A\|G245V\|G245S\|M246V\|M246K\|M246R\|M246I\|N247I\|R248W\|R248G\|R248Q\|R249G\|R249W\|R249T\|R249M\|P250L\|I251N\|L252P\|I254S\|I255F\|I255N\|I255S\|L257Q\|L257P\|E258K\|E258Q\|D259Y\|S261T\|G262D\|G262V\|L265P\|G266R\|G266E\|G266V\|R267W\|R267Q\|R267P\|E271K\|V272M\|V272L\|R273S\|R273G\|R273C\|R273H\|R273P\|R273L\|V274F\|V274D\|V274A\|V274G\|V274L\|C275Y\|C275S\|C275F\|A276P\|C277F\|C277Y\|P278T\|P278A\|P278S\|P278H\|P278R\|P278L\|G279E\|R280G\|R280K\|R280T\|R280I\|R280S\|D281N\|D281H\|D281Y\|D281G\|D281E\|R282G\|R282W\|R282Q\|R282P\|E285K\|E285V\|E286G\|E286V\|E286K\|K320N\|L330R\|G334V\|R337C\|R337L\|A347T\|L348F\|T377P |
| *JAK2*  [NM_004972] | **RTV:** none  **KHV:** N533D\|N533Y\|N533S\|H538R\|K539E\|K539L\|I540T\|I540V\|V617F\|R683S\|R683G\|del/ins537-539L\|del/ins538-539L\|del/ins540-543MK\|del/ins540-544MK\|del/ins541-543K\|del542-543\|del543-544\|ins11546-547 3717 |
| *SF3B1*  [NM_012433] | **RTV:** none  **KHV:** G347V\|R387W\|R387Q\|E592K\|E622D\|Y623C\|R625L\|R625C\|R625G\|H662Q\|H662D\|T663I\|K666N\|K666T\|K666E\|K666R\|K700E\|V701F\|A708T\|G740R\|G740E\|A744P\|D781G\|E783K\|R831Q\|L833F\|E862K\|R957Q |
| *GNB1*  [NM_002074] | **RTV:** none  **KHV**: K57N\|K57M\|K57E\|K57T\|I80T\|I80N |
| *CBL*  [NM_005188] | **RTV:** none  **KHV**: RING_finger_missense_p.381_421 |
| *SFRS2*  [NM_003016] | **RTV:** none  **KHV**: Y44H\|P95H\|P95L\|P95T\|P95R\|P95A\|P107H\|P95fs |
| *GNAS*  [NM_016592] | **RTV:** none  **KHV**: R201(844)S\|R201(844)C\|R201(844)H\|R201(844)L\|Q227(870)K\|Q227(870)R\|Q227(870)L\|Q227(870)H\|R374(1017)C |
| *BRCC3*  [NM_024332] | **RTV**: frameshift\|nonsense\|splice-site  **KHV**: none |
| *CREBBP*  [NM_004380] | **RTV**: frameshift\|nonsense\|splice-site  **KHV**: D1435E\|R1446L\|R1446H\|R1446C\|Y1450C\|P1476R\|Y1482H\|H1487Y\|W1502C\|Y1503D\|Y1503H\|Y1503F\|S1680del |
| *NRAS*  [NM_002524] | **RTV**: none  **KHV**: G12S\|G12R\|G12C\|G12N\|G12P\|G12Y\|G12D\|G12A\|G12V\|G12E\|G13S\|G13R\|G13C\|G13N\|G13P\|G13Y\|G13D\|G13A\|G13V\|G13E\|G60E\|G60R\|Q61R\|Q61L\|Q61K\|Q61P\|Q61H\|Q61Q |
| *RAD21*  [NM_006265] | **RTV**: frameshift\|nonsense\|splice-site  **KHV**: R65Q\|H208R\|Q474R |
| *U2AF1*  [NM_006758] | **RTV**: none  **KHV**: D14G\|S34F\|S34Y\|R35L\|R156H\|R156Q\|Q157R\|Q157P |
| *PPM1D**  [NM_003620] | **RTV**: frameshift\|nonsense\|splice-site (only in exon 5 or 6)  KHV: none |

**Table ST3: Gene-specific variant inclusion criteria for 16 genes recurrently mutated in elderly patients:** Definitions are taken from Jaiswal *et al*.^6^ . RTV: Rare Truncating Variant: a non-specific annotation indicating a type of truncating event, i.e.: ‘frameshift’, ‘nonsense’, ‘splice-site’. Recurrent truncating events in hematological malignancies are annotated as KHV. KHV: Known Hotspot Variant: Variants recurrently reported to be mutated in hematological malignancies.

Supplementary References

## References

1. Holstege H, *et al.* Somatic mutations found in the healthy blood compartment of a 115-yr-old woman demonstrate oligoclonal hematopoiesis. Genome Res. 2014 May;24(5):733-42. doi: <https://doi.org/10.1101/gr.162131.113>
2. Welch JS *et al.* The origin and evolution of mutations in acute myeloid leukemia. Cell. 2012 Jul 20;150(20):264-78. doi: <http://doi.org/10.1016/j.cell.2012.06.023>
3. Lee-Six H, *et al.* Population dynamics of normal human blood inferred from somatic mutations. Nature 2018 Sep;561(7724):473-478. doi: <https://doi.org/10.1038/s41586-018-0497-0>
4. Osorio F G, *et al.* Somatic Mutations Reveal Lineage Relationships and Age-Related Mutagenesis in Human Hematopoiesis. Cell Rep. 2018 Nov 27; 25(9): 2308–2316.e4. doi: https://10.1016/j.celrep.2018.11.014
5. Alexandrov LB, *et al.* Clock-like mutational processes in human somatic cells. Nat Genet. 2015 Dec;47(12):1402-7. doi: 10.1038/ng.3441. doi: <https://doi.org/10.1038/ng.3441>
6. Jaiswal S, *et al.* Clonal Hematopoiesis and Risk of Atherosclerotic Cardiovascular Disease. N Engl J Med. 2017 Jul 13;377(2):111-121. doi: <https://doi.org/10.1056/NEJMoa1701719>
7. Busque L, *et al.* Recurrent somatic TET2 mutations in normal elderly individuals with clonal hematopoiesis. Nat Genet. 2012 Nov;44(11):1179-81. doi: <https://doi.org/10.1038/ng.2413>
8. Xie M, *et al.* Age-related mutations associated with clonal hematopoietic expansion and malignancies. Nat Med. 2014 Dec;20(12):1472-8. doi: <https://doi.org/10.1038/nm.3733>
9. Jaiswal S, *et al.* Age-related clonal hematopoiesis associated with adverse outcomes. N Engl J Med. 2014 Dec 25;371(26):2488-98. doi: <https://doi.org/10.1056/NEJMoa1408617>
10. Genovese G, *et al.* Clonal hematopoiesis and blood-cancer risk inferred from blood DNA sequence. N Engl J Med. 2014 Dec 25;371(26):2477-87. doi: <https://doi.org/10.1056/NEJMoa1409405>
11. van den Akker EB, *et al.* Uncompromised 10-year survival of oldest old carrying somatic mutations in DNMT3A and TET2. Blood. 2016 Mar 17;127(11):1512-5. doi: <https://doi.org/10.1182/blood-2015-12-685925>
12. Schoenmaker M, *et al.* Evidence of genetic enrichment for exceptional survival using a family approach: the Leiden Longevity Study. Eur J Hum Genet. 2006 Jan;14(1):79-84. doi: <https://doi.org/10.1038/sj.ejhg.5201508>
13. Holstege H, *et al.* The 100-plus Study of cognitively healthy centenarians: rationale, design and cohort description. Eur J Epidemiol. 2018 Dec;33(12):1229-1249. doi: https://doi.org/10.1007/s10654-018-0451-3
14. McKenna A, *et al.* The Genome Analysis Toolkit: a MapReduce framework for analyzing next-generation DNA sequencing data. Genome Res. 2010 Sep;20(9):1297-303. doi: <https://doi.org/10.1101/gr.107524.110>
15. Blokzijl F, et al. MutationalPatterns: comprehensive genome-wide analysis of mutational processes. Genome Med. 2018 Apr 25;10(1):33. [doi:https://doi.org/ 10.1186/s13073-018-0539-0](https://doi.org/)
16. Miller MA, *et al.* SciClone: inferring clonal architecture and tracking the spatial and temporal patterns of tumor evolution. PLoS Comput Biol. 2014 Aug 7;10(8):e1003665. doi: <https://doi.org/10.1371/journal.pcbi.1003665>
17. Niknafs N, *et al.* SubClonal Hierarchy Inference from Somatic Mutations: Automatic Reconstruction of Cancer Evolutionary Trees from Multi-region Next Generation Sequencing. PLoS Comput Biol. 2015 Oct 5;11(10):e1004416. doi: <https://doi.org/10.1371/journal.pcbi.1004416>
18. van Zelm MC, *et al.* Replication history of B lymphocytes reveals homeostatic proliferation and extensive antigen-induced B cell expansion. J Exp Med. 2007 Mar 19;204(3):645-55. doi: <https://doi.org/10.1084/jem.20060964>
19. van der Weerd K, *et al.* Combined TCRG and TCRA TREC analysis reveals increased peripheral T-lymphocyte but constant intra-thymic proliferative history upon ageing. Mol Immunol. 2013 Mar;53(3):302-12. doi: <https://doi.org/10.1016/j.molimm.2012.08.019>
20. Shapiro MB, *et al.* RNA splice junctions of different classes of eukaryotes: sequence statistics and functional implications in gene expression. Nucleic Acids Res. 1987 Sep 11;15(17):7155-74. doi: <https://doi.org/10.1093/nar/15.17.7155>
21. Rentzsch P, et al., CADD: predicting the deleteriousness of variants throughout the human genome. Nucleic Acids Res. 2018 Oct 29. doi: https://doi.org/10.1093/nar/gky1016.
22. Veeriah S, et al.. The tyrosine phosphatase PTPRD is a tumor suppressor that is frequently inactivated and mutated in glioblastoma and other human cancers. Proc Natl Acad Sci U S A. 2009 Jun 9;106(23):9435-40. doi: https://doi.org/10.1073/pnas.0900571106
23. Nik-Zainal S, et al. Mutational processes molding the genomes of 21 breast cancers. Cell. 2012 May 25;149(5):979-93. doi: <https://doi.org/10.1016/j.cell.2012.04.024>
24. Alexandrov LB, et al. Deciphering Signatures of Mutational Processes Operative in Human Cancer. Cell Rep. 2013 Jan 31;3(1):246-59. doi: <https://doi.org/10.1016/j.celrep.2012.12.008.>
